# Novel approach to delivering pro-environmental messages significantly shifts norms and motivation, but children are not more effective spokespeople than adults

**DOI:** 10.1371/journal.pone.0255457

**Published:** 2021-09-08

**Authors:** Cynthia McPherson Frantz, John Petersen, Kathryn Lucaites

**Affiliations:** 1 Department of Psychology and Environmental Studies, Oberlin College, Oberlin, OH, United States of America; 2 Department of Environmental Studies and Biology, Oberlin College, Oberlin, OH, United States of America; 3 Department of Psychology, Oberlin College, Oberlin, OH, United States of America; Ball State University, UNITED STATES

## Abstract

Three studies provided initial laboratory tests of the effectiveness of a novel form of community-based environmental messaging intended to be deployed on public digital signs. In all studies, adult participants watched a slideshow of “Community Voices,” a display that combines community images and quotes to celebrate and empower pro-environmental and pro-community thought and action. In addition to assessing the general efficacy of the approach, a central goal was to assess the impact of alternative messengers by comparing identical text associated with either adult or child messengers (Studies 1, 2, and 3). We also assessed the impact of alternative framing of the message itself by comparing: injunctive vs non-injunctive wording (Study 1), political vs non-political content (Study 1), and future vs. present-oriented framing (Study 2). Studies 1 and 2 were conducted on a national sample. In addition, to assess the impact of local vs. non-local messengers, Study 3 compared the response of a non-local sample to a local population in which subjects had personal connections with the people and places featured in the message content. Exposure to Community Voices messages resulted in significant increases in social norm perception, concern about environmental issues, commitment to action, and optimism, suggesting that this approach to messaging is potentially valuable for stimulating cultural change. However, messages attributed to child messengers were generally not more effective, and in some cases were less effective than the same message attributed to adults. We also found no significant difference in the impact of the alternative message frames studied.

## Introduction

Climate change and other environmental threats require an “all hands on deck” approach; a resilient and sustainable response requires large scale and concurrent shifts in culture, politics, economics, and technology in a short time span. Social norms are a potentially effective and scalable leverage point [1]: People are powerfully influenced by what they believe other people think and do, particularly people that they see as “like them” [2, 3]. A potentially valuable community-based mechanism for using social norms to drive positive cultural change is to identify positive thoughts and actions that are already taking place within a particular community, and then communicate these back to the community as a whole [4]. The goal of such an approach is to reinforce thought, consumptive choices, and political action that build sustainability and resilience in communities. However, the efficacy of implementing such an approach through simple public messaging has not been well tested and raises a number of important questions and challenges regarding how to best frame these messages so as to maximize the desired impact.

This research provides an initial proof-of-concept test of the efficacy of “Community Voices” (CV), a novel approach to social marketing that combines localized images with message content generated from community members. Such content can be displayed in a variety of ways, but has been developed in particular to make use of the ubiquitous venue of digital signage (electronic screens in public places that show rotating content). The explicit goal of Community Voices is to shift cultural norms and inspire pro-environmental and pro-social action. The studies reported here test whether exposure to Community Voices in a controlled setting can create the desired shifts. We also examined the effectiveness of alternative approaches to framing and delivering the same general messages. Specifically, we assessed the impact of alternative messengers and alternative framing of the messages on several psychological attributes.

### Community voices

Community Voices (CV) is a novel approach to conveying pro-environmental and pro-social messaging content for display on digital signage. The approach draws content, in the form of quotes paired with photographs, from local community members and can be easily replicated in a variety of communities and contexts. This technology has been operational in the City of Oberlin OH (population 8,300) since 2015 and has since been deployed on a pilot basis in Cleveland OH and in three other college-town communities. The Oberlin version can be viewed online (URL: www.environmentaldashboard.org/community-voices). The methods used to develop CV content as well as the technology have been described in detail elsewhere [5]. In brief, the psychological goal of CV is to discover, communicate and thereby strengthen pro-environmental and pro-community thought, identity and action. Most people are already engaged in at least some sort of pro-environmental thought and action in their daily lives that can serve as the basis for developing positive social norms. CV is explicitly designed to build and reinforce these norms to help move communities towards the goal of sustainability and resilience. CV also explicitly embraces diversity to highlight and leverage the unique history and character of a particular community so as to foster pride in accomplishments and encourage further aspiration.

**Fig 1:**
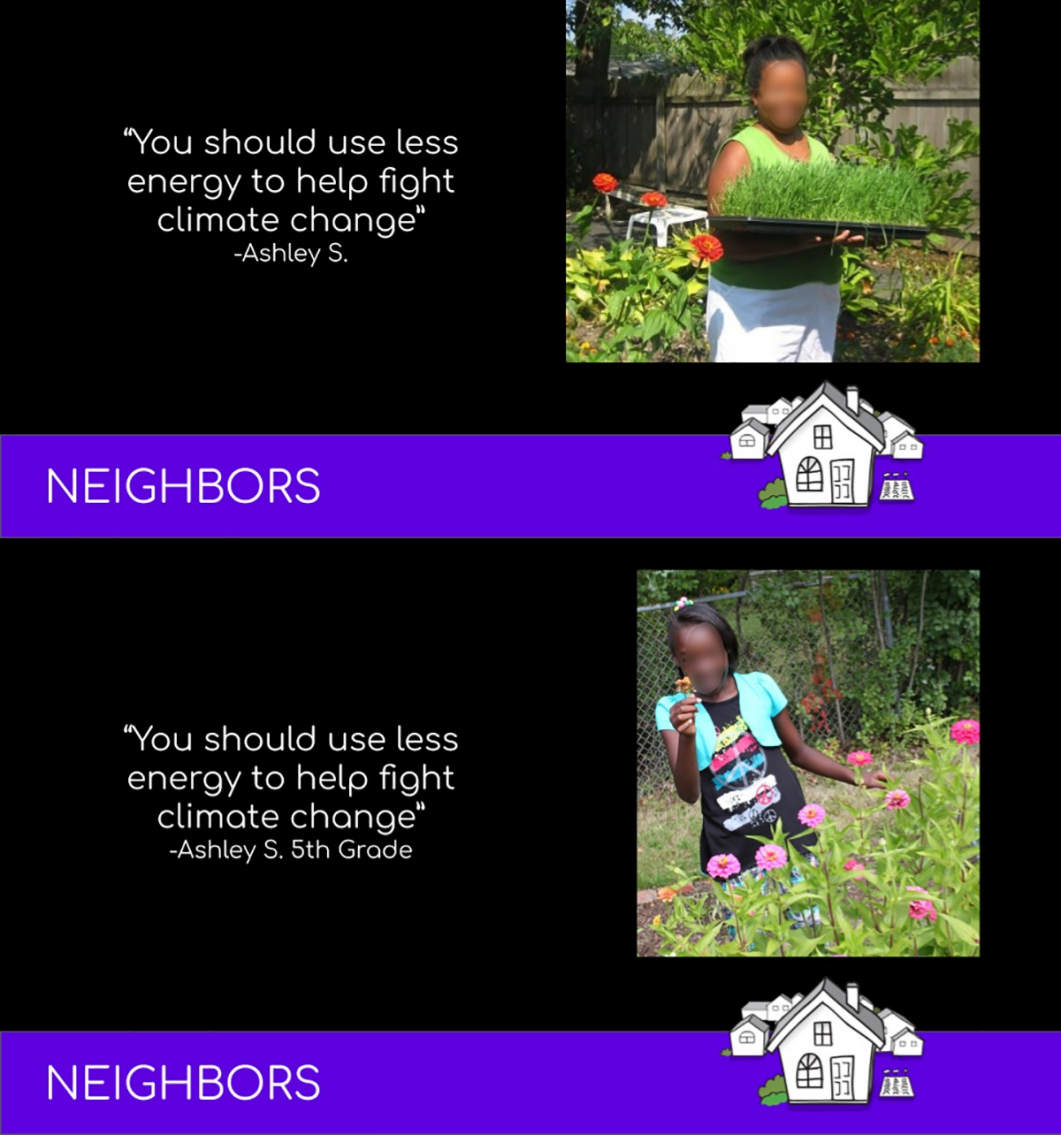
Example of alternative slide content used in different experimental groups. The top panel is one of 16 slides included in the set developed for the experimental condition receiving a combination of injunctive framing of messages attributed to adults. The bottom slide was part of the set delivered to the experimental condition receiving injunctive framing of messages attributed to children.

The Community Voices “slides, which are intended for display on digital signs installed in communities, combine images, text and category branding (Fig 1). The text content is developed through interviews, historic archives, and public documents. Images are contributed by community members or extracted from historic archives and photo shoots. Slides combining images and text are categorized and branded with iconography to highlight desirable thought and action occurring within different contexts. The Oberlin implementation includes six content categories: neighborhoods (branded as “Neighbors”), businesses (“Our Downtown”), historical legacy (“Heritage”), natural and cultivated beauty (“Natural”), public service (“Serving Our Community”) and the role of youth (“Next Generation”). Each slide is a combination of a photograph, quoted text, citation to source and title or role of source quoted, and category title and iconography. Although the text is drawn from interviews, those constructing the slides have flexibility in selecting what material to quote and therefore how the messages delivered are framed.

The communication approaches taken in CV content are based on literature drawn from research in social psychology and marketing and communication (e.g. [2, 6–9]). Eight principles derived from this literature have been used to inform the development of interview questions and the selection of text and image content. Specifically, content is developed to: focus on stories that are personal, local and emphasize connections; celebrate positive thought and action; feature cultural diversity; leverage social norms and satisfy people’s desire to belong; feature commitments and goals; emphasize positive consistency in thought and action; appeal to self-interest, convenience and personal health as well as community interest; use attention-grabbing images and wording. These principles reflect research indicating that environmental messaging is more effective when it is "nested in the cultural values and beliefs of the audience and … integrated with the experiential meaningfulness of place” [10 p43].

Although there is considerable research supporting the eight principles used to develop Community Voices content, the general question of whether this approach affects social norms had not been tested. Furthermore, there are many remaining questions with respect to the relative efficacy of different ways of framing messages and linking text to messengers. The studies reported here were designed to address the general question of whether CV content influences perceptions of social norms, concern about environmental issues, and commitment to take action. The studies were also designed to assess specific hypotheses about the impact of message framing and messenger attribution. The three general questions we asked and motivating rationale are explained in the sections that follow. The third question related to framing considers three alternative approaches to message framing.

#### Q1. Does exposure to community voices shift social norms, environmental concern, and commitment to act?

The first goal of these studies was to test whether exposure to Community Voices content does in fact shift social norms, as well as thoughts, feelings, and intentions around environmental issues. Social norms have proven to be an effective and versatile method of shifting pro-environmental behavior in many [3, 11, 12] but not all (e.g. [13]) applications. Further, once norms get established, they tend to be self-reinforcing; this means that an initial short-term investment of resources and effort can result in lasting behavior change (e.g. [14, 15]). Social norms can be *descriptive* (a message conveying what most people are thinking and doing), or injunctive (a message conveying what people *should* do [16]; there is empirical support that both kinds of norms promote pro-environmental behavior [17, 18]. The Community Voices approach primarily focuses on establishing descriptive norms by simply making pro-environmental thought and action in the community visible. However, in Study 1 we tested the impact of framing messages injunctively.

A norm-based approach has the potential to address two obstacles to establishing a culture of environmental stewardship. One important challenge with many environmental issues is that they can be characterized as “public goods” problems. In this situation, the benefits of individual action accrue to the public as a whole while the costs of actions are borne by the individual. Furthermore, the direct benefits of individual actions in large-scale public goods problems such as climate change are so dilute that they may undermine a sense of self-efficacy [19–21]. In this type of situation use of social norms to motivate behavior change is likely to be effective while appeals to self-interest are not. Making pro-environmental behavior in a community more visible not only helps to establish a norm, but may also enhance self-efficacy, a sense of responsibility, and optimism, thereby making individual and collective action more likely [19].

A second important challenge with environmental issues relates to the visibility of thought and action. Some pro-environmental behaviors are public: curbside recycling, biking and walking, and shopping at local farmers’ markets. However, many are not. For example, friends and neighbors usually do not know what each other’s thermostats are set to, how much effort they take to minimize shower length, whether they have insulated and weatherized their homes, whether they write or call elected officials, or whether they purchase carbon offsets for airplane travel. What’s more, environmental concerns related to public goods are often excluded from daily discourse; at least in the United States, at this point in history, most people do not regularly voice their concerns or actions related to climate change or environmental degradation at work or among friends [22].

For the reasons described above, we hypothesized that exposure to Community Voices would shift social norms, environmental concern, and commitment to act in a pro-environmental direction.

#### Q2. Are children more effective than adults for motivating concern and action on environmental issues?

Concern about climate change is a key predictor of personal and collective action to mitigate and adapt to the problem [23]. Once a consensus was reached within the scientific community on the reality of anthropogenic climate change, many scientists and professional communicators assumed that effective delivery of this scientific information would raise this concern. However, misinformation and disinformation have undermined the perceived credibility of the scientific consensus [24]. A recent meta-analysis of prior studies found that political affiliation and ideology are by far the most powerful predictors of belief in climate change–far more important in determining belief than either scientific knowledge or personal experience with extreme weather events [23]. However, climate change perceptions in children are less influenced by socio-ideological background [25]. This has led to the suggestion that children may be particularly useful messengers for delivering climate and other potentially controversial messages to care-givers and other adults [26]. Consistent with this hypothesis, a recent study found that children’s impact on climate views of their parents are most pronounced within socio-ideological groups that are most resistant to climate change communication [27].

A variety of evidence suggests that children are effective influencers of adult thought and behavior, at least in certain contexts. For example, children are widely targeted in advertising because both academic research [28–32] and trade communications [33, 34] support the idea that children influence the purchasing behavior of their adult caregivers. There is also a growing body of research on intergenerational learning that demonstrates that education delivered to children can influence the attitudes, knowledge and behaviors of adult care-givers on a range of health and social issues [e.g. 35–38]. Controlled studies indicate that education delivered to children can enhance care-givers’ thought and behavior on a variety of environmental issues including: knowledge of wildlife conservation principles [39]; knowledge of wetland ecology and household water management [26]; energy conservation behaviors [40]; participation in recycling programs [41]; as well as concern regarding climate change [27].

What is less clear is whether children may have similar impacts outside of a care-giver relationship. In the research cited above children are conduits of information to caregivers. We could find no research that tested child messengers speaking directly to non-caregiver adults. We discuss below arguments both for and against the effectiveness of children as spokespeople under these conditions.

There is a long history of using the future world that today’s children will inhabit as a means of motivating adults to make particular decisions in the public realm. An example is the iconographic "daisy" attack ad from the 1964 U.S. presidential campaign, which has been credited for helping Lyndon Johnson defeat Barry Goldwater [42]. In general, it has been recognized that children have important symbolic significance in social movements [43], and have the potential to do so in the fight against climate change [44]. Thus their messages may impact adults because they symbolize future generations that will be most impacted by the choices adults make now.

Additionally, research indicates that messengers are more likely to persuade if they are likable [2]; children are typically seen as endearing and likable. Finally, research on helping behavior suggests that perceptions of deservingness [45, 46] and feelings of empathy [47] elicit helping behavior. Once again children may be seen as particularly deserving protection, and may also elicit empathy and therefore protective behavior.

Children may also be particularly effective at delivering injunctive (“ought”) messages or in taking a stand on issues that are otherwise framed as political, as they may be viewed as less threatening, less likely to be pushing a particular agenda, and more trustworthy than adults. Messages from children that have political implications (for example supporting legislation that addresses climate change) may be perceived as less ideologically motivated and more personally motivated, and therefore have more impact on those who are less sympathetic to belief in or action on climate change or other politicized environmental issues. Again, there is experimental evidence to support the hypothesis that children (particularly daughters) are effective at convincing ideologically resistant parents (particularly fathers) to be concerned about climate change [48].

On the other hand, it is easy to identify countervailing arguments that lead to the conclusion that children in general might be less credible messengers than adults, at least in some circumstances. For example, children may be perceived as naive and as having less knowledge and expertise with respect to both problems and potential solutions (e.g., viewers might legitimately question what children know about the complicated factors influencing climate or the economics of renewable energy or installation of home solar panels). Or, they may be perceived as parroting what they have been told by adults, and thus pushing someone else’s agenda. A lack of similarity between messenger and audience is another credible reason to predict that child messengers will not be effective with adult audiences [49, 50].

Despite reasons to question the effectiveness of child messengers, we hypothesized that they would be more effective than adults under certain conditions (described below).

#### Q3. Does the framing of the message affect response? If so, does this differ depending on whether messages are attributed to children or adults?

The same general message can be worded in many different ways; research on framing suggests that these choices are likely to affect how the message is received [51] and the subsequent choices people make (e.g. [52, 53]). This is particularly true with respect to climate change [54, 55]. We chose to examine the impact of the following four alternative message frames and to consider how these might differ in impact when attributed to child vs. adult messengers.

#### Q3a. Injunctive versus non-injunctive

Given the magnitude of climate change impacts, environmentalists often frame action as an absolute necessity—for example we “must” or “ought” to act now. Whether this is objectively true or not, it is important to determine how this framing might impact the effectiveness of the message. On the one hand, [56] and [57] found injunctive norm-based interventions more effective than descriptive norm-based interventions. On the other hand, people are less likely to reliably pursue a goal if they feel that this goal has been thrust on them by others [58], and in fact may actively pursue an opposing goal in an attempt to re-establish a sense of freedom [59]. If children are seen as less threatening and more deserving of protection, they may be more effective at delivering such messages without sounding preachy or controlling. We hypothesized that an injunctive frame would be more effective when the message was attributed to children.

#### Q3b. Political vs. non-political

The scope of current environmental problems is so large that addressing them will require action on the political level (as opposed to focusing solely on individual behavior). However, although the impacts of climate change such as increased drought, fire, the intensity and frequency of storms will affect all aspects of society, the issue has become politically polarized [23]. Might a message framed in a political context be less effective as a result? For example, would the statement “our government needs to do something about climate change” be more off-putting than “you should use less energy to help fight climate change”? Once again, we hypothesized that children might feel less threatening, or be perceived as having less of an agenda, than adults in encouraging political action, and therefore would be more effective at delivering political messaging than adults.

#### Q3c. Future vs. present-oriented

Environmental problems have both current impacts and future impacts. Which framing is more impactful? One of the challenges of communicating about many environmental problems is that of temporal discounting: because the impacts feel far away the rewards of taking action can seem less valuable. Therefore, taking environmental action becomes less of a priority than addressing nearer term issues [60]. Environmental problems such as climate change also create a classic split incentive scenario: the costs of taking action are borne by this generation, while the benefits of taking action are experienced by future generations [61]. There is evidence in the health domain that future-oriented messages are often less effective than messages that emphasize the present [62, 63]. Consequently, some researchers [60] argue that messages about climate change that are framed in the present will be most effective. In contrast, others [64, p. 246] argue that communicators “should adopt techniques that increase individuals’ affinity and identification with future generations”.

Hendrick and Nicolaij [65] note that sizable portions (30–50%) of participants in studies they reviewed do not show classic temporal discounting effects on environmental issues, and conclude that the assessment of environmental risks differs from other risks because of the importance of ethical concerns. Since children will inhabit a future that extends beyond adults, we hypothesized that children would be more effective than adults at activating ethical concerns when delivering messages that emphasize protection of future conditions.

#### Q3d. Local vs. non-local messengers and place

Many [e.g. 10, 66] have documented the importance of place. Adams and Gynneld go so far as to say that "the experience of place is so central to being human that attempts to alter environmental attitudes and behaviors via communication will seldom succeed unless they take into account the power of place" [66 p116]. Building on this perspective, Community Voices is designed to emphasize a place-based mode of communication. However, the studies described here largely made use of online samples comprised of individuals located throughout the U.S., and thus provide a worst-case scenario for testing the effect of Community Voices without the power of place. To assess this deficit, Study 3 included a local as well as a national sample and used images and quote attributions from this local community to evaluate whether child messengers were more effective when they were recognizably from a viewer’s own community. We hypothesized that Community Voices would be more impactful if participants believed the content to come from their own community, particularly for child messengers (in comparison to unknown children).

The Oberlin College Institutional Review Board approved this research (IRB Protocol #SP14PCF/EJP/ERS-02). All participants recruited provided electronic informed consent prior to participating in the study. Data, metadata, syntax, and output files for all three studies are archived with Dryad, https://doi.org/10.5061/dryad.np5hqbzs4.

## Study 1

To address Question 1, whether exposure to CV shift norms, concern, and commitment to act, Study 1 compared those exposed to CV to a non-exposure control group. To address Questions 2, 3a, and 3b, it also compared several approaches to message framing and attribution. Specifically, it compared injunctive vs non-injunctive framing of messages and political vs. non-political messages and examined whether children might be more effective than adults at delivering these types of messages. The study used a 2 (messenger: child vs. adult) X 2 (politics: political vs. apolitical) X 2 (voicing: injunctive vs. non-injunctive) between-participants design, with an additional non-exposure control condition. This resulted in eight separate exposure conditions plus one control condition. We hypothesized that exposure to CV would increase norms, concern, and commitment to act (tested by the main effect comparing Community Voices exposure to no exposure). We hypothesized that children would be more effective messengers than adults (a main effect of messenger). We hypothesized that both an injunctive frame and a political frame would be more effective when the message was attributed to children (the 2-way interactions between messenger and politics and messenger and voicing). S1 Text presents power analyses indicating that our main effect and *a priori* simple effect analyses were sufficiently powered to detect an effect.

### Method

#### Participants

A total of 866 participants were recruited from the U.S. via Mechanical Turk (Mturk) and received $1.00 in exchange for their participation. The purpose of the study (as well as Studies 2 and 3) was described thus: “We are interested in getting feedback on one component of a display that will appear on public display screens in many communities.” The overall sample was 57% male and 75% white, with an average age of 35 years. The sample was 44% self-identified Democrats, 14% Republicans, and 42% Independents or other. 86% of the sample reported having completed at least some college.

#### Procedure

Participants were told, “In this study, you may or may not see a series of slides that show pictures and quotes. You will then be asked to answer questions about the slide show and your beliefs and concerns.” Each participant was randomly assigned to one of the eight experimental conditions discussed above or to the control condition. Those in the experimental conditions were told “This is a slide show containing 16 images paired with quotes, which will last 4 minutes. We ask that you watch the entire slide show for the purposes of this study. We also suggest that you watch it in full screen to be more able to view the pictures on the slides. After the slide show is finished you may proceed to the next part of the study.” They then watched one of 8 versions of a CV slideshow (one for each condition) that was embedded in the survey.

Each slideshow included 16 slides; each slide was shown for 15 seconds, in the form of a 4 min video presentation embedded via YouTube. A separate slideshow was created for each of the eight experimental conditions. The slideshows simulated the type of content shown on CV; each slide consisted of one image paired with one quote (e.g., Fig 1). For the purpose of this study, the content of the quotes was constructed by our research team, but the type of phrasing was designed to plausibly match content we have previously developed through interviews with community members. Slides used in the different conditions were as similar as possible to each other in general content; they differed with respect to the photographic image (child vs. adult messenger) and/or how messages were framed (political vs. non-political, injunctive vs. non-injunctive). All 16 slides had content consistent with the framing for that condition. See Table 1 for examples of the text content depicted on slides, S1 Images for the images used in Study 1 as well as Studies 2 and 3), and S2 Text for a complete list of all message content.

**Table 1:** Examples of messages in treatment groups for Studies 1 and 2^a^.

Study	Framing	Condition	Example message
1	Apolitical	Injunctive	You should use less energy to help fight climate change
1		Non-injunctive	I try to use less energy to help fight climate change
1	Political	Injunctive	Our government needs to do something about climate change
1		Non-injunctive	Our government can do something about climate change
2	Temporal orientation	Present	Keep remaking, keep reusing, to keep the world clean
2		Future	Keep remaking, keep reusing to keep the world clean in the future

^a^Adult vs. child was compared in all cases, and manipulated through the picture and quote attribution.

The child vs. adult condition was manipulated by associating text with a photo of either an adult or a child and through the attribution inserted below the quoted text; this attribution consisted of either a grade (3^rd^ - 6^th^) after the quotes accompanying all 16 images, or it consisted of only a name (implying adult). Ten of the slides in each slide show depicted images of people (either all adults or all children). The number of people, race, gender, and activities depicted in a photo associated with each quote were matched in slides that contained images of adults and children (e.g., Fig 1). The remaining 6 photographs included in each slide show were of natural environments, and were held constant across all conditions. The quotes were identical in adult and child conditions and designed such that they could plausibly be attributed to either age level.

The political vs. apolitical experimental condition was manipulated by the content of the quotes. Each political quote mentioned a political body, political person, or political action by using keywords or phrases in each quote such as: “take a stand”, “the city”, “organize”, “leaders”, “congress”, “president”, “make laws”, and “government”. Apolitical quotes did not contain these keywords and avoided language that inferred collective action (see Table 1, S2 Text). All quotes referred to the same topics.

The injunctive vs. non-injunctive condition was also manipulated by the content of the quotes. “Injunctive” is operationally defined in this experiment as a statement that implies necessity of the action. This was manipulated by including phrases such as “need to” and “should” (injunctive) versus “can”, “could”, or “want” (non-injunctive). Additionally, the non-injunctive statements referred to the person who said the quote by emphasizing use of the first-person singular pronoun “I”. Injunctive statements referred to actions the viewer could do by using a “you” pronoun construct (e.g., “you should” vs “I try”) (see Table 1, S2 Text).

After viewing the video, participants exposed to each of the eight conditions answered a number of questions designed to measure the slideshow’s impact (described below). The entire task of watching the slideshow and answering the survey questions took participants in Study 1 an average of 15 minutes. Those in the control condition did not view a CV slideshow; this group was simply asked to answer the same set of survey questions.

#### Measures of impact (dependent variables)

We employed nine distinct psychological measures described below to assess the impact of exposure to the eight experimental conditions, in comparison to the control condition and to each other. All instruction, items, operational definitions, and alphas are listed in S2 Text. We also collected demographic information and included a question that functioned as a manipulation check. Unless otherwise indicated, all measures below used a 5-point Likert scale, for example ranging from “1 = strongly disagree” to “5 = strongly agree”.

To measure how the CV slideshow affected participants’ mood, we developed a mood scale, which consisted of 9 items assessing an individual’s perceived levels of different emotions, such as “happy,” “sad,” “tense,” and “guilty.” Participants were asked to use a sliding scale, ranging from 0 (“I don’t feel this way at all”) to 100 (“I very much feel this way”). This was the only measure of impact that was assessed both before and after participants viewed the CV slideshow. All subsequent questions were only asked following exposure. Negatively worded items were reverse scored, and the nine items were averaged to create a single mood score. The scale proved reliable, alpha_T1_ = .84, alpha_T2_ = .81.

We had some concern that the injunctive messages could cause a negative reaction. To measure participants’ perception that the CV slideshow they experienced was “preachy” and manipulative, we asked respondents to indicate their level of agreement with three statements (e.g. “I felt the slide show was preaching at me”); alpha = .84. These items were averaged together.

Participants were asked to indicate the extent to which they were concerned about 14 environmental problems, such as “rising temperatures,” “deforestation,” and “water drought” (α = .94). We intentionally included environmental items that were not addressed in the CV slideshow as well as those that were so that we could assess the potential for spillover effect from one type of environmental concern to another; six of the items in the scale referenced environmental problems that were directly mentioned in the slideshow that they viewed (α = .90), and eight of the items were not mentioned (α = .89. Responses to the different problems were averaged to create two separate scores for problems mentioned and problems not mentioned in the slide show.

Participants also indicated the extent to which they were committed to 13 different pro-environmental actions, such as “bicycling/walking,” “conserving water,” and “shopping locally” (α = .91). Five of the items in the scale referenced actions that were directly mentioned in the text of CV slideshow (α = .78), and 7 of the items were actions that were not mentioned in the slideshow (α = .85). Again, this allowed for an assessment of spillover effects. Responses to the different commitments were averaged to create separate scores for commitments to actions mentioned and actions not mentioned in the slideshows presented. An important caveat is that, as a result of a coding error, we did not record data for one of the 13 actions.

Participants then indicated their general awareness and perception of children and adults being involved in pro-environmental action in the world. Four items (averaged together) addressed children (α = .77), and 4 items (averaged together) addressed adults (α = .59). For example, items included “[youth/other people] are taking action to protect the environment.” Although the reliability for the adult subscale was low, results did not change when we dropped the item that correlated least well. Further, reliabilities for this subscale were much higher in Studies 2 and 3; to be consistent across studies we used the scale as is.

#### Efficacy and responsibility

Six items assessed participants’ perception of efficacy and responsibility (we originally conceived of these as separate constructs, but exploratory factor analysis clearly yielded a single factor). For example, items included “I can do things to make the environment better,” “what people do now affects the environment in the future,” and “people have a responsibility to protect the environment for future generations”. All six items were averaged together to create a single scale, (α = .91).

A single item, “I think the environment will be better in the future" was used to measure optimism.

The Connectedness to Nature Scale-Revised (CNS-R) measures the degree to which an individual feels like an egalitarian member of the natural world [67]. Five items from the scale were used, such as “I think of the natural world as a community to which I belong” (α = .93). Responses to these items were averaged to create a single CNS-R score for each respondent. Connectedness to nature was not expected to be influenced by exposure to CV; it was included as a covariate because prior research indicates that it correlates strongly with pro-environmental thought and behavior.

In addition to the collection of measures, demographic information was collected on gender identity, age, ethnicity, geographic location, urban vs. rural, education, and political orientation (measured on a seven-point Likert scale from liberal to conservative).

#### Manipulation check

In addition to the psychological measures described above, we included three manipulation check questions. We asked respondents to indicate their agreement (on a 5-point scale, from strongly disagree to strongly agree) to the following statements: “The quotes I read came from adults” (child vs adult), and “The messages were overtly political” (political vs non-political, and “the messages suggested things I *should* do” (injunctive vs non-injunctive)”.

### Results

We used a casewise deletion approach (rather than listwise) to incomplete and missing data, thus cell sizes vary slightly between analyses.

#### Manipulation checks and preliminary analyses

We ran a series of independent sample t-tests to evaluate whether our manipulations (child vs adult, injunctive vs non-injunctive, political vs non-political) created the intended impression among participants. Participants who saw adult messengers (N = 356) were much more likely to say they saw adults than those who saw child messengers (N = 377), *p* < .001, *Cohen’s d = 1*.*02*. People who saw slides with injunctive messages were much more likely to say they saw slides that mentioned things they *should* do (N = 355) than those who didn’t (N = 378), *p* < .001, *Cohen’s d* = 1.09. People who saw political messages (N = 374) were much more likely to say the messages were overtly political than those who did not see political messages (N = 357), *p* < .001, *Cohen’s d* = 1.11.

We also tested whether the injunctive or political messages increased negative mood, or made people feel preached at, using two 2 (child vs adult) x 2 (political vs apolitical) x 2 (injunctive vs non-injunctive) x 2 (time 1 vs time 2) mixed model ANOVAs. We were particularly interested in whether this effect was weaker for child messengers. While all conditions saw a decrease in positive mood from Time 1 to Time 2 (*M*_change_ = 1.99, *SD* = 8.22, *F*(1, 430) = 33.32, *p* < .001, *partial eta squared* = .07), there were no significant differences in mood change between the conditions, *p’s* > .13. Contrary to our expectations, the slideshow was not experienced as less preachy in the child messenger condition (*p* = .70). In fact, there was a significant political by injunctive interaction (*F* (1, 726) = 23.09, *p* < .001, *partial eta squared* = .03) such that those who saw political injunctive messages gave the slide show the lowest ratings on preachiness. No other effects were significant.

#### Q1: Testing overall effects of community voices

The main dependent variables were subjected to a series of one-way ANCOVAs comparing those who did not view the CV slideshow (the control condition) to all who did view it. Because numerous studies (e.g. [68–72]) have shown attitudes about environmental issues vary by connectedness to nature and political orientation, we planned *a priori* to use these two background measures as covariates. Although both were measured after our manipulation, there were no significant differences between conditions on either variable. Inspection of correlation tables confirmed that CNS-R and political orientation correlated strongly and significantly with virtually all of our dependent variables. Including these background variables reduces error variance and increases statistical power [73]. We have also included all analyses without the covariates in S3 Text. One result went from significant to marginal (noted below), otherwise results are identical. We also tested whether participants who identified as liberals, independents, or conservatives responded differently to message frames, but did not find any significant effects. Throughout we report adjusted means and SEs, controlling for CNS and political orientation.

Consistent with our hypothesis, those exposed to CV slides exhibited significant differences from those in the control condition for many, but not all, of the dependent variables (see Table 2 for a summary of the results). Relative to the control condition, participants exposed to CV exhibited a higher level of environmental concern regarding issues that were directly mentioned in the slideshow and also for environmental issues that were not mentioned in the slideshow. Those exposed to CV content reported a higher sense of efficacy and responsibility for environmental protection. The exposed group also perceived environmental protection as more normative among both adults (marginally significant, *p* = .08) and children (significant, *p* < .001). However, though the means were in the predicted direction, those exposed to CV did not report significantly higher levels of commitment to action or optimism about the future.

**Table 2:** Adjusted means (controlling for CNS and political orientation), SEs, and F statistics comparing exposure to community voices vs no exposure.

Variable	Community Voices (N = 733) Mean (SE)	No Community Voices (N = 88) Mean (SE)	F	p	Partial eta squared
Concern, overall	3.60 (0.03)	3.31 (0.08)	13.19	< .001**	.02
Concern, mentioned in CV	3.80 (0.03)	3.44 (0.08)	17.99	< .001**	.02
Concern, not mentioned in CV	3.46 (0.03)	3.21 (0.08)	8.56	.001**	.01
Behavioral commitment, overall	5.85 (0.05)	5.67 (0.13)	1.69	.19	--
Behavioral commitment, mentioned in CV	5.80 (0.05)	5.60 (0.14)	1.91	.17	--
Behavioral commitment, not mentioned in CV	5.89 (0.05)	5.72 (0.15)	1.25	.27	--
Efficacy & responsibility^1^	4.23 (0.02)	4.13 (0.06)	5.50	.02**	.01
Optimism	2.98 (0.04)	2.93 (0.11)	0.20	.66	--
Perceived norms, children	3.44 (0.03)	3.20 (0.07)	11.90	.001**	.01
Perceived norms, adult	3.69 (0.02)	3.60 (0.06)	3.01	.08*	.004

^1^ When covariates are omitted, this result becomes marginal, p = .06.

** = significant at the .05 level

* = significant at the .10 level

We ran two 2 (CV exposure vs not) x 2 (mentioned vs not mentioned) mixed model ANCOVAs comparing concern for and commitment to behavioral change for issues that were mentioned versus not mentioned in the slide show. Both those exposed and not exposed to CV expressed more concern (*F*(1, 817) = 17.94, *p* < .001, *partial eta squared* = .02) for the issues mentioned in the slideshow, relative to those not mentioned. This suggests that the issues chosen for inclusion in the slideshow were inherently more compelling than those not included. The interactions were not significant.

#### Q2, Q3a, Q3b: Effects of messenger and message type

We ran a series of 2 (child vs adult) x 2 (political vs non-political) x 2 (injunctive vs non-injunctive) ANCOVAS (with connectedness to nature and political orientation as covariates) to test whether the messenger, political content of the messages, and injunctive tone of the messages influenced responses. When appropriate we included a within-subjects factor to test differences between content mentioned in the slideshow in comparison to content not mentioned. Because the potential for family wise error is high, we are not reporting marginal effects or interaction effects that are irrelevant to our hypotheses and did not replicate across at least two dependent variables. We also ran *a priori* simple comparisons evaluating child vs adult messenger, split by voice and framing. A summary of analyses without the covariates can be found in S3 Text. Without the covariates, an effect of messenger on the commitment variables emerged (marginal for commitment to those issues mentioned in the slideshow), with child spokespeople yielding higher levels of commitment. No other differences emerged.

**Fig 2:**
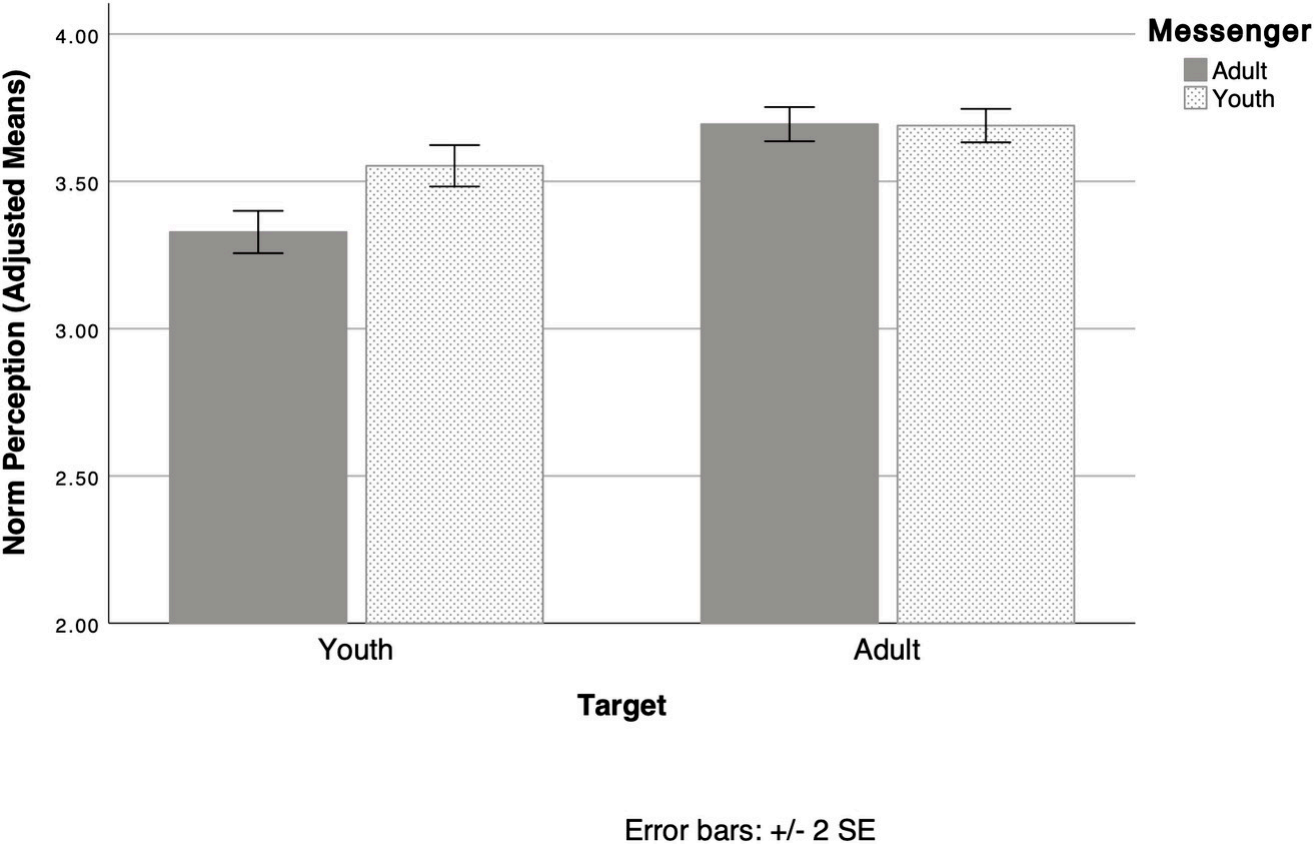
Interaction between messenger and norm perceptions for youth vs adults. Those exposed to child messengers saw youth action on environmental issues as significantly more normative than those exposed to adult messengers. (All means adjusted to control for CNS and political orientation. Error bars represent +/- 2 SE.).

As we reported above, participants were more concerned about issues mentioned in the slideshow than issues not mentioned. Contrary to Hypotheses 2, 3a, and 3b, there were very few significant effects of messenger or message frame, and those that emerged were not consistent with each other. We detected a messenger effect on norm perception (see Fig 2). We ran a 2 (child vs adult messenger) x 2 (political vs non-political) x 2 (injunctive vs non-injunctive) x 2 (youth norms vs adult norms) mixed model ANCOVA, controlling for CNS and political orientation, comparing participants’ perception of child and adult norms (i.e. perceptions that children and adults engaged in pro-environmental action). There was a significant main effect for adult vs child norm perception, *F (1*, *723)* = 28.18, *p* < .001, *partial eta squared* = .04. Regardless of which messages they were exposed to, participants perceived that adults (*M* = 3.69, *SE* = 0.02) were more active in environmental conservation efforts than youth (*M* = 3.44, *SE* = 0.03). There was also a main effect for the messenger condition, but it was qualified by a significant target (youth vs adult norms) by messenger interaction, *F* (1, 724) = 30.52, *p* < .001, *partial eta squared* = .04. Perception of adult norms did not change based on whether there was a child or adult messenger, (b0oth *Ms* = 3.69). However, perception of child norms was significantly higher in the child condition (*M* = 3.55, *SE* = 0.04) than in the adult condition (*M* = 3.33, *SE* = 0.04). In other words, exposure to adult messengers did not increase perceptions of adult norms; but exposure to child messengers did increase perception of child norms. None of the simple effects comparisons approached significance, which suggests that child messengers are not more effective at delivering injunctive or political messages.

### Discussion

Overall, Study 1 provides strong evidence that a brief exposure to Community Voices increases pro-environmental thought. Exposure increased concern about environmental issues that were not mentioned in the slideshow as well as those which were mentioned, which suggests a spillover effect. Exposure also increased the perceptual norm that others are engaged in environmental protection (significant for perceptions of children, marginal for adults). Exposure likewise increased a sense of efficacy and responsibility to act. Together, these findings suggest that, in general, CV is having its intended impact.

However, we found relatively few differences in the impact of child vs. adult messengers and alternative message framing. Overall, injunctive messages were not more or less effective than non-injunctive messages. Political messages were not more or less effective than non-political messages. In particular, contrary to our hypotheses, children were not more effective at delivering injunctive or political messages. Only one predicted significant effect of messenger emerged; Those who saw child messengers had higher estimates of children being involved in pro-environmental behavior than those who saw adult messengers.

## Study 2

Because Study 1 provided no evidence that the political or injunctive nature of messaging had an impact, we pursued another line of inquiry in Study 2. Today’s children will be more impacted by environmental degradation than today’s adults; thus, children may evoke more concern and perhaps be seen as having more of a stake in environmental protection if future impacts are highlighted (Q3c). Study 2 sought to replicate the finding from Study 1 that CV has an overall impact (Q1, tested by the main effect comparing Community Voices exposure to no exposure). In addition, it sought to test the hypothesis that highlighting future consequences would increase the impact of CV, particularly if the messengers were children (Q3c, tested by the main effect of tense and the messenger x tense interaction). Study 2 used a 2 (child vs. adult) X 2 (present vs. future) between-participants design, with an additional non-exposure control condition. The overall approach to this study was similar to Study 1; however, message content and certain survey questions differed as described below.

### Method

#### Participants

A total of 509 participants were recruited via Mechanical Turk; those who participated in Study 1 were ineligible to participate. Participants received $1.00 in exchange for participation. The sample was 53% male and 75% white, with an average age of 35 years. The sample was 44% self-identified Democrats, 16% Republicans, and 40% Independents or other; 84% of the sample reported having completed at least some college, and 41% reported having at least one child.

#### Procedure and materials

Each participant was randomly assigned to one of the four experimental conditions or to the control condition. In the four experimental conditions, the appropriate slideshow was embedded into the survey. Participants were told “This is a slide show containing 12 images paired with quotes, which will last 3 minutes (You will not be able to click next and continue until the video is finished). We ask that you watch the slide show until you are instructed to continue. Please watch in full screen.” After viewing the slideshow, participants answered questions similar to those used in Study 1. Those in the control condition were directed immediately to the survey questions. The entire task took an average of 14 minutes for those who viewed the slideshow.

As in Study 1, participants watched an embedded YouTube slideshow of content from CV. In this case, each slideshow consisted of 12 slides, presented for 18 seconds each. The child vs. adult condition was manipulated by the images shown in the slideshow. The quotes accompanying all 12 images (which were different from those in Study 1, see S2 Text) were attributed to a fictional person who was either issued a name and grade (3^rd^ - 6^th^) in the child condition or simply with a name (implying adult). Additionally, of the 12 images, 5 were images of natural environments while the remaining 7 were of either adults or children matched by number of people, race, gender, and activity in photo. All quotes were phrased so that they could be plausibly said by either an adult or a child.

The present vs. future condition was manipulated by the quotes on the slides. Quotes referenced either the present or the future with duplicate text appended to this stem. Quotes referenced the present via phrases such as “right now” and “current.” Quotes referenced the future via phrases such as “in the future,” “down the line,’” and “for years to come.” All slideshows contained the same 12 images, and the quotes were matched for theme and length across each condition (see Table 1 and S1 Text for examples and complete quotes).

#### Measures

The following measures from Study 1 were also used in Study 2: environmental concern, behavioral commitment, norm perception, efficacy and responsibility, optimism, and connectedness to nature (see S2 Text for full survey, operational definitions, and scale alphas). We added an additional demographic variable for participants that assessed number of children and age of children to assess whether parental status of participants might alter response. Our manipulation check questions for Study 2 asked how much participants agreed that they had seen messages about the future, and messages from adults. To determine whether child messengers elicited more empathy we also added three questions that asked participants to use a 5-point scale to rate the strength of their agreement with the statement that the slides they viewed caused them to feel compassion, concern or touched by the people depicted in the slideshows (α = .879).

### Results

As in Study 1, we used a casewise deletion approach (rather than listwise) to incomplete and missing data, thus cell sizes vary slightly between analyses.

#### Manipulation checks

We ran a series of independent sample t-tests to assess whether our manipulations (future vs present messages, child messengers vs adult messengers) created the intended impression among participants. Participants who saw adult messengers (N = 213) were much more likely to agree that they saw adults than those who saw child messengers (N = 213), *p* < .001, *Cohen’s d* = 1.02. People who saw slides about the future (N = 211) were much more likely to agree that they saw slides that talked about the future than those who saw slides about the present (N = 215), *p* < .001, *Cohen’s d* = 1.13.

#### Q1: Testing overall effects of *c*ommunity voices

The main dependent variables were subjected to a series of one-way ANCOVAs comparing those who did not view the CV slideshow to those who viewed it. All analyses controlled for CNS scores and political orientation, as both of these measures were highly correlated with our dependent variables (a summary of the analyses without covariates can be found in S4 Text). As in Study 1, and consistent with our hypothesis, all means were in the predicted direction. Most, but not all, of our effects replicated Study 1 (see Table 3 for a summary of the results.) Relative to the control condition, participants who saw the CV slideshow reported more concern about environmental issues (about both those mentioned and not mentioned in the slideshow); they reported higher levels of commitment to taking action on environmental issues (marginally for those mentioned, significantly for those not mentioned); they reported higher levels of optimism; and they perceived environmental protection as marginally more normative among both adults and children.

**Table 3:** Adjusted means (controlling for CNS and political orientation), SEs, and F statistics comparing exposure to community voices vs no exposure.

Variable	Community Voices (N = 421) Mean (SE)	No Community Voices (N = 82) Mean (SE)	F	p	Eta squared
Concern, overall	3.62 (0.03)	3.43 (0.07)	5.25	.02**^1^	.010
Concern, mentioned in CV	3.74 (0.04)	3.56 (0.08)	3.95	.05*	.01
Concern, not mentioned in CV	3.57 (0.03)	3.38 (0.08)	5.31	.02**^1^	.011
Commitment, overall	3.07 (0.03)	2.89 (0.08)	4.25	.04**^2^	.008
Commitment, mentioned in CV	2.80 (0.04)	2.65 (0.09)	2,59	.11	.005
Commitment, not mentioned in CV	3.19 (0.04)	3.00 (0.08)	3.92	.05**^2^	.008
Efficacy & responsibility	4.13 (0.03)	4.06 (0.06)	1.61	.22	--
Optimism	3.12 (0.05)	2.82 (0.12)	5.07	.03**	.010
Perceived norms, children	3.46 (0.04)	3.30 (0.09)	2.66	.10*	.005
Perceived norms, adult	3.96 (0.03)	3.84 (0.07)	2.35	.13	--

** = significant at the .05 level

* = significant at the .10 level.

^1^ Becomes marginal when covariates are omitted.

^2^ Becomes nonsignificant when covariates are omitted.

We ran two 2 (CV exposure) x 2 (mentioned vs not mentioned) mixed model ANCOVAs comparing concern for and commitment to behavioral change for issues that were mentioned and not mentioned in the slide show. As in Study 1, both those exposed and those not exposed to the CV slideshow expressed more concern for the issues mentioned in the slideshow. (*F*(1, 498) = 11.50, *p* = .001, *partial eta squared* = .02). They did not express more behavioral commitment for the mentioned issues, however. The interactions were again not significant.

#### Q2, Q3c: Effects of messenger and message type

We ran a series of 2 (messenger: child vs adult) x 2 (tense: present vs. future) ANCOVAS (with connectedness to nature and political orientation as covariates) to test whether the messenger and tense influenced responses to CV. We initially included participants’ parental status as a fixed factor; however parental status did not significantly interact with either messenger or tense and was thus dropped from our models. When appropriate we included a within-subjects factor to test differences between dependent variables that were related to each other. Specifically, we compared content mentioned by the slideshow to content not mentioned, and perception of child norms to adult norms. Because the potential for family wise error is high, we are not reporting marginal effects or interaction effects that are irrelevant to our hypotheses and did not replicate across at least two dependent variables.

Similar to Study 1, participants perceived pro-environmental behaviors as more normative among adults (*M* = 3.90, *SE* = 0.03) than among children (*M* = 3.38, *SE* = .05, *F*(1, 415) = 35.90, *p* < .001, *partial eta squared* = .080. Consistent with Study 1 there was also a significant target (adult vs child norms) by messenger interaction, *F (1*, *415)* = 12.19, *p* = .001, *partial eta squared* = .029. Post-hoc independent sample t-tests indicated that adult norm perception did not change based on whether there was a child or adult messenger, *t*(424) = 1.70, *p* = .09. However, as in Study 1, child norm perception was significantly higher in the Child condition than in the Adult condition, *t(424)* = 4.32, *p* < .001. Regardless of messenger condition, all participants held the same norm perceptions of adult others; however, participants who viewed children perceived that children were more active in environmental issues than people who viewed adults.

**Fig 3:**
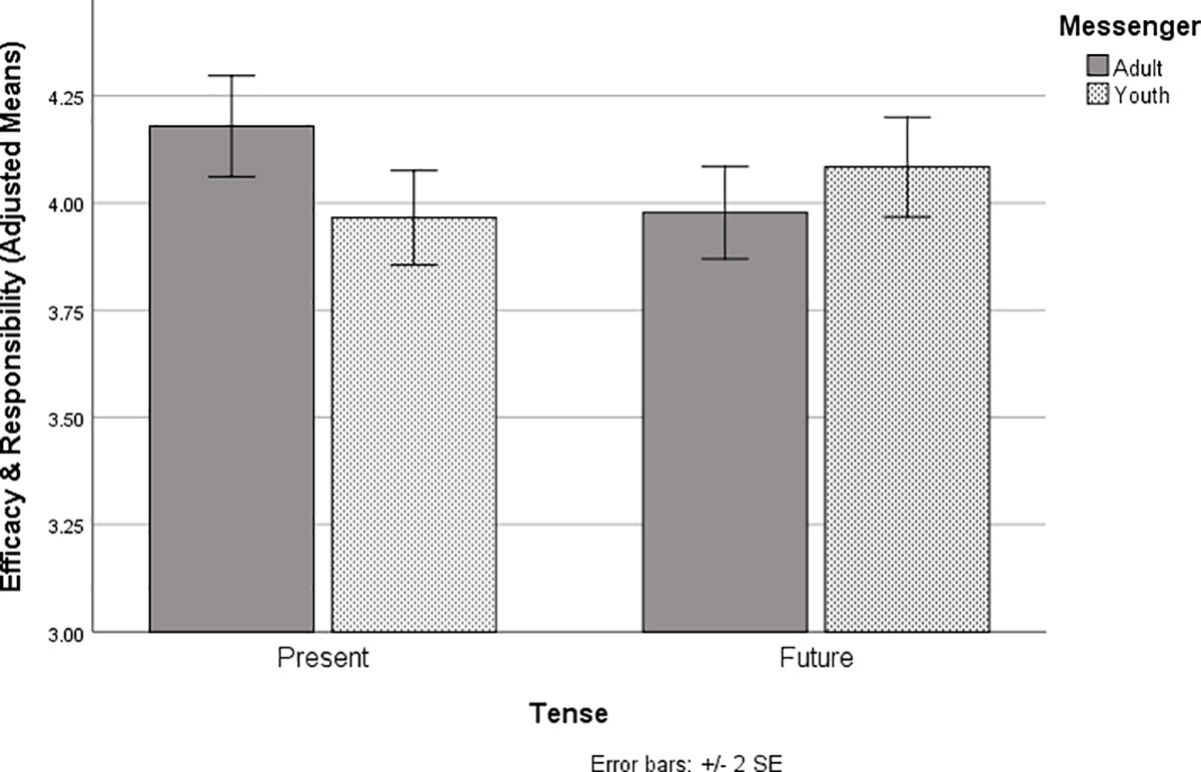
Interaction between messenger and tense of messages on feelings of responsibility and efficacy. Adult messengers using present tense increased efficacy and responsibility more than those using future tense; the pattern was reversed for child messengers.

Changes in norm perception notwithstanding, contrary to our hypotheses there were very few significant effects of messenger or tense at the level of the omnibus F. As predicted, child messengers (*M* = 3.64, *SE* = .06) elicited more empathy than adult messengers (*M* = 3.33, *SE* = .06), *F*(1, 415) = 12.39, *p* < .001, *partial eta squared* = .029. Additionally, there was a marginal messenger x tense interaction for efficacy and responsibility that was consistent with our hypothesis that child messengers would be more effective delivering messages in the future tense, *F*(1, 415) = 3.21, *p* = .07, *partial eta squared* = .01, see Fig 3. Compared to child messengers, adult messengers resulted in marginally higher levels of efficacy and responsibility when messages were in the present tense, (*M* = 4.22, *SE* = .05) than those in the child messenger condition (*M* = 4.08, *SE* = .05), *F* (1, 207) = 3.49, *p* = .06, *partial eta squared* = .02. In contrast, child messengers resulted in marginally higher levels of efficacy and responsibility in the future tense condition, (*M* = 4.19, *SE* = .05) than those in the adult messenger condition (*M* = 4.05, *SE* = .05), *F* (1, 207) = 3.70, *p* = .06, *partial eta squared* = .02.

We also followed up with *a priori* simple comparisons (controlling for CNS and political orientation) testing whether the future child condition was different from the future adult condition for the variables that did not yield a significant omnibus F. Consistent with our hypothesis, those in the child messenger condition had marginally higher levels of commitment (*M* = 3.18, *SE* = .071) than those in the adult messenger condition (*M* = 3.01, *SE* = .066), *F* (1, 206) = 2.90, *p* = .090, *partial eta squared* = .01. They also had significantly higher levels of optimism (*M* = 3.31, *SE* = .11) than those in the adult messenger condition *M* = 2.89, *SE* = .11), *F* (1, 205) = 7.18, *p* = .008, *partial eta squared* = .03.

It should also be noted that when the covariates were removed from the messenger x tense ANOVAs, several nonsignificant interaction effects became marginal or significant (See S4 Text for a summary of these results). These effects are all consistent with our hypothesis (Q3c) that child messengers might be more effective when talking about the future.

We had intended to test whether any beneficial effects of child messengers were mediated by higher levels of empathy for children. Child messengers marginally outperformed adult messengers on efficacy and responsibility in the future condition. Standard tests of mediation yielded marginal results. Child messengers also outperformed adult messengers on optimism. Mediation analyses suggest that this effect was partially mediated by empathy (Sobel test = 2.65, p < .01, see S4 Text). Thus there is weak evidence that increased empathy for child messengers partially mediated the increase in optimism and efficacy and responsibility observed in that condition.

### Discussion

Study 2 replicated many of the findings from Study 1. Once again, as hypothesized, brief exposure to CV increased concern about and willingness to take action on environmental issues not mentioned in the messaging; it marginally increased perceptions that others are engaged in environmental protection. In addition, Study 2 found that exposure to CV also increased commitment to taking action and optimism about solving environmental problems. Study 2 did not replicate the finding from Study 1 that exposure increased efficacy and responsibility, though the means were in the predicted direction. Together with Study 1, however, the results suggest that CV has a broad and reliable impact on pro-environmental thoughts and feelings.

Study 2 also found few beneficial effects of children as messengers (contrary to our hypotheses). While children did elicit more empathy, this did not seem to translate into increases in other dependent variables. The analyses in which covariates were omitted (see S4 Text) and *a priori* simple comparisons did provide weak evidence that child messengers are more effective than adults when talking about the future. Those who saw child messengers in the future condition reported marginally higher levels of efficacy and responsibility, marginally higher levels of commitment to act, and significantly higher levels of optimism. A retrospective power analysis (see S1 Text) suggests that our study did not have adequate power to detect significant 2-way interactions. These weak but suggestive findings deserve further study with a larger sample. Additionally, it is clear that child messengers do increase norm perception about children.

## Study 3

One key feature of CV that was not reflected in Studies 1 and 2 is its emphasis on community-specific content. In Study 3 we assessed whether messengers that participants believe come from their own community might be more effective than strangers (Q3d). We hypothesized that local messengers would be more effective than non-local messengers (tested by the main effect of locality). We also hypothesized that child messengers would be more effective than adults when participants believed they came from their own community (tested by the locality x messenger interaction).

In Study 3 we collected data from a population local to the CV display. These viewers knew that the content was generated in their own community and were highly likely to recognize the people and places depicted. This sample was contrasted to a sample drawn from Mturk and exposed to the same content. Once again, half of our participants saw messages delivered by child messengers, and half saw messages delivered by adults. Because we failed to find message content effects in Studies 1 and 2, and because we wished to maximize our statistical power to detect the hypothesized interaction between messenger and sample, we did not systematically vary message content of the CV slides in any way besides the messenger. Because we were not constrained by a need to systematically vary message content, the quotes used in Study 3 were actual quotes from community members (their real names were not used). Further, because Studies 1 and 2 established the overall effect of CV on a variety of variables, and because our local population was limited in size, we omitted the no-exposure control condition.

### Method

#### Participants

A non-local sample of 292 participants was recruited from Mturk; those who participated in Studies 1 and 2 were ineligible to participate. Participants received $1.50 in exchange for their participation. The sample was 53% male and 76% white, with an average age of 34 years. The sample was 46% self-identified Democrats, 17% Republicans, and 32% Independents or other; 87% of the sample reported having completed at least some college.

In addition, a local sample of 104 participants was recruited in the City of Oberlin Ohio (population 8,300). Local non-profit organizations were asked to forward our survey link to their email lists. As an incentive for both the organizations and the participants, upon completion of the task, a $5 donation was made to a local non-profit organization of the participant’s choosing. The sample was 38% male and 89% white, with an average age of 60 years. The sample was 68% self-identified Democrats, 2% Republicans, and 26% Independents or other; 98% of the sample reported having completed at least some college. It should be noted that our local sample differed from the Mturk sample in several significant ways: it was older, more female, more educated, more White, and more liberal.

#### Procedure and materials

Procedures followed were similar to the prior two studies. Each participant was randomly assigned to one of two experimental conditions (messenger: adult vs. child). The appropriate slideshow was embedded into the survey. Participants were told “This is a slide show containing 13 images paired with quotes, which will last about 3 minutes (You will not be able to click next and continue until the video is finished). We ask that you watch the slide show until you are instructed to continue.” Each slide was presented for 15 seconds. As in prior studies, the child vs. adult conditions were identical except for the age of the people in the images. As in the previous studies, five of the 13 images were of the natural environment, in this case in and around Oberlin OH, while the remaining 8 were of either adults or children from this community. Slides of people in the adult and child conditions were matched to each other by quote and by number of people, race, gender and activity depicted in the photo, as in Fig 1. Quotes were chosen so that they could be plausibly said by either an adult or a child.

The images in the slides consisted of people and places in the local community and were thus potentially recognizable by the local sample but not by the non-local sample. In total, there were four experimental conditions: adult vs. child local and adult vs. child non-local (Mturk).

After viewing the slideshow, participants answered a series of survey questions. The entire task took the Oberlin sample an average of 23 minutes, and the Mturk sample an average of 13 minutes.

#### Measures

The measures in Experiment 3 were identical to those used in Experiment 2: Environmental concern, behavioral commitment, norm perception, efficacy and responsibility, optimism, empathy, and connectedness to nature. Demographic information collected was also as in Study 2. Manipulation check questions were used to determine how much participants agreed that they had seen children or adults in the slides and recognized people and places depicted in the photographs. Operational definitions of each item and scale alphas are available in S2 Text. Statistical output of all analyses, with and without covariates, is in S5 Text.

### Results

As noted above, we used a casewise deletion approach (rather than listwise) to incomplete and missing data, thus cell sizes vary slightly between analyses.

#### Manipulation checks

Independent sample t-tests were used to check that the local audience recognized the people and places in the slides. Both tests were significant: the local audience recognized significantly more people (*M* = 1.30, *SD* = 1.67) and places (*M* = 3.16, *SD* = 1.39) in the slideshow than the nonlocal audience recognized people (*M* = 0.10, *SD* = 0.44) and places (*M* = 0.28, *SD* = 0. 69), *Cohen’s d* = .94 and .93 respectively, *p*’s < .001.

Further, participants in the adult condition (N = 208, *M* = 4.05, *SD* = .99) more strongly expressed that the messages in the slideshow came from adults than participants in the child condition (N = 185, *M* = 2.90, *SD* = 1.32), *t(*391) = 9.85, *p* < .001, *Cohen’s d* = 1.16.

#### Q2, Q3d: Effects of message type and audience type

We ran a series of 2 (messenger: adult vs child) x 2 (audience: local vs. nonlocal) ANCOVAs (with connectedness to nature and political orientation as covariates) to test whether the messenger and audience influenced responses to CV. We also included age, gender, education level, and ethnicity (White vs non-White) to control for the large demographic differences between our samples. Because the messages were actual quotes, they did not map on cleanly to the items we used in Studies 1 and 2 to measure concern and commitment, so we did not make this distinction in Study 3. We did run a mixed model ANOVA comparing perception of child norms and adult norms to each other.

Since we did not include a non-exposure control in Study 3, we were not able to assess the effects of slideshow exposure relative to no exposure. As in Studies 1 and 2, participants indicated that adults were more likely to exhibit environmental protective behaviors (*M* = 3.95, *SE* = 0.04) than children (*M* = 3.47, *SE* = 0.05, *F*(1, 368) = 10.83, *p* = .001), *partial eta squared* = .03. Unlike Studies 1 and 2, there was not a significant target (adult vs child) by messenger interaction on norm perception, *F(1*, *368)* = 2.15, *p* = 0.14. In other words, seeing child messengers in this study did not increase norm perception of children in either sample.

Controlling for demographic variables, the local audience expressed significantly more concern (M = 4.02, SE = 0.09) than the nonlocal audience (M = 3.77, SE = 0.04), F(1, 372) = 5.83, p = .02. The local audience also expressed marginally more commitment (M = 3.67, SE = 0.09) than the nonlocal audience (M = 3.50, SE = 0.04), F(1, 372) = 2.65, p = .10. There were no other significant main or interaction effects of the messenger being a child vs an adult. When covariates were omitted, a number of significant differences between the local and nonlocal sample emerged (see S5 Text). However, given the differences between the two samples, we suspect these stem from these demographic differences, rather than a differential impact of CV on the local audience.

In short, there was virtually no support for the hypothesis that CV led to more environmental concern and commitment among a local audience (relative to an MTurk audience (Q3d), and no evidence that children were more effective messengers.

### Discussion

Study 3 tested whether a local audience, with a personal connection to the people and places depicted, made child messengers more effective than adults. Once we controlled for demographic differences between our two samples, there were almost no significant effects due to messenger, audience, or the interaction between the two. As S1 Text discusses, our study was adequately powered to detect a main effect of source or audience. The fact that we found no impact of child vs. adult messengers provided further evidence that children are not generally more effective as messengers than adults, even when those children (and adults) come from one’s own community.

## General discussion and conclusions

Taken together, these studies suggest that CV does, indeed, have desired psychological impacts. While there were subtle inconsistencies in findings between Studies 1 and 2 in terms of which dependent variables reached the level of statistical significance, the means in both studies were in the predicted direction and paint the same picture: those exposed to pro-environmental messaging in the form of CV slides that combine images and short quotes expressed more concern, commitment to taking action, more efficacy and responsibility, and more optimism than those who did not. Those exposed also saw environmental thought and behavior as more normative among both children and adults than control groups.

An additional important finding relates to spillover effects. As one might expect, we generally observed a stronger increase in concern for and intention to take action on environmental issues that were directly mentioned in the messages delivered. However, relative to the control, those exposed to the messages also exhibited a significantly higher level of concern and commitment to take action on environmental issues that were not explicitly addressed in the messages. Prior studies have shown mixed results, with some demonstrating limited spillover of interventions [e.g., 74, 75] and others demonstrating spillover [e.g. 76; see 77 for a review].

Contrary to our expectations, we did not find any main effects of message framing (injunctive vs non-injunctive, political vs apolitical, past vs present), despite the fact that all main effect analyses were adequately powered (*β* = .78 - .99). Strategic framing is known to help overcome ideological barriers to action in some circumstances [54, 78]. Our failure to find such effects may be because the particular frames we choose to evaluate were ineffective. It is also possible that there is no “one frame fits all” approach, and that different audiences respond to different framing [e.g. 79]. Other kinds of message frames–and the impact of different frames on subpopulations—should be evaluated in future research.

An important goal of this study was to assess whether children might be more effective messengers than adults, and if so, the conditions under which this is true. As a general conclusion, we found that, at least for the particular messaging used in this study, children did not prove to be more effective messengers than adults. There were two small exceptions to this general finding. First, in Study 2 those who received messages worded in the future tense delivered by child messengers reported marginally higher levels of efficacy and responsibility, marginally higher commitment to take action, and significantly higher optimism than those in the present tense condition. However, we did not find this effect on the other dependent variables. Second, Studies 1 and 2 (but not Study 3) found that exposure to child messengers increases normative perceptions of children’s engagement on environmental issues. However, this perception did not seem to translate into greater concern or commitment to taking action. Our post hoc power analyses (see S1 Text) indicated that we had adequate statistical power to detect interaction effects in Study 1 with a planned comparison. However, we did not have adequate power in Studies 2 and 3 to test interaction effects. Study 2 yielded 1 significant and 2 marginal planned comparison, as well as non-significant patterns of means that trended in support of our hypothesis that children would be more effective spokespeople when talking about the future; a better-powered test of the impact of future tense used by child messengers is thus warranted. In Study 3 the local audience had significantly higher concern, and marginally higher commitment than the nonlocal one. However, the large number of demographic differences between the two samples, and the lack of a control group, make it impossible to conclude anything definitively. The pattern of means shows not the tiniest hint of support for the idea that children are more effective messengers than adults. In sum, we did not find consistent or broad evidence for the effectiveness of child messengers on environmental messages displayed using CV.

It must be acknowledged that all of the data from Studies 1 and 2, and much of the data in Study 3, came from MTurk. Multiple studies (e.g., [80, 81]) have demonstrated that with basic safeguards in place MTurk yields high quality data from populations that are more diverse than college samples and other samples of convenience. However, they are not representative of the general population [82]. Further, there is concern that MTurk samples are more likely to be inattentive, and less likely to be naïve, than other sources of data [83]. While we cannot be certain these issues did not affect our data, we did include safeguards (manipulation checks, exclusion of previous workers in subsequent studies) to minimize the impact of these issues. Future research will ideally replicate these effects with different sample populations.

On a related note, one important question that was not sufficiently addressed by these studies is how much difference the local context makes on the effectiveness of CV. In its intended use as an approach to communication, CV was designed to extract messages and images from a local community, select those that meet the pro-environmental communications criteria discussed earlier, and then displays these messages to the same local community. This approach is explicitly designed to maximize the impact of social norms. The effectiveness of this approach is contingent on the degree to which the audience feels a connection to and kinship with the messengers and community represented. Studies 1 and 2 therefore provide something of a "worst-case scenario" for this: the participants had no local connection to the people or places in the slide. It is notable, therefore, that exposure to CV messaging still resulted in significant increases in pro-environmental thought and concern. Based on previous research cited earlier, we hypothesize that the effects would be even stronger among people who had a connection to the people and places depicted. However, once we controlled for the substantial differences in demographic characteristics of the local and nonlocal samples, we found only limited and flawed evidence that Community Voices was more impactful for the local audience (it significantly increased concern, and marginally increased commitment). Given the correlational nature of these results, we believe this hypothesis warrants further exploration.

These studies also do not directly demonstrate that CV displays will be effective in their intended field setting (displayed on digital signage installed in public locations within a community). Digital signage is increasingly ubiquitous in public locations such as schools, college campuses, banks, stores, and other locations [84, 85]. Unlike forms of media that individuals actively seek out (printed media, social media and other online media etc.), digital signage has the advantage of being “in your space, in your face”; most of us are at least passively exposed in multiple locations on a fairly continuous basis.

The participants in our studies were asked to seat themselves in front of a computer screen at a time and in a place where they were, presumably, free of distraction. They were exposed to CV for several continuous minutes without interruption. Furthermore, they were provided with a small incentive to participate. The psychological impact was then assessed immediately after this exposure. A real-world situation is quite different: people may only briefly focus on content on digital signs in public settings. Their attention may be subconscious rather than conscious. On the other hand, they are also likely to experience this content repeatedly and over a much longer period of time. In other words, there are reasons to conclude that exposure in the intended real-world context may be both more or less effective.

An extensive literature on priming (see [86, 87] for reviews) leads us to predict that CV in a field setting actually *will* have detectable long-term effects, despite the lack of conscious attention observers may pay to it. A field test in Oberlin OH, in which local residents were surveyed before and after digital signage with CV content was installed, support this contention. The results of this study will be reported in a subsequent publication.

## Supporting information

S1 Text Power analyses for Studies 1, 2 and 3. (DOCX)

S2 Text Survey items, operational definitions, and scale reliabilities for Studies 1, 2 and 3. (DOCX)

S3 Text Summaries of Study 1 analyses, both with and without covariates. (DOCX)

S4 Text Summaries Study 2 analyses, both with and without covariates. (DOCX)

S5 Text Summaries Study 3 analyses, both with and without covariates. (DOCX)

S1 Images Stimulus images used in Studies 1, 2 and 3. (ZIP)
