# Supplementary material for: Novel approach to delivering pro-environmental messages significantly shifts norms and motivation, but children are not more effective spokespeople than adults
Source: PLoS One. 2021 Sep 8;16(9):e0255457. doi: 10.1371/journal.pone.0255457 (PMC8425541; doi:10.1371/journal.pone.0255457)
Supplement: S1 Text — (DOCX) [file pone.0255457.s001.docx]

**S1: Power analysis**

Because we found so many null effects, we conducted power analyses to evaluate whether the lack of significant results arose from a lack of statistical power. To estimate likely effect sizes for our power analyses, we examined the literature on attitude change, particularly messenger effects and framing effects [e.g. 1,2,3 ,4]. Unfortunately, none of the studies we examined provided good matches to the exact conditions of our study; thus, all of our effect size assumptions are necessarily approximations. Further, we found great heterogeneity in the observed effect sizes, with Cohen’s d’s ranging from .03 up to 1, depending on the manipulation and comparison condition. Rather than cherry-pick effect sizes from particular papers (for example the classic Petty et al. [1] paper yielded fairly large messenger effect sizes -- .67 in the weak argument condition, .56 in the strong argument condition), we chose to use the more conservative estimate of d = .33, based on Albarracin & Shavitt’s [5] review of research on attitude change, for all our power analyses.

None of our cell sizes were exactly equal; this was especially true when comparing those exposed to Community Voices to those in the control condition. To adjust for this, we computed harmonic means to determine n (see Howell, 2017), as the standard error of means (and thus power) is proportional to 1/n (not n).

Estimating power for comparisons between two groups is relatively straightforward; estimating power for interactions requires a different approach. As Laken & Caldwell [6] explain, the statistical power of an interaction depends on the exact pattern of means. Crossover interactions (in which the relationship between two levels of a variable flips, depending on the level of the other variable) have higher statistical power than interactions that do not have a crossover pattern (ordinal interactions). Our hypotheses all predicted ordinal interactions (ie., that children would be more effective than adults in delivering injunctive, political, and future messages; we did not predict that adults would be *less* effective).

Power analyses were conducted using Laken & Caldwell’s [6] Superpower power analysis package in R. The syntax appears at the end of this supplement. Table S1.1 presents the results.

**Table S1.1.** **Results of power analysis using Superpower package in R.** All effect sizes are assumed to be d = .33. N_h_ = harmonic mean. Simple effects power analyses compare adult messengers to child messengers in the injunctive, political, future tense, and local conditions, respectively.

| **Comparison** | **Study** | **N_h_** | **Power, Ominbus F** | **Power, Simple Effect** |
| --- | --- | --- | --- | --- |
| **Main Effects** |  |  |  |  |
| Control vs CV | Study 1 | 733 | 0.84 |  |
|  | Study 2 | 420 | 0.78 |  |
| Adult vs Child Messenger | Study 1 | 366 | 0.99 |  |
|  | Study 2 | 213 | 0.93 |  |
|  | Study 3 | 216 | 0.93 |  |
| Voice: Injunctive vs Noninjunctive | Study 1 | 366 | 0.99 |  |
| Framing: Political vs Nonpolitical | Study 1 | 366 | 0.99 |  |
| Tense: Future vs Present | Study 2 | 213 | 0.93 |  |
| Audience: Local vs Nonlocal | Study 3 | 190 | 0.9 |  |
| **Interactions** |  |  |  |  |
| Messenger x Voice | Study 1 | 193 | 0.64 | 0.9 |
| Messenger x Framing | Study 1 | 193 | 0.64 | 0.9 |
| Messenger x Tense | Study 2 | 107 | 0.41 | 0.68 |
| Messenger x Audience | Study 3 | 98 | 0.38 | 0.64 |

The vast majority of our statistical tests had between adequate and excellent power. The tests of interaction effects were less well-powered (though the tests of simple effects in Study 1 were adequately powered). The interaction effects in Study 2 (messenger x tense) and Study 3 (messenger x audience) were not adequately powered, either at the level of the omnibus F or the simple comparison. We note this as a limitation in the main paper.

**R Syntax**

**Main Effects**

**Study 1, control vs CV**

library(Superpower)
design <- ANOVA_design(
 design = "2b",
 n = 157,
 mu = c(.333, 0),
 sd = 1)

power_oneway_between(design, alpha_level = 0.05)$Cohen_f

power_oneway_between(design, alpha_level = 0.05)$power

plot_power(design, alpha_level = 0.05, min_n = 45, max_n = 400)$plot_ANOVA

Study 2, control vs CV

library(Superpower)
design <- ANOVA_design(
 design = "2b",
 n = 137,
 mu = c(.333, 0),
 sd = 1)

power_oneway_between(design, alpha_level = 0.05)$Cohen_f

power_oneway_between(design, alpha_level = 0.05)$power

plot_power(design, alpha_level = 0.05, min_n = 45, max_n = 400)$plot_ANOVA

**Study 1, child vs adult, injunctive vs noninjunctive, political vs nonpolitical**

library(Superpower)
design <- ANOVA_design(
 design = "2b",
 n = 366,
 mu = c(.333, 0),
 sd = 1)

power_oneway_between(design, alpha_level = 0.05)$Cohen_f

power_oneway_between(design, alpha_level = 0.05)$power

plot_power(design, alpha_level = 0.05, min_n = 45, max_n = 400)$plot_ANOVA

**Study 2, child vs adult**

library(Superpower)
design <- ANOVA_design(
 design = "2b",
 n = 212,
 mu = c(.333, 0),
 sd = 1)

power_oneway_between(design, alpha_level = 0.05)$Cohen_f

power_oneway_between(design, alpha_level = 0.05)$power

plot_power(design, alpha_level = 0.05, min_n = 45, max_n = 400)$plot_ANOVA

**Study 2, present vs future**

design <- ANOVA_design(
 design = "2b",
 n = 213,
 mu = c(.333, 0),
 sd = 1)

power_oneway_between(design, alpha_level = 0.05)$Cohen_f

power_oneway_between(design, alpha_level = 0.05)$power

**Study 3, child vs adult**

library(Superpower)
design <- ANOVA_design(
 design = "2b",
 n = 216,
 mu = c(.333, 0),
 sd = 1)

power_oneway_between(design, alpha_level = 0.05)$Cohen_f

power_oneway_between(design, alpha_level = 0.05)$power

plot_power(design, alpha_level = 0.05, min_n = 45, max_n = 400)$plot_ANOVA

**Study 3, local vs nonlocal**

design <- ANOVA_design(
 design = "2b",
 n = 190,
 mu = c(.333, 0),
 sd = 1)

power_oneway_between(design, alpha_level = 0.05)$Cohen_f

power_oneway_between(design, alpha_level = 0.05)$power

**Interactions**

**Study 1 Interaction (ordinal)**

design <- **ANOVA_design**(
  design = "2b*2b",
  n = 193,
  mu = **c**(.333, 0, 0, 0),
  sd = 1)

ANOVA_exact(design, alpha_level = 0.05)

**Study 2 Interaction (ordinal)**

design <- **ANOVA_design**(
  design = "2b*2b",
  n = 107,
  mu = **c**(.333, 0, 0, 0),
  sd = 1)

ANOVA_exact(design, alpha_level = 0.05)

**Study 3 Interaction (ordinal)**

design <- **ANOVA_design**(
  design = "2b*2b",
  n = 98,
  mu = **c**(.333, 0, 0, 0),
  sd = 1)

ANOVA_exact(design, alpha_level = 0.05)

**References**

1. Petty RE, Cacioppo JT, Schumann D. Central and Peripheral Routes to Advertising Effectiveness: The Moderating Role of Involvement. *J Consum Res*. 1983;10(2):135-146.

2. Pena‐Marin J, Wu R. Disconfirming Expectations: Incorrect Imprecise (vs. Precise) Estimates Increase Source Trustworthiness and Consumer Loyalty. *J Consum Psychol*. 2019;29(4):623-641. doi:https://doi.org/10.1002/jcpy.1117

3. Nisbet EC, Hart PS, Myers T, Ellithorpe M. Attitude Change in Competitive Framing Environments? Open/ClosedMindedness, Framing Effects, and Climate Change. *J Commun*. Published online 2013:20.

4. Priester JR, Petty RE. The Influence of Spokesperson Trustworthiness on Message Elaboration, Attitude Strength, and Advertising Effectiveness. *J Consum Psychol*. 2003;13(4):408-421. doi:https://doi.org/10.1207/S15327663JCP1304_08

5. Albarracin D, Shavitt S. Attitudes and Attitude Change. *Annu Rev Psychol*. 2018;69(1):299-327. doi:10.1146/annurev-psych-122216-011911

6. Lakens D, Caldwell AR. *Simulation-Based Power-Analysis for Factorial ANOVA Designs*. PsyArXiv; 2019. doi:10.31234/osf.io/baxsf
