# Supplementary material for: Novel approach to delivering pro-environmental messages significantly shifts norms and motivation, but children are not more effective spokespeople than adults
Source: PLoS One. 2021 Sep 8;16(9):e0255457. doi: 10.1371/journal.pone.0255457 (PMC8425541; doi:10.1371/journal.pone.0255457)
Supplement: S2 Text — (DOCX) [file pone.0255457.s002.docx]

S2 Text. Messaging, survey items, operational definitions, and scale reliabilities for Studies 1, 2, and 3

Below we provide operational definitions and survey items for all variables reported in Studies 1, 2, and 3. Variable names used in the data files appear in brackets after the descriptive name. Data and metadata files for all three studies are archived with Dryad, <https://doi.org/10.5061/dryad.np5hqbzs4>.

**Operational Definitions for Community Voices Study 1**

**Conditions**

**Community Voices vs No Community Voices [prime]:** Roughly 1/9 of all participants were randomly assigned to a no-exposure control condition. The rest watched one of eight versions of the Community Voices slideshow (described below). See Table S1 for text of messages.

**Adult vs. Child [Child]**: This condition was manipulated by the images shown in the slide shows. Each slide show contains 16 images. All images were attributed to a fictional person who was either issued a grade (3^rd^-6^th^) in order to infer that they were a child or they were left blank with only a name shown (inferring adult). Additionally, of the 16 images, 6 were images of natural environments while the remaining 10 were of either adults or children matched along number of people, race, gender, activity in photo, and quality of photo. All quotes were controlled that they could be plausibly said by either an adult or a 5^th^ grader.

**Injunctive vs. Noninjunctive [Injunc]:** Injunctive for our purposes refers to the intentionality and necessity of the action in the quote. This was manipulated by changing phrases such as ‘need to’ and ‘should’ (Injunctive) to ‘can’, ‘could’, ‘want’ (Noninjunctive). Additionally, the noninjunctive statements referred to the person who said the quote by using ‘I’ and the injunctive statements referred to actions the reader could do by using ‘you’. AKA ‘Our leaders should’ vs ‘Our leaders can’. ‘You should’ vs ‘I can’.

**Political vs. Apolitical [Poli]**: Political for our purposes refers to the content of the quotes. Each political quote aims at a political body, political person, or political action. This was operationalized by using keywords in each quote that relate to the above-mentioned groups. Apolitical quotes did not contain these keywords and avoided language that inferred collective action. Keywords included: take a stand, the city, organize, leaders, congress, president, make laws, government.

**Table S1.** Messages appearing on Community Voices slides, by condition. Each message was paired with a picture of an adult and a child.

| INJUNCTIVE NONPOLITICAL | |
| --- | --- |
| Ashley S. | You should use less energy to help fight climate change |
| Madeline L. | You need to think about how the planet will be for kids in the future |
| Annie B. | You should do something to protect the forests |
| Justin B. | You should make changes in your life that help us waste less |
| Elizabeth S. | I think businesses need to be polluting less |
| Ashley W. | You should garden in your yard so you can have fresh food |
| George H. | You have to do more to stop hurting the environment |
| Anna C. | You should work to help our community and environment |
| Jack H. | People who use less water should be rewarded |
| Josh K. | You should care more about your electricity use |
| Laura H. | You've got to work towards having cleaner air |
| Jeremy C. | You should ride your bike more often |
| Nicole J. | You need to make sure not to pollute the water |
| Lori G. | You should help protect endangered animals |
| Phil M. | You should be using more renewable energy |
| Bobby M. | Our land is for everyone: humans, plants, and animals. You should be sharing it |
| NONINJUNTIVE NONPOLITICAL | |
| Ashley S. | I try to use less energy to help fight climate change |
| Madeline L. | I think about how the planet will be for kids in the future |
| Annie B. | I can do something to protect the forests |
| Justin B. | I can make changes in my life that help reduce waste |
| Elizabeth S. | I think businesses pollute too much |
| Ashley W. | I love gardening in my yard so I can have fresh food |
| George H. | I can do more to stop hurting the environment |
| Anna C. | I can work to help our community and environment |
| Jack H. | It would be nice to reward people who use less water |
| Josh K. | I care about how much electricity I am using |
| Laura H. | I can do my part to make the air cleaner |
| Jeremy C. | I ride my bike all the time |
| Nicole J. | I make sure not to pollute the water |
| Lori G. | I want to help protect endangered animals |
| Phil M. | I want to use more renewable energy |
| Bobby M. | Our land is for everyone: humans, plants, and animals |
| INJUNCTIVE POLITICAL | |
| Ashley S. | Our government needs to do something about climate change |
| Madeline L. | When we make laws, we need to think about how the planet will be for kids in the future |
| Annie B. | We should make sure that the government protect the forests |
| Justin B. | We need to take a stand on how much we waste in this city |
| Elizabeth S. | We need rules that punish businesses that pollute |
| Ashley W. | This city should start more community gardens so we have fresh local food for everybody |
| George H. | We need to do things to make our government stop hurting the environment |
| Anna C. | Our President should help our community and environment |
| Jack H. | Our government should reward people that use less water |
| Josh K. | Our leaders need to care more about using less electricity |
| Laura H. | Congress needs to make sure we have cleaner air |
| Jeremy C. | The government should make more bike paths |
| Nicole J. | We should make it against the law to pollute water |
| Lori G. | We should organize to protect endangered animals |
| Phil M. | Our leaders need to make sure we are using renewable energy |
| Bobby M. | Our land is for everyone: humans, plants, and animals. And our government should make sure we're sharing it |
| NONINJUNCTIVE POLITICAL | |
| Ashley S. | Our government can do something about climate change |
| Madeline L. | The laws we make today affect the planet for kids in the future |
| Annie B. | The government can do something to protect the forests |
| Justin B. | We can take a stand on how much we waste in this city |
| Elizabeth S. | We can make rules that punish businesses that pollute |
| Ashley W. | The city can start more community gardens to give fresh local food to everybody |
| George H. | I thInk our government can do more to stop hurting the environment |
| Anna C. | Our President can help our community and environment |
| Jack H. | Our government can reward people that use less water |
| Josh K. | Our leaders could care more about using less electricity |
| Laura H. | Congress can do something to help us have cleaner air |
| Jeremy C. | Our government can make more bike paths |
| Nicole J. | We can pass laws to stop water pollution |
| Lori G. | We can organize to protect endangered animals |
| Phil M. | Our leaders can help us use more renewable energy |
| Bobby M. | Our land is for everyone: humans, plants, and animals. And our government can help us share it |

**Covariates**

**Connectedness to Nature [CNS].** In response to the prompt “Rate the degree to which you agree with the following statements” participants responded on a 5-point scale (1 = Strongly disagree, 5 = Strongly agree) to 5 items: I often feel a strong connection to nature, I think of nature as a family I belong to, I see myself as a part of the greater circle of life, I feel that all living things in this world are connected and I am a part of that, like the trees in the forest I feel I belong to nature. The items were averaged together, alpha: .93.

**Political Orientation [PoliScale].** Participants responded to the item “Use the scale below to indicate the extent to which you view yourself as politically liberal or conservative,” with 1 = Liberal, and 7 = Conservative.

**Manipulation Checks**

To evaluate whether participants paid sufficient attention to the content of the slideshow, and to evaluate whether the messages were offputting, we asked six questions (described below) with the prompt “Please indicate your agreement to the following statements below” on a 5-point scale (1 = Strongly disagree, 5 = Strongly agree).

**Political vs apolitical** **[PolManCheck]** was evaluated with the item “The messages were overtly political”.

**Child vs adult spokesperson** **[MessManCheck]** was evaluated with the item “The quotes I read came from adults.”

**Injunctive vs non-injunctive** **[InjuncManCheck]** was evaluated with the item “The messages suggested things I *should* do”.

**Preachiness [Preach]** was evaluated with 3 items (which were averaged together): I felt the slide show was preaching at me, I felt the slide show was manipulative, the slide show was designed to make me feel guilty. Alpha = .84.

**Dependent Variables**

**Mood [Mood1, Mood2].** Both before and after watching the slideshow, participants responded to the prompt “Please indicate how you feel at this moment” using a 100-point scale (0 = I don’t feel this way, 100 = I very much feel this way). We averaged their responses to nine emotion words: happy, energetic, sad*, tense*, confused*, afraid*, tired*, angry*, guilty* (* denotes items that were reverse-scored). The no-CV control condition only completed this measure once. Alpha = .85 (mood 1), .81 (mood 2).

**Concern [Concern].** Participants rated 14 items (listed below) in response to the prompt “How concerned are you with the following environmental problems?”, using a 5-point scale (1 = Not at all concerned, 5 = Very concerned). Alpha = .94.

**Concern Mentioned [ConcernMentioned]** included the average of 6 items: deforestation, water drought, use of fossil fuels, air pollution, water pollution, loss of biodiversity. Alpha = .90.

**Concern Not Mentioned [ConcernNotMentioned]** included the average of 8 items: rising temperatures, trash in the oceans, sea level rise, mountaintop renewal, overpopulation, nuclear waste spill, non-sustainable farming practices, factory farmed animals. Alpha = .89.

**Commit [Commit].** Participants rated 12 items (listed below) in response to the prompt “How committed are you to the following actions?”, using a 5-point scale (1 = Not at all committed, 5 = Very committed). Alpha = .91.

**Committed Mentioned** **[CommitMentioned]** was an average of 5 items: eating local foods/growing your own food, bicycling or walking, conserving water, installing energy efficient appliances, volunteering to help your community. Alpha = .78.

**Committed Not Mentioned [CommitNotMentioned]** was an average of 7 items: Changing your dietary habits in ways that support the environment, recycling, shopping locally, using biodegradable products, ,voting, supporting candidates who champion the environment, educating others. Alpha = .85.

**Efficacy, Responsibility, Optimism, and Norms.** Participants responded to multiple items (listed below) in response to the prompt “Rate the degree to which you agree with the following statements”, on a 5-point scale (1 = Strongly disagree, 5 = Strongly agree).

**Efficacy & Responsibility [efficacy_responsibility]** was an average of 6 items: I can do things to make the environment better, People can work together to protect the environment, I have a responsibility to protect the environment for future generations., People have a responsibility to protect the environment for future generations., What I do now affects the environment in the future, What people do now affects the environment in the future. Alpha = .91.

**Optimism [Optimism]** was measured with the single item “I think the environment will be better in the future”.

**Youth Norms [Youth]** was an average of 4 items: I am aware of what youth think about the environmental issues, I am influenced by what youth think about environmental issues, youth are concerned about the environment, youth are taking action to protect the environment. Alpha = .77.

**Adult Norms [Adult]** was an average of 4 items: I am aware of what others think about environmental issues, I am influenced by what others think about environmental issues, other people are concerned about the environment, other people are taking action to protect the environment**.** Alpha = .59.

**Operational Definitions for Community Voices Study 2**

**Conditions**

**Community Voices vs No Community Voices [CV_check]:** Roughly 1/5 of all participants were randomly assigned to a no-exposure control condition. The rest watched one of 4 versions of the Community Voices slideshow (described below).

**Adult vs. Child [Source]**: This condition was manipulated by the images shown in the slide shows. Each slide show contains 16 images. All images were attributed to a fictional person who was either issued a grade (3^rd^-6^th^) in order to infer that they were a child or they were left blank with only a name shown (inferring adult). Additionally, of the 16 images, 6 were images of natural environments while the remaining 10 were of either adults or children matched along number of people, race, gender, activity in photo, and quality of photo. All quotes were controlled that they could be plausibly said by either an adult or a 5^th^ grader.

**Present vs. Future [Tense]:** This condition was manipulated by the messages that appeared on each slide. For those in the Future condition, phrases such as "in the future", "for years", "down the road" or "later on" were added to the messaging on the 10 slides that depicted people. Otherwise the content was identical. See Table S2 for the text of the messages.

**Table S2.** Messages appearing on Community Voices slides, by condition. Each message was paired with a picture of an adult and a child.

| PRESENT CONDITION | |
| --- | --- |
| Madeline L. | Keep remaking, keep reusing, to keep the world clean |
| Annie B. | Everything we need to live is here on earth. We just have to share it so we can all use it |
| Justin B. | Our planet will stay healthy when we create as little trash as possible |
| Elizabeth S. | I think rivers are beautiful |
| Ashley W. | We will build this community by bringing people together |
| George H. | Get outside and ride your bike! Keep the air clean |
| Anna C. | What affects your community affects you |
| Josh K. | Sustainability is what we need to be happy and healthy |
| Laura H. | We need a place to be outside and have fun |
| Nicole J. | I enjoy watching the fish swim down the stream |
| Lori G. | I like learning about the environment in school |
| Phil M. | It's good for our school to use renewable energy |
| Bobby M. | We should protect the earth because our land is for everyone: humans, plants, and animals |
| FUTURE CONDITION | |
| Madeline L. | Keep remaking, keep reusing to keep the world clean in the future |
| Annie B. | Everything we need to live is here on earth. We just have to share it so our kids can use it when they grow up |
| Justin B. | Our planet will stay healthy for years when we create as little trash as possible |
| Elizabeth S. | I think rivers are beautiful |
| Ashley W. | We build this community for years by bring people together |
| George H. | Get outside and ride your bike! Keep the air clean down the road |
| Anna C. | What affects your community affects you later on |
| Josh K. | Sustainability is what our kids need to be happy and healthy when they grow up |
| Laura H. | Kids in the future will need a place to be outside and have fun |
| Nicole J. | I enjoy watching the fish swim down the stream |
| Lori G. | In the future, I want my kids to be able to learn about the environment in school |
| Phil M. | It's good for my kid's school to use renewable energy for what lies ahead |
| Bobby M. | In the future, we should protect the Earth because our land is for everyone: humans, plants, and animals |

**Covariates**

**Connectedness to Nature [CNS].** In response to the prompt “Rate the degree to which you agree with the following statements” participants responded on a 5-point scale (1 = Strongly disagree, 5 = Strongly agree) to 5 items: I often feel a strong connection to nature, I think of nature as a family I belong to, I see myself as a part of the greater circle of life, I feel that all living things in this world are connected and I am a part of that, like the trees in the forest I feel I belong to nature. The items were averaged together, alpha = .93.

**Political Orientation [PoliScale].** Participants responded to the item “Use the scale below to indicate the extent to which you view yourself as politically liberal or conservative,” with 1 = Liberal, and 7 = Conservative.

**Manipulation Checks.**

To evaluate whether participants paid sufficient attention to the content of the slideshow, we asked two questions (described below) with the prompt “Please indicate your agreement to the following statements below” on a 5-point scale (1 = Strongly disagree, 5 = Strongly agree).

**Present vs future tense [mancheck_future]** was evaluated with the item “The slides I watched were about the future”.

**Child vs adult spokesperson** **[mancheck_adult]** was evaluated with the item “The quotes I read came from adults.”

**Dependent Variables**

**Concern [Concern].** Participants rated 14 items (listed below) in response to the prompt “How concerned are you with the following environmental problems?”, using a 5-point scale (1 = Not at all concerned, 5 = Very concerned). Alpha = .94.

**Concern Mentioned [ConcernMentioned]** included the average of 4 items: use of fossil fuels, trash in the oceans, air pollution, loss of biodiversity. Alpha = .85.

**Concern Not Mentioned [ConcernNotMentioned]** included the average of 10 items: rising temperatures, deforestation, sea level rise, water pollution, water drought, mountaintop removal, overpopulation, nuclear waste spill, non-sustainable farming practices, factory farmed animals. Alpha = .91.

**Commit [Commit].** Participants rated 13 items (listed below) in response to the prompt “How committed are you to the following actions?”, using a 5-point scale (1 = Not at all committed, 5 = Very committed). Alpha = .91.

**Committed Mentioned** **[CommitMentioned]** was an average of 4 items: Bicycling/walking, recycling, volunteering to help your community, meeting with fellow citizens to bring about political change. Alpha = .69.

**Committed Not Mentioned [CommitNotMentioned]** was an average of 9 items: Eating/growing local foods, changing dietary habits, shopping locally, using biodegradable products, voting, supporting candidates who champion the community, meet with fellow citizens to bring about political change. Alpha = .88.

**Efficacy, Responsibility, Optimism, and Norms.** Participants responded to multiple items (listed below) in response to the prompt “Rate the degree to which you agree with the following statements”, on a 5-point scale (1 = Strongly disagree, 5 = Strongly agree).

**Efficacy & Responsibility [efficacy_responsibility]** was an average of 6 items: I can do things to make the environment better, People can work together to protect the environment, I have a responsibility to protect the environment for future generations., People have a responsibility to protect the environment for future generations., What I do now affects the environment in the future, What people do now affects the environment in the future. Alpha = .92.

**Optimism [Optimism]** was measured with the single item “I think the environment will be better in the future”.

**Youth Norms [Youth]** was an average of 4 items: I am aware of what youth think about the environmental issues, I am influenced by what youth think about environmental issues, youth are concerned about the environment, youth are taking action to protect the environment. Alpha = .84.

**Adult Norms [Adult]** was an average of 4 items: I am aware of what others think about environmental issues, I am influenced by what others think about environmental issues, other people are concerned about the environment, other people are taking action to protect the environment**.** Alpha = .79.

**Empathy [Empathy].** Using a 5-point scale (1 = Strongly disagree, 5 = Strongly agree), participants responded to the prompt “Please think about how you felt when you watched the slides” to three items: I felt compassion, I felt concern, I felt touched by the people in the slides. We averaged the three items together, alpha = .88.

**Operational Definitions for Community Voices Study 3**

**Conditions**

**Adult vs. Child [Source]**: This condition was manipulated by the images shown in the slide shows. Each slide show contains 13 images. All images were attributed to a fictional person who was either issued a grade (3^rd^-6^th^) in order to infer that they were a child or they were left blank with only a name shown (inferring adult). Additionally, of the 13 images, 5 were images of natural environments while the remaining 8 were of either adults or children matched along number of people, race, gender, activity in photo, and quality of photo. All quotes were controlled that they could be plausibly said by either an adult or a 5^th^ grader. See Table S3 for the messages.

**Local vs Nonlocal [Locality]:** Participants were either recruited from MTurk (a nonlocal sample) or recruited from Oberlin, OH (a local sample).

**Table S3.** Messages appearing on Community Voices slides, by condition. Each message was paired with a picture of an adult and a child.

| Slide Title | Quote |
| --- | --- |
| Natural World | When I think of sustainability, I think of being caregivers of our earth, of our planet, of our home |
| Next Generation | If it’s a nice day, why not be outside |
| Natural World | Animals are relatable and they deserve to have healthy lives just like human beings do |
| Next Generation | Everyone needs to become aware with how we affect our environment |
| Natural World | Saving paper saves trees. Saving trees saves people |
| Next Generation | Our soil is living, it's alive, it's a life. It's our job to be stewards |
| Natural World | We have to take care of the environment because this is the only Earth we have to live on |
| Next Generation | Human energy is really important to sustainability |
| Natural World | If we are careful about preserving wetlands, water will be safe for plants and animals |
| Next Generation | I always think about how much energy me and my family use. Now it's time to do something about it |
| Next Generation | If we didn't have the environment, we wouldn't be alive right now |
| Heritage | Sustainability is going back to the old ways… Just simplifying your life |
| Next Generation | What we do, even though it seems small, can have a big effect on the world |

**Covariates**

**Connectedness to Nature [CNS].** In response to the prompt “Rate the degree to which you agree with the following statements” participants responded on a 5-point scale (1 = Strongly disagree, 5 = Strongly agree) to 5 items: I often feel a strong connection to nature, I think of nature as a family I belong to, I see myself as a part of the greater circle of life, I feel that all living things in this world are connected and I am a part of that, like the trees in the forest I feel I belong to nature. The items were averaged together, alpha = .93.

**Political Orientation [PoliScale].** Participants responded to the item “Use the scale below to indicate the extent to which you view yourself as politically liberal or conservative,” with 1 = Liberal, and 7 = Conservative.

**Manipulation Checks**

**Child vs adult spokesperson** **[man_check]** To evaluate whether participants paid sufficient attention to the content of the slideshow, we presented the item “The quotes I read came from adults.” Participants responded on a 5-point scale (1 = Strongly disagree, 5 = Strongly agree).

**Familiarity with people and places in slideshow** [familiar_p, familiar_o] Participants were asked “Did you recognize any of the people in the slideshow?” and “ Did you recognize in of the places in the slide show?” They responded on a 6-point scale from 0 = I didn’t recognize any person/place to 5 = I recognized more than one person/place”.

**Dependent Variables**

**Concern [Concern].** Participants rated 14 items (listed below) in response to the prompt “How concerned are you with the following environmental problems?”, using a 5-point scale (1 = Not at all concerned, 5 = Very concerned). Alpha = .94.

**Commit [Commit].** Participants rated 13 items (listed below) in response to the prompt “How committed are you to the following actions?”, using a 5-point scale (1 = Not at all committed, 5 = Very committed). Alpha = .92.

**Efficacy, Responsibility, Optimism, and Norms.** Participants responded to multiple items (listed below) in response to the prompt “Rate the degree to which you agree with the following statements”, on a 5-point scale (1 = Strongly disagree, 5 = Strongly agree).

**Efficacy & Responsibility [efficacy_responsibility]** was an average of 6 items: I can do things to make the environment better, People can work together to protect the environment, I have a responsibility to protect the environment for future generations., People have a responsibility to protect the environment for future generations., What I do now affects the environment in the future, What people do now affects the environment in the future. Alpha = .89.

**Optimism [Optimism]** was measured with the single item “I think the environment will be better in the future”.

**Youth Norms [Youth]** was an average of 4 items: I am aware of what youth think about the environmental issues, I am influenced by what youth think about environmental issues, youth are concerned about the environment, youth are taking action to protect the environment. Alpha = .77.

**Adult Norms [Adult]** was an average of 4 items: I am aware of what others think about environmental issues, I am influenced by what others think about environmental issues, other people are concerned about the environment, other people are taking action to protect the environment**.** Alpha = .70.

**Empathy [Empathy].** Using a 5-point scale (1 = Strongly disagree, 5 = Strongly agree), participants responded to the prompt “Please think about how you felt when you watched the slides” to three items: I felt compassion, I felt concern, I felt touched by the people in the slides. We averaged the three items together, alpha = .85.
