# Supplementary material for: Novel approach to delivering pro-environmental messages significantly shifts norms and motivation, but children are not more effective spokespeople than adults
Source: PLoS One. 2021 Sep 8;16(9):e0255457. doi: 10.1371/journal.pone.0255457 (PMC8425541; doi:10.1371/journal.pone.0255457)
Supplement: S3 Text — (DOCX) [file pone.0255457.s003.docx]

**S3: Summaries of Study 1 analyses with and without covariates**

This document contains summaries of the analyses relevant to our hypotheses, both with and without covariates. The syntax and all SPSS output can be found at <https://doi.org/10.5061/dryad.np5hqbzs4>.

**Table S3.1. Results of analyses testing the effects of Community Voices (compared to the no exposure control condition), controlling for participants’ connectedness to nature and political orientation.**

| Variable | Community Voices  N = 733 | No Community Voices  N = 88 | ­­F | p | Partial eta squared |
| --- | --- | --- | --- | --- | --- |
|  | Mean (SE) | Mean (SE) |  |  |  |
| Concern, overall | 3.60 (0.03) | 3.31 (0.08) | 13.19 | <.01** | 0.02 |
| Concern, mentioned in CV | 3.80 (0.03) | 3.44 (0.08) | 17.99 | <.01** | 0.02 |
| Concern, not mentioned in CV | 3.46 (0.03) | 3.21 (0.08) | 8.56 | <.01** | 0.01 |
| Behavioral commitment, overall | 5.85 (0.05) | 5.67 (0.13) | 1.69 | 0.19 | -- |
| Behavioral commitment, mentioned in CV | 5.80 (0.05) | 5.60 (0.14) | 1.91 | 0.17 | -- |
| Behavioral commitment, not mentioned in CV | 5.89 (0.05) | 5.72 (0.15) | 1.25 | 0.27 | -- |
| Efficacy & responsibility^1^ | 4.23 (0.02) | 4.13 (0.06) | 5.5 | .02** | 0.01 |
| Optimism | 2.98 (0.04) | 2.93 (0.11) | 0.2 | 0.66 | -- |
| Perceived norms, children | 3.44 (0.03) | 3.20 (0.07) | 11.9 | <.01** | 0.01 |
| Perceived norms, adult | 3.69 (0.02) | 3.60 (0.06) | 3.01 | .08* | 0.004 |

. ** = significant at the .05 level, * = significant at the .10 level

**Table S3.2. Results of analyses testing the effects of Community Voices (compared to the no exposure control condition) without covariates.**

| Variable | Community Voices  N = 767 | No Community Voices  N = 97 | ­­F | p | Partial eta squared |
| --- | --- | --- | --- | --- | --- |
|  | Mean (SE) | Mean (SE) |  |  |  |
| Concern, overall | 3.59 (0.03) | 3.33 (0.09) | 6.77 | .01** | .01 |
| Concern, mentioned in CV | 3.78 (0.03) | 3.46 (0.09) | 10.25 | < .01** | .01 |
| Concern, not mentioned in CV | 3.44 (0.03) | 3.23 (0.10) | 4.19 | .04** | .01 |
| Behavioral commitment, overall | 5.84 (0.06) | 5.72 (0.15) | 0.53 | .47 | -- |
| Behavioral commitment, mentioned in CV | 5.79 (0.06) | 5.64 (0.16) | 0.77 | .38 | -- |
| Behavioral commitment, not mentioned in CV | 5.82 (0.06) | 5.65 (0.17) | 0.9 | .34 | -- |
| Efficacy & responsibility^1^ | 4.23 (0.02) | 4.10 (0.07) | 3.6 | .06* | .01 |
| Optimism | 2.98 (0.04) | 2.91 (0.10) | 0.53 | .47 | -- |
| Perceived norms, children | 3.44 (0.03) | 3.19 (0.08) | 9.69 | < .01** | .01 |
| Perceived norms, adult | 3.70 (0.02) | 3.59 (0.06) | 3.16 | .08* | .01 |

. ** = significant at the .05 level, * = significant at the .10 level

Removing the covariates resulted in only one change. The effect of Community Voices on efficacy and responsibility was significant with the covariate, but became only marginally significant (p < .1) without the covariate. No other variables changed in significance with the removal of the covariates.

**Table S3.3. Summary of 2 (adult vs child messenger) by 2 (injunctive vs noninjunctive) ANCOVAs with connectedness to nature and political orientation as covariates.**

| Variable | Noninjunctive, Adult | Injunctive, Adult | Noninjunctive, Child | Injunctive, Child | Main effect, messenger  F | Main effect, Injunctive  F | Messenger x Injunctive Interaction  F |
| --- | --- | --- | --- | --- | --- | --- | --- |
|  | Mean (SE) | Mean (SE) | Mean (SE) | Mean (SE) |  |  |  |
| Concern, overall | 3.60 (0.05) | 3.59 (0.05) | 3.64 (0.05) | 3.55 (0.05) | 0.00 | 0.82 | 0.43 |
| Concern, mentioned in CV | 3.81 (0.06) | 3.78 (0.06) | 3.84 (0.05) | 3.72 (0.06) | 0.09 | 1.69 | 0.69 |
| Convern, not mentioned in CV | 3.44 (0.06) | 3.44 (0.06) | 3.49 (0.05) | 3.43 (0.06) | 0.10 | 0.31 | 0.23 |
| Commitment, overall | 5.77 (0.09) | 5.81 (0.09) | 5.94 (0.09) | 5.82 (0.09) | 0.98 | 0.17 | 0.78 |
| Commitment, mentioned in CV | 5.71 (0.10) | 5.80 (0.10) | 5.91 (0.09) | 5.73 (0.10) | 0.46 | 0.26 | 1.84 |
| Commitment, not mentioned in CV | 5.75 (0.10) | 5.78 (0.10) | 5.91 (0.10) | 5.83 (0.10) | 1.21 | 0.07 | 0.29 |
| Efficacy & responsibility | 4.21 (0.04) | 4.27 (0.04) | 4.22 (0.04) | 4.23 (0.04) | 0.04 | 0.76 | 0.38 |
| Optimism | 2.99 (0.07) | 2.94 (0.07) | 3.08 (0.07) | 2.88 (0.07) | 0.03 | 2.89 | 1.07 |
| Perceived norms, children | 3.33 (0.05) | 3.33 (0.05) | 3.56 (0.05) | 3.55 (0.05) | 20.29** | 0.03 | 0.02 |
| Perceived norms, adult | 3.70 (0.04) | 3.69 (0.04) | 3.70 (0.04) | 3.68 (0.04) | 0.01 | 0.22 | 0.01 |

. ** = significant at the .05 level, * = significant at the .10 level

**Table S3.4. Summary of 2 (adult vs child messenger) by 2 (injunctive vs noninjunctive) ANOVAs without covariates.**

| Variable | Noninjunctive, Adult | Injunctive, Adult | Noninjunctive, Child | Injunctive, Child | Main effect, messenger  F | Main effect, Injunctive  F | Messenger x Injunctive Interaction  F |
| --- | --- | --- | --- | --- | --- | --- | --- |
|  | Mean (SE) | Mean (SE) | Mean (SE) | Mean (SE) |  |  |  |
| Concern, overall | 3.58 (0.07) | 3.51 (0.07) | 3.67 (0.06) | 3.58 (0.07) | 1.73 | 1.53 | 0.03 |
| Concern, mentioned in CV | 3.78 (0.07) | 3.70 (0.07) | 3.87 (0.07) | 3.75 (0.07) | 1.13 | 2.22 | 0.10 |
| Convern, not mentioned in CV | 3.42 (0.07) | 3.36 (0.07) | 3.53 (0.07) | 3.45 (0.07) | 2.06 | 1.00 | 0.01 |
| Commitment, overall | 5.76 (0.11) | 5.68 (0.11) | 6.00 (0.11) | 5.90 (0.11) | 4.40** | 0.70 | 0.00 |
| Commitment, mentioned in CV | 5.72 (0.11) | 5.65 (0.11) | 5.96 (0.11) | 5.81 (0.11) | 3.06* | 0.92 | 0.15 |
| Commitment, not mentioned in CV | 5.74 (0.12) | 5.63 (0.12) | 5.97 (0.12) | 5.91 (0.12) | 4.62** | 0.53 | 0.06 |
| Efficacy & responsibility | 4.20 (0.05) | 4.21 (0.05) | 4.25 (0.05) | 4.25 (0.05) | 0.83 | 0.02 | 0.00 |
| Optimism | 3.04 (0.07) | 2.93 (0.07) | 3.07 (0.07) | 2.88 (0.07) | 0.01 | 4.47** | 0.28 |
| Perceived norms, children | 3.35 (0.05) | 3.27 (0.05) | 3.58 (0.05) | 3.55 (0.05) | 23.26** | 1.17 | 0.22 |
| Perceived norms, adult | 3.72 (0.04) | 3.66 (0.04) | 3.72 (0.04) | 3.69 (0.04) | 0.14 | 1.14 | 0.16 |

. ** = significant at the .05 level, * = significant at the .10 level

Removing the covariates resulted in several changes. The main effects of the messenger on overall commitment, and commitment to those not mentioned in CV became significant. The amin effect of messenger on commitment to those issues mentioned in CV became marginally significant without covariates. In all cases, child messengers resulted in higher levels of commitment.

Additionally, the main effect of injunctive messages on optimism became significant without covariate. Participants reported higher levels of optimism in the non-injunctive condition. No other variables had changes in significance with the removal of covariates.

**Table S3.5. Summary of 2 (adult vs child messenger) by 2 (political vs nonpolitical) ANCOVAs with connectedness to nature and political orientation as covariates.**

| Variable | Nonpolitical, Adult | Political, Adult | Nonpolitical, Child | Political, Child | Main effect, messenger  F | Main effect, Political  F | Messenger x Political Interaction  F |
| --- | --- | --- | --- | --- | --- | --- | --- |
|  | Mean (SE) | Mean (SE) | Mean (SE) | Mean (SE) |  |  |  |
| Concern, overall | 3.56 (0.05) | 3.63 (0.05) | 3.58 (0.05) | 3.61 (0.05) | 0.00 | 1.10 | 0.12 |
| Concern, mentioned in CV | 3.76 (0.06) | 3.83 (0.06) | 3.77 (0.06) | 3.79 (0.05) | 0.09 | 0.69 | 0.23 |
| Convern, not mentioned in CV | 3.40 (0.05) | 3.48 (0.06) | 3.43 (0.06) | 3.48 (0.05) | 0.10 | 1.29 | 0.04 |
| Commitment, overall | 5.74 (0.09) | 5.84 (0.09) | 5.82 (0.09) | 5.95 (0.09) | 0.98 | 1.59 | 0.03 |
| Commitment, mentioned in CV | 5.70 (0.10) | 5.81 (0.10) | 5.79 (0.10) | 5.85 (0.09) | 0.46 | 0.70 | 0.10 |
| Commitment, not mentioned in CV | 5.73 (0.10) | 5.79 (0.10) | 5.79 (0.10) | 5.95 (0.10) | 1.21 | 1.38 | 0.26 |
| Efficacy & responsibility | 4.24 (0.04) | 4.23 (0.04) | 4.18 (0.04) | 4.28 (0.04) | 0.04 | 1.42 | 1.75 |
| Optimism | 3.04 (0.07) | 2.89 (0.07) | 3.01 (0.07) | 2.95 (0.07) | 0.03 | 2.21 | 0.36 |
| Perceived norms, children | 3.35 (0.05) | 3.30 (0.05) | 3.53 (0.05) | 3.57 (0.05) | 20.29** | 0.01 | 0.85 |
| Perceived norms, adult | 3.73 (0.04) | 3.66 (0.04) | 3.68 (0.04) | 3.69 (0.04) | 0.01 | 0.44 | 0.80 |

. ** = significant at the .05 level, * = significant at the .10 level

**Table S3.6. Summary of 2 (adult vs child messenger) by 2 (political vs nonpolitical) ANOVAs without covariates.**

| Variable | Nonpolitical, Adult | Political, Adult | Nonpolitical, Child | Political, Child | Main effect, messenger  F | Main effect, Political  F | Messenger x Political Interaction  F |
| --- | --- | --- | --- | --- | --- | --- | --- |
|  | Mean (SE) | Mean (SE) | Mean (SE) | Mean (SE) |  |  |  |
| Concern, overall | 3.52 (0.07) | 3.57 (0.07) | 3.59 (0.07) | 3.66 (0.06) | 1.73 | 0.85 | 0.02 |
| Concern, mentioned in CV | 3.72 (0.07) | 3.77 (0.07) | 3.79 (0.07) | 3.84 (0.06) | 1.13 | 0.62 | 0.01 |
| Convern, not mentioned in CV | 3.36 (0.07) | 3.42 (0.07) | 3.45 (0.07) | 3.53 (0.07) | 2.06 | 0.97 | 0.04 |
| Commitment, overall | 5.69 (0.11) | 5.75 (0.11) | 5.85 (0.11) | 6.05 (0.11) | 4.40** | 1.42 | 0.41 |
| Commitment, mentioned in CV | 5.64 (0.11) | 5.73 (0.11) | 5.83 (0.12) | 5.94 (0.11) | 3.06* | 0.77 | 0.01 |
| Commitment, not mentioned in CV | 5.68 (0.12) | 5.69 (0.12) | 5.82 (0.12) | 6.06 (0.12) | 4.62** | 1.1 | 0.9 |
| Efficacy & responsibility | 4.21 (0.05) | 4.20 (0.05) | 4.18 (0.05) | 4.31 (0.05) | 0.83 | 1.79 | 2.35 |
| Optimism | 3.06 (0.07) | 2.91 (0.07) | 3.01 (0.07) | 2.95 (0.07) | 0.01 | 1.98 | 0.41 |
| Perceived norms, children | 3.34 (0.05) | 3.28 (0.05) | 3.54 (0.05) | 3.59 (0.05) | 23.26** | 0.01 | 1.21 |
| Perceived norms, adult | 3.72 (0.04) | 3.65 (0.04) | 3.69 (0.04) | 3.72 (0.04) | 0.14 | 0.26 | 1.39 |

. ** = significant at the .05 level, * = significant at the .10 level

As reported above, the main effects of messenger on the commitment variables became significant or marginally significant. No other changes in results were observed.
