# Supplementary material for: Novel approach to delivering pro-environmental messages significantly shifts norms and motivation, but children are not more effective spokespeople than adults
Source: PLoS One. 2021 Sep 8;16(9):e0255457. doi: 10.1371/journal.pone.0255457 (PMC8425541; doi:10.1371/journal.pone.0255457)
Supplement: S4 Text — (DOCX) [file pone.0255457.s004.docx]

**S4: Summaries of Study 2 analyses with and without covariates**

This document contains summaries of the analyses relevant to our hypotheses, both with and without covariates. The syntax and all SPSS output can be found at <https://doi.org/10.5061/dryad.np5hqbzs4>.

**Table S4.1. Results of analyses testing the effects of Community Voices (compared to the no exposure control condition) controlling for participants’ connectedness to nature and political orientation.**

| Variable | Community Voices  N = 421 | No Community Voices  N = 82 | F | p | Eta squared |
| --- | --- | --- | --- | --- | --- |
|  | Mean (SE) | Mean (SE) |  |  |  |
| Concern, overall | 3.62 (0.03) | 3.43 (0.07) | 5.25 | .02** | .01 |
| Concern, mentioned in CV | 3.74 (0.04) | 3.56 (0.08) | 3.95 | .05** | .01 |
| Concern, not mentioned in CV | 3.57 (0.03) | 3.38 (0.08) | 5.31 | .20 | -- |
| Commitment, overall | 3.07 (0.03) | 2.89 (0.08) | 4.25 | .04** | .01 |
| Commitment, mentioned in CV | 2.80 (0.04) | 2.65 (0.09) | 2.59 | .11 | -- |
| Commitment, not mentioned in CV | 3.19 (0.04) | 3.00 (0.08) | 3.92 | .05** | .01 |
| Efficacy & responsibility | 4.13 (0.03) | 4.06 (0.06) | 1.61 | .22 | -- |
| Optimism | 3.12 (0.05) | 2.82 (0.12) | 5.07 | .03** | .01 |
| Perceived norms, children | 3.46 (0.04) | 3.30 (0.09) | 2.66 | .10* | .005 |
| Perceived norms, adult | 3.96 (0.03) | 3.84 (0.07) | 2.35 | .13 | -- |

. ** = significant at the .05 level, * = significant at the .10 level

**Table S4.2. Results of analyses testing the effects of Community Voices (compared to the no exposure control condition) without covariates.**

| Variable | Community Voices  N = 426 | No Community Voices  N = 83 | F | p | Eta squared |
| --- | --- | --- | --- | --- | --- |
|  | Mean (SE) | Mean (SE) |  |  |  |
| Concern, overall | 3.62 (0.04) | 3.45 (0.10) | 2.76 | .10* | .005 |
| Concern, mentioned in CV | 3.74 (0.04) | 3.57 (0.10) | 2.32 | .13 | -- |
| Concern, not mentioned in CV | 3.57 (0.04) | 3.40 (0.10) | 2.78 | .10* | .005 |
| Commitment, overall | 3.07 (0.04) | 2.91 (0.10) | 2.1 | .15 | -- |
| Commitment, mentioned in CV | 2.80 (0.04) | 2.67 (0.10) | 1.57 | .21 | -- |
| Commitment, not mentioned in CV | 3.19 (0.05) | 3.03 (0.11) | 1.94 | .17 | -- |
| Efficacy & responsibility | 4.13 (0.03) | 4.07 (0.07) | 0.655 | .42 | -- |
| Optimism | 3.12 (0.05) | 2.84 (0.12) | 4.4 | .04** | .01 |
| Perceived norms, children | 3.45 (0.04) | 3.32 (0.10) | 1.45 | .23 | -- |
| Perceived norms, adult | 3.96 (0.03) | 3.85 (0.07) | 1.64 | .20 | -- |

. ** = significant at the .05 level, * = significant at the .10 level

The significance of several effects shifted when the covariates were removed. Overall concern went from significant to marginally significant (p < .1), Concern for issues mentioned in CV went from significant to nonsignificant, and concern for issues not mentioned in the slideshow went from nonsignificant to marginally significant (p < .1) with the removal of the covariates. Overall commitment went from significant at the .05 level to nonsignificant when covariates were removed. Perceived norms in children went from marginally significant to nonsignificant with the removal of the covariate.

The other three variables (commitment mentioned in CV, efficacy and responsibility, and optimism) did not change in significance with the removal of the covariates.

**S4.3. Summary of 2 (present vs future tense) by 2 (adult vs child messenger) ANCOVAs with connectedness to nature and political orientation as covariates.**

| Variable | Present, Child  N = 114 | Present, Adult  N = 111 | Future, Child  N = 99 | Future, Adult  N = 112 | Main effect, messenger  F | Main effect, Tense  F | Messenger x Tense Interaction  F |
| --- | --- | --- | --- | --- | --- | --- | --- |
|  | Mean (SE) | Mean (SE) | Mean (SE) | Mean (SE) |  |  |  |
| Concern, overall | 3.56 (0.06) | 3.68 (0.07) | 3.67 (0.07) | 3.56 (0.06) | 0.00 | 0.00 | 3.19* |
| Concern, mentioned in CV | 3.66 (0.07) | 3.82 (0.07) | 3.81 (0.07) | 3.67 (0.07) | 0.00 | 0.00 | 4.61** |
| Convern, not mentioned in CV | 3.52 (0.06) | 3.63 (0.07) | 3.62 (0.07) | 3.52 (0.06) | 0.01 | 0.00 | 2.32 |
| Commitment, overall | 3.04 (0.07) | 3.06 (0.07) | 3.17 (0.07) | 3.01 (0.07) | 1.13 | 0.43 | 1.80 |
| Commitment, mentioned in CV | 2.82 (0.07) | 2.76 (0.08) | 2.93 (0.08) | 2.71 (0.07) | 3.30* | 0.15 | 1.12 |
| Commitment, not mentioned in CV | 3.13 (0.07) | 3.19 (0.08) | 3.28 (0.08) | 3.14 (0.07) | 0.32 | 0.45 | 1.69 |
| Efficacy & responsibility | 4.09 (0.05) | 4.33 (0.06) | 4.19 (0.06) | 4.03 (0.05) | 0.05 | 0.75 | 7.23** |
| Optimism | 3.25 (0.10) | 3.08 (0.11) | 3.29 (0.11) | 2.86 (0.10) | 7.89** | 0.76 | 1.51 |
| Perceived norms, children | 3.57 (0.08) | 3.30 (0.08) | 3.71 (0.08) | 3.26 (0.08) | 21.68** | 0.39 | 1.14 |
| Perceived norms, adult | 4.03 (0.06) | 3.97 (0.06) | 4.00 (0.06) | 3.85 (0.06) | 2.89* | 1.51 | 0.46 |

. ** = significant at the .05 level, * = significant at the .10 level

**Table S4.4. Summary of 2 (present vs future tense) by 2 (adult vs child messenger) ANOVAs without covariates.**

| Variable | Present, Child  N = 114 | Future, Child  N = 99 | Present, Adult  N = 101 | Future, Adult  N = 112 | Main effect, messenger  F | Main effect, Tense  F | Messenger x Tense Interaction  F |
| --- | --- | --- | --- | --- | --- | --- | --- |
|  | Mean (SE) | Mean (SE) | Mean (SE) | Mean (SE) |  |  |  |
| Concern, overall | 3.54 (0.08) | 3.74 (0.09) | 3.73 (0.09) | 3.50 (0.08) | 0.08 | 0.01 | 6.77** |
| Concern, mentioned in CV | 3.64 (0.08) | 3.88 (0.09) | 3.86 (0.09) | 3.60 (0.09) | 0.10 | 0.02 | 8.09** |
| Convern, not mentioned in CV | 3.49 (0.08) | 3.69 (0.09) | 3.68 (0.09) | 3.46 (0.08) | 0.07 | 0.01 | 5.82** |
| Commitment, overall | 3.01 (0.08) | 3.25 (0.09) | 3.09 (0.09) | 2.95 (0.08) | 1.60 | 0.35 | 4.81** |
| Commitment, mentioned in CV | 2.80 (0.08) | 2.98 (0.09) | 2.79 (0.09) | 2.66 (0.08) | 3.68* | 0.10 | 3.25* |
| Commitment, not mentioned in CV | 3.10 (0.09) | 3.36 (0.10) | 3.22 (0.09) | 3.08 (0.09) | 0.74 | 0.41 | 4.71** |
| Efficacy & responsibility | 4.06 (0.06) | 4.24 (0.07) | 4.24 (0.07) | 4.00 (0.06) | 0.17 | 0.15 | 10.89** |
| Optimism | 3.25 (0.10) | 3.31 (0.11) | 3.08 (0.11) | 2.88 (0.10) | 7.77** | 0.47 | 1.52 |
| Perceived norms, children | 3.55 (0.08) | 3.75 (0.09) | 3.31 (0.09) | 3.23 (0.08) | 19.18** | 0.50 | 2.71* |
| Perceived norms, adult | 4.00 (0.06) | 4.02 (0.07) | 3.97 (0.07) | 3.84 (0.06) | 2.75* | 0.69 | 1.39 |

. ** = significant at the .05 level, * = significant at the .10 level

When the covariates were omitted, a number of messenger and tense interactions became significant. Overall concern went from marginally significant to very significant and concern for issues not mentioned in the slide show went from nonsignificant to significant at the p < 0.05 level. Further, the messenger by tense interaction for overall commitment went from nonsignificant to significant, commitment to issues mentioned in the slideshow went from nonsignificant to marginally significant, and commitment to issues not mentioned went from nonsignificant to significant at the p < 0.05 level. In all cases the pattern was the same: child messengers led to higher levels of concern and commitment in the future tense condition while adult messengers led to higher levels of concern and commitment in the present tense condition. These effects are all consistent with our hypothesis (Q3c) that child messengers might be more effective when talking about the future.

Additionally, the interaction effect of messenger and tense on perceived norms for children went from nonsignificant to marginally significant (p < 0.10) without the covariates included. The pattern of means suggests that child messengers in the future condition particularly increased perceived child norms.

The other variables (concern mentioned in CV, efficacy and responsibility, optimism, and perceived norms in adults) did not change in significance.

We planned *a priori* to test whether any beneficial effects of child messengers were mediated by higher levels of empathy for children. Child messengers marginally outperformed adult messengers on efficacy and responsibility in the future condition. Standard tests of mediation yielded marginal results (see Table S4.5 below), but the Sobel test evaluating the change in messenger regression weight after including the mediator (empathy) was significant, Sobel test = 2.87, p < .01. Child messengers also outperformed adult messengers on optimism. Mediation analyses suggest that this effect was also partially mediated by empathy (Sobel test = 2.65, p < .01). The Sobel tests were computed using the online calculator found at https://www.danielsoper.com/statcalc/calculator.aspx?id=31.

**Table S4.5. Summary of regression equations testing empathy as a mediator of the messenger effects on optimism and efficacy and responsibility among those in the future condition.**

| **Independent Variable** | **Dependent Variable** | **b** | **t** | **p** |
| --- | --- | --- | --- | --- |
| ^1^Messenger (child vs adult) | Empathy | -.36 | 3.06 | .003** |
| ^1^Messenger (child vs adult | Efficacy and responsibility | -.15 | 1.92 | .06* |
| ^1^Messenger (child vs adult) | Optimism | -.42 | 2.68 | .01** |
| Empathy | Efficacy and Responsibility | .34 | 8.36 | <.001** |
| ^1^Empathy  Messenger | Efficacy and Responsibility | .15  -.10 | 3.31  1.24 | .001**  .22 |
| Empathy | Optimism | .38 | 5.26 | <.001** |
| ^1^Empathy  Messenger | Optimism | .31  -.31 | 3.48  1.98 | .001**  .05** |

**^1^** Equation also controlled for CNS and political orientation. ** p < .05, * p < .10.
