# Supplementary material for: Novel approach to delivering pro-environmental messages significantly shifts norms and motivation, but children are not more effective spokespeople than adults
Source: PLoS One. 2021 Sep 8;16(9):e0255457. doi: 10.1371/journal.pone.0255457 (PMC8425541; doi:10.1371/journal.pone.0255457)
Supplement: S5 Text — (DOCX) [file pone.0255457.s005.docx]

**S5: Summaries of Study 3 analyses with and without covariates**

This document contains summaries of the analyses relevant to our hypotheses, both with and without covariates. The syntax and all SPSS output can be found at <https://doi.org/10.5061/dryad.np5hqbzs4>.

**Table S5.1. Results of 2 (adult vs child messenger) by 2 (local vs nonlocal audience) ANCOVAs with connectedness to nature and political orientation as covariates.**

| Variable | Local, Adult  N = 55 | Local, Child  N = 42 | Nonlocal, Adult  N = 144 | Nonlocal Child  N = 139 | Main effect, messenger  F | Main effect, audience  F | Messenger x Audience Interaction  F |
| --- | --- | --- | --- | --- | --- | --- | --- |
|  | Mean (SE) | Mean (SE) | Mean (SE) | Mean (SE) |  |  |  |
| Concern, overall | 4.03 (0.10) | 4.01 (0.12) | 3.82 (0.06) | 3.71 (0.06) | 0.77 | 5.83** | 0.25 |
| Commitment, overall | 3.69 (0.10) | 3.65 (0.12) | 3.57 (0.06) | 3.43 (0.06) | 1.46 | 2.65* | 0.51 |
| Efficacy & responsibility | 4.28 (0.07) | 4.25 (0.09) | 4.20 (0.04) | 4.16 (0.04) | 0.37 | 1.27 | 0 |
| Optimism | 2.81 (0.17) | 2.77 (0.20) | 3.01 (0.10) | 3.04 (0.10) | 0 | 1.61 | 0.06 |
| Perceived norms, children | 3.5 (0.12) | 3.60 (0.14) | 3.38 (0.07) | 3.45 (0.07) | 0.87 | 1.22 | 0.03 |
| Perceived norms, adult | 4.01 (0.09) | 3.94 (0.11) | 3.92 (0.05) | 3.94 (0.05) | 0.23 | 0.22 | 0.42 |

. ** = significant at the .05 level, * = significant at the .10 level

**Table S5.2. Results of 2 (adult vs child messenger) by 2 (local vs nonlocal audience) ANOVAs without covariates.**

| Variable | Local, Adult | Local, Child | Nonlocal, Adult | Nonlocal Child | Main effect, messenger  F | Main effect, audience  F | Messenger x Audience Interaction  F |
| --- | --- | --- | --- | --- | --- | --- | --- |
|  | Mean (SE)  N = 60 | Mean (SE)  N = 44 | Mean (SE)  N = 150 | Mean (SE)  N = 142 |  |  |  |
| Concern, overall | 4.35 (0.10) | 4.35 (0.12) | 3.69 (0.07) | 3.57 (0.07) | 0.43 | 59.11** | 0.397 |
| Commitment, overall | 4.03 (0.11) | 4.04 (0.12) | 3.43 (0.07) | 3.29 (0.07) | 0.48 | 51.55** | 0.57 |
| Efficacy & responsibility | 4.44 (0.07) | 4.42 (0.09) | 4.13 (0.05) | 4.08 (0.05) | 0.3 | 25.12** | 0.05 |
| Optimism | 2.8 (0.14) | 2.74 (0.17) | 2.99 (0.09) | 3.02 (0.09) | 0.01 | 3.31* | 0.1 |
| Perceived norms, children | 3.59 (0.10) | 3.67 (0.12) | 3.34 (0.07) | 3.42 (0.07) | 0.75 | 7.55** | 0 |
| Perceived norms, adult | 4.09 (0.08) | 4.07 (0.09) | 3.90 (0.05) | 3.88 (0.05) | 0.06 | 7.25** | 0 |

. ** = significant at the .05 level, * = significant at the .10 level

When the covariates were removed, several nonsignificant audience effects became significant. Specifically, the main effect of audience on efficacy and responsibility, perceived child norms, and perceived adult norms went from being nonsignificant with covariates to being highly significant (p < .001) without the covariates. Additionally, the main effect of audience on commitment went from nonsignificant to marginally significant, and optimism went from being nonsignificant with covariates to being marginally significant without the covariates (p < .1). In all cases save one (optimism), the Oberlin sample scored higher than the MTurk sample did.

It should be noted that the two samples differed in how they were obtained, and that they had many demographic differences. We believe it likely that the significant results in Table S5.2 stem from these demographic differences, rather than a differential impact of CV on the local audience.
