# Supplementary material for: Novel approach to delivering pro-environmental messages significantly shifts norms and motivation, but children are not more effective spokespeople than adults
Source: PLoS One. 2021 Sep 8;16(9):e0255457. doi: 10.1371/journal.pone.0255457 (PMC8425541; doi:10.1371/journal.pone.0255457)
Supplement: S1 Images — (ZIP) [file pone.0255457.s006.zip › Slideshows/Study 2 Slideshow Adults.pdf]

“Keep remaking, keep reusing, to keep the world clean.”
-Madeline L.

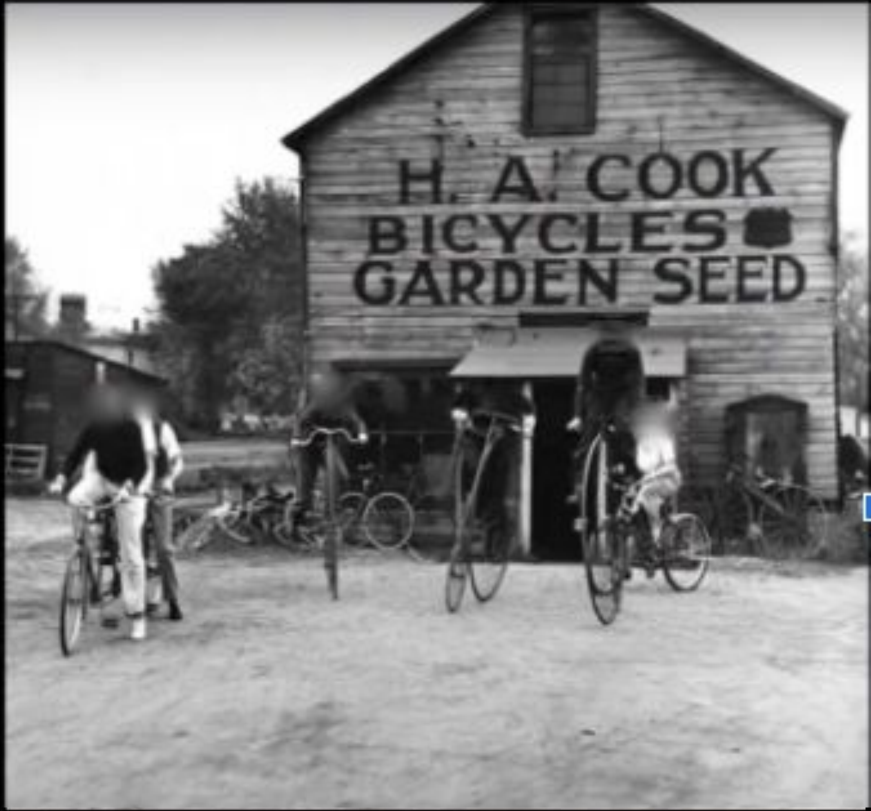

HERITAGE

"Everything we need to
live is here on earth.
We just have to share it
so we can all use it."
-Annie B.

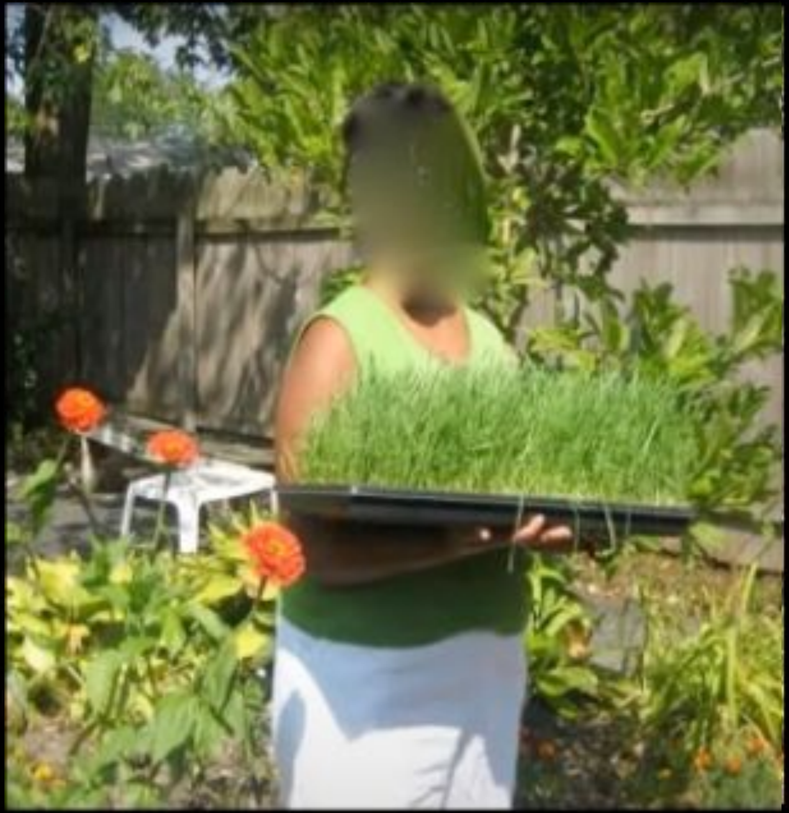

NEIGHBORS

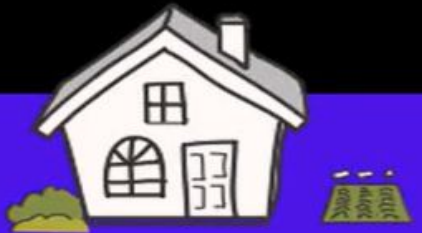

“Our planet will stay healthy when we create as little trash as possible.”

-Justin B.

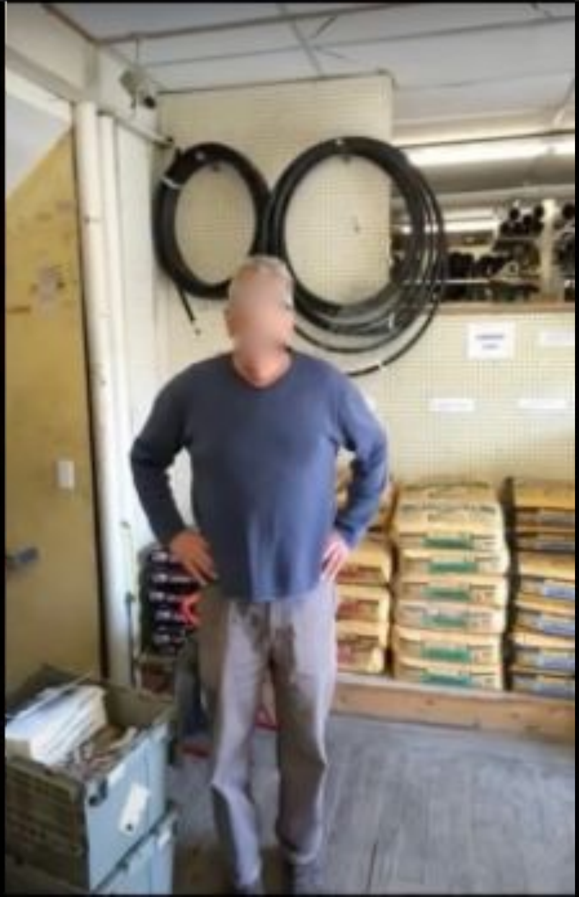

NEIGHBORS

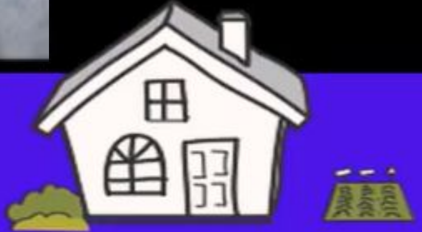

"I think rivers are beautiful."
-Elizabeth S.

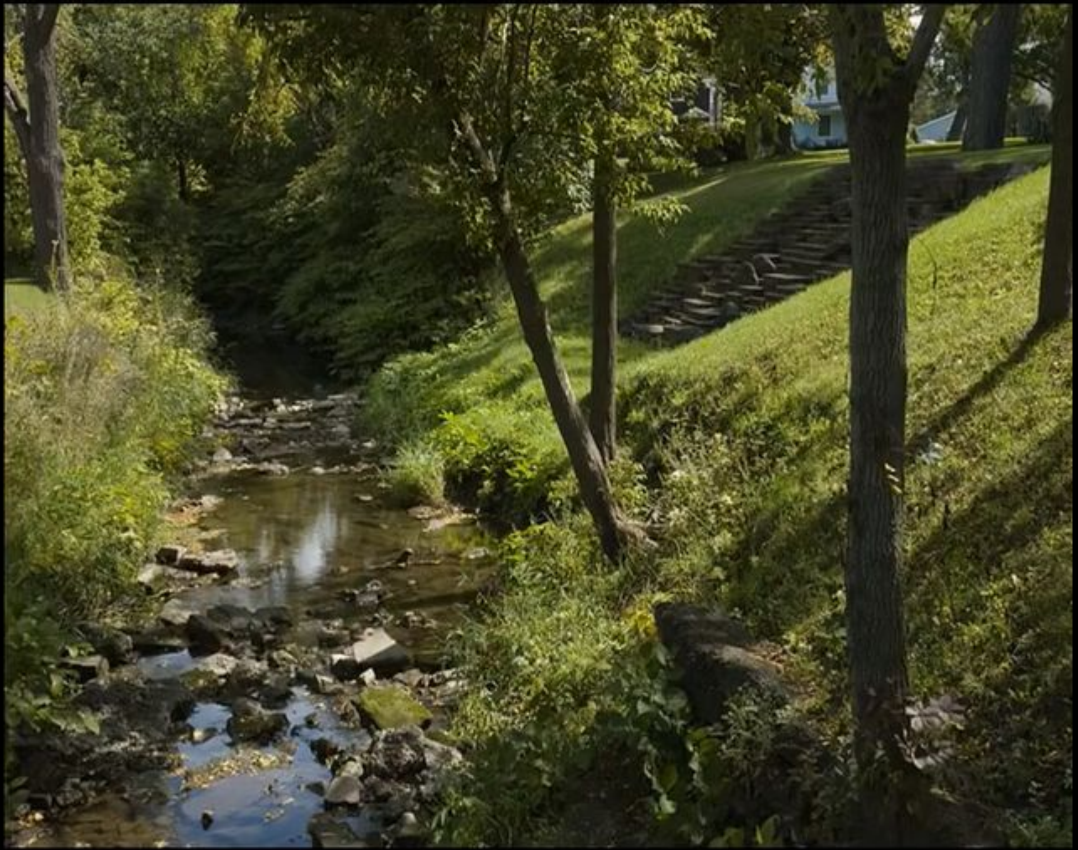

NATURAL WORLD

"We build this community and bring people together."
-Ashley W.

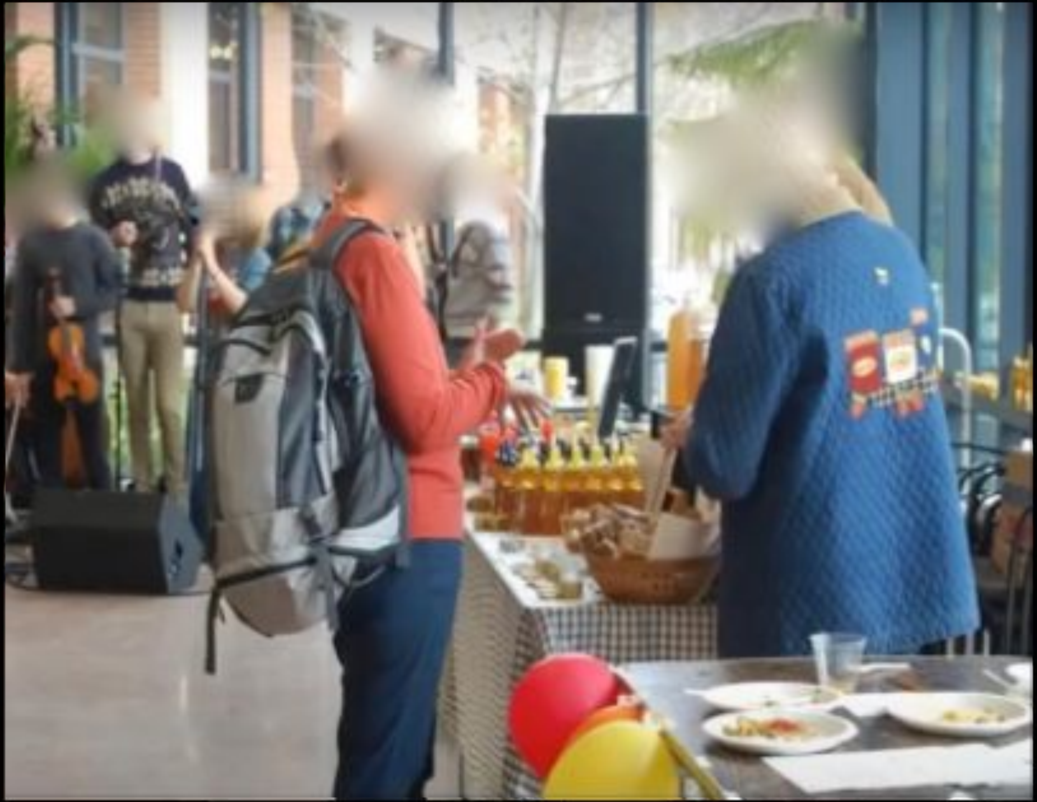

NEIGHBORS

“Get outside and ride your bike! Keep the air clean.”
-George H.

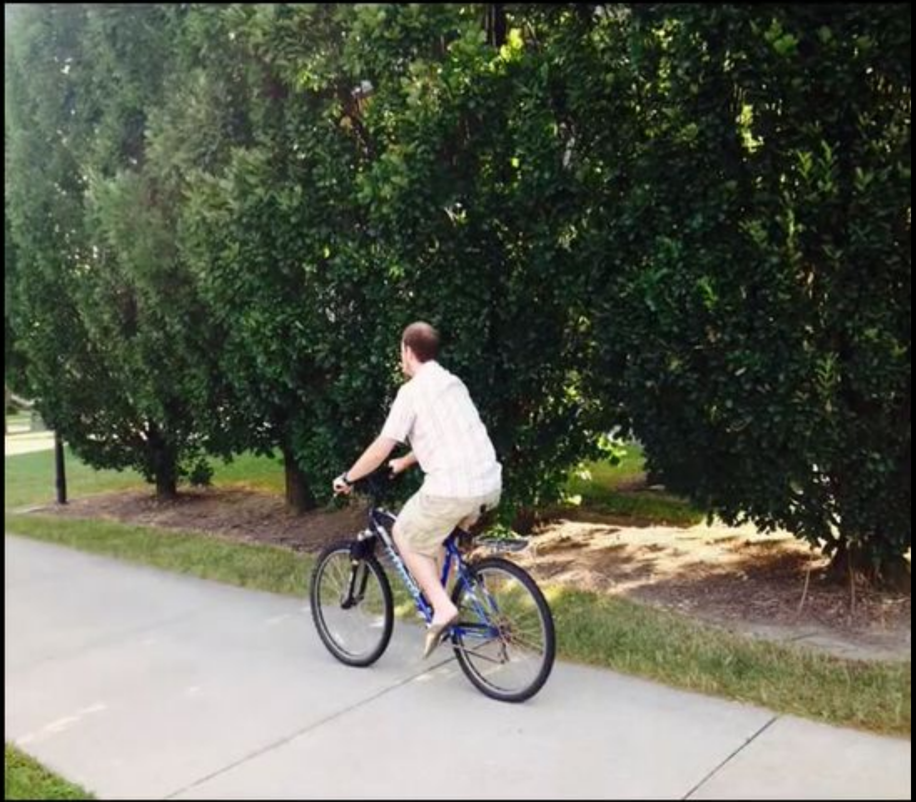

NEIGHBORS

"What affects your community affects you."
-Anna C.

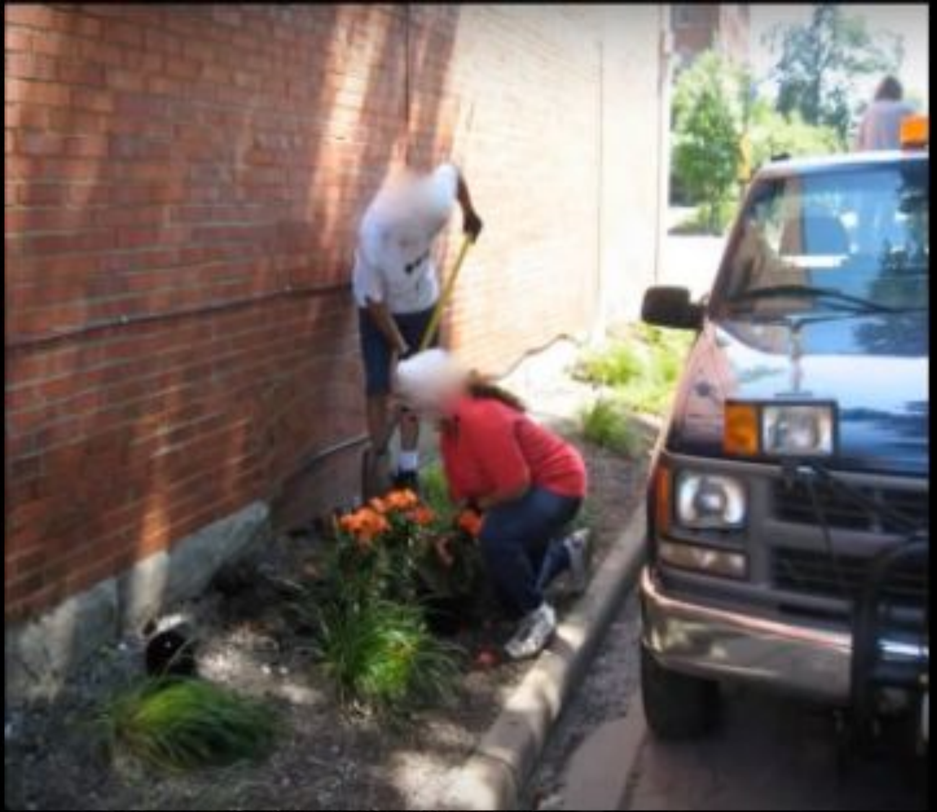

NEIGHBORS

“Sustainability is what our kids need to be happy and healthy.”
-Josh K.

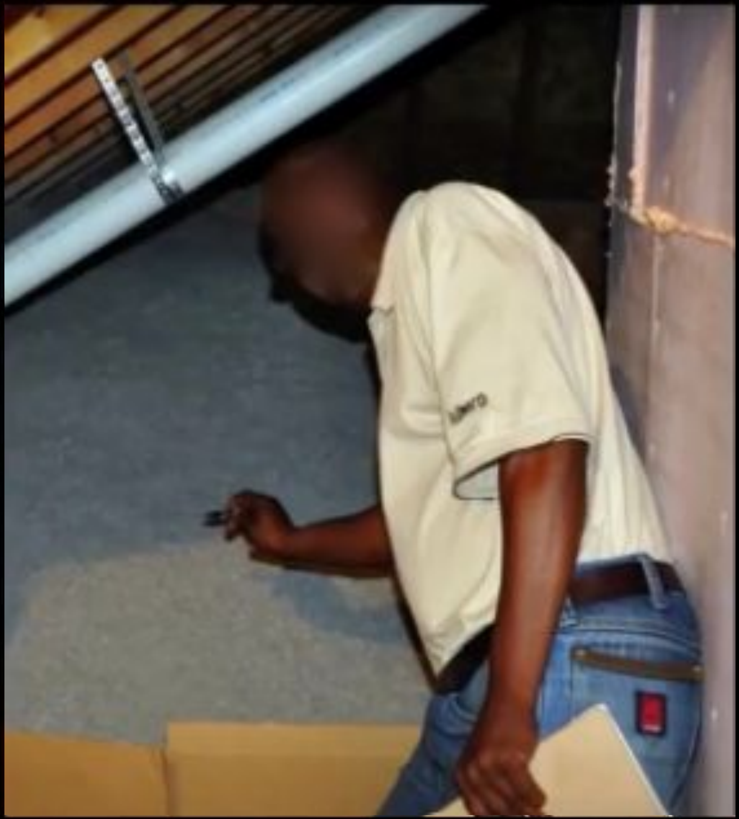

NEIGHBORS

"Kids need a place to be outside and have fun."
-Laura H.

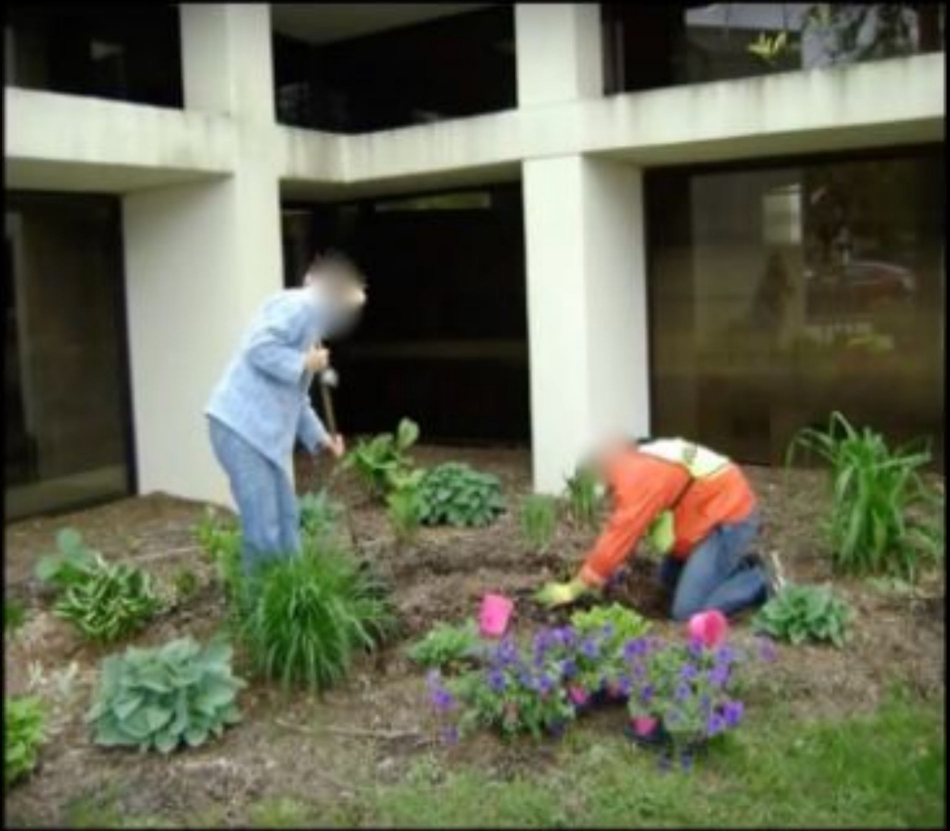

NEIGHBORS

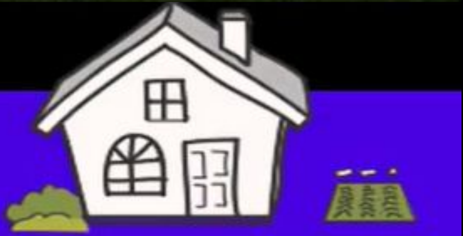

“I enjoy swimming in clean water.”
-Nicole J.

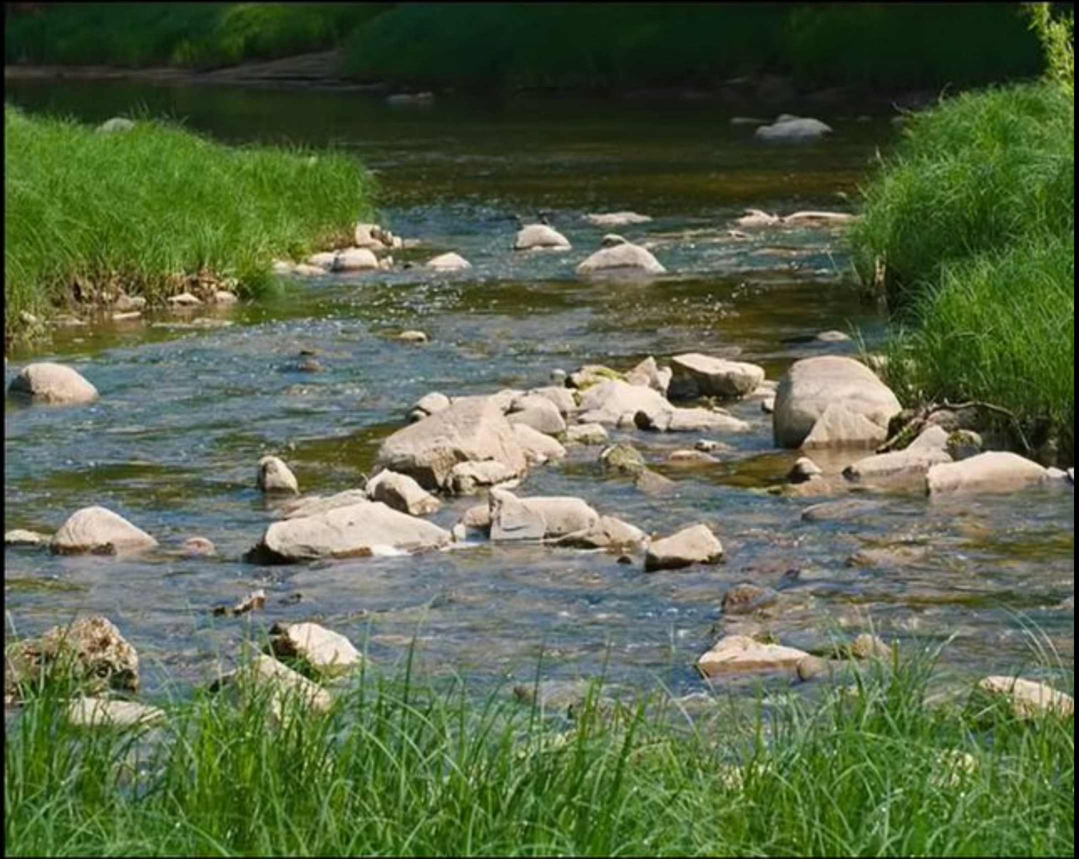

NATURAL WORLD

"My kids like to learn about the environment in school."
-Lori G.

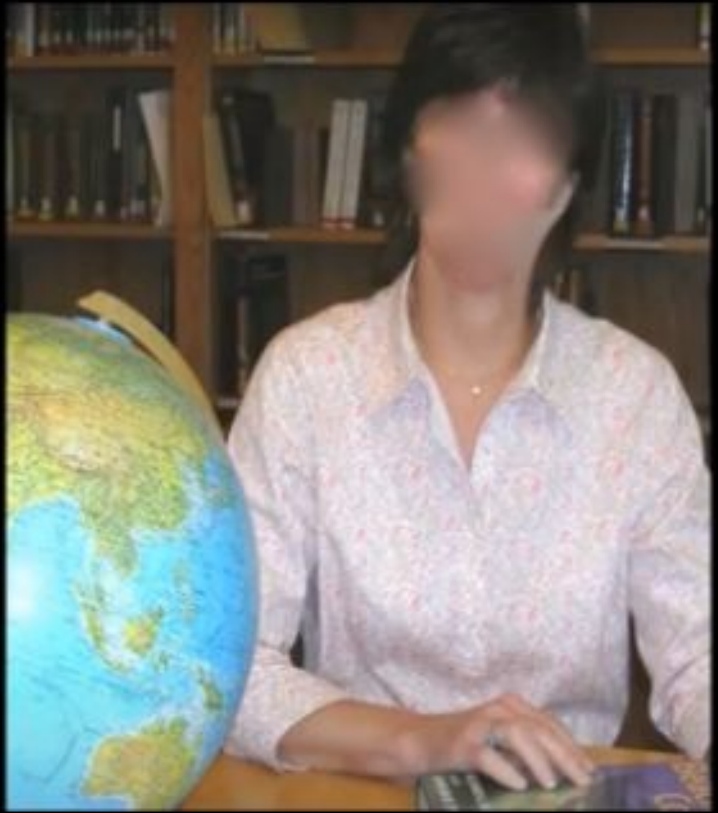

NEIGHBORS

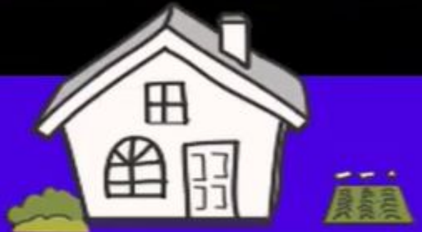

"It's good for my kid's school to use renewable energy."
-Phil M.

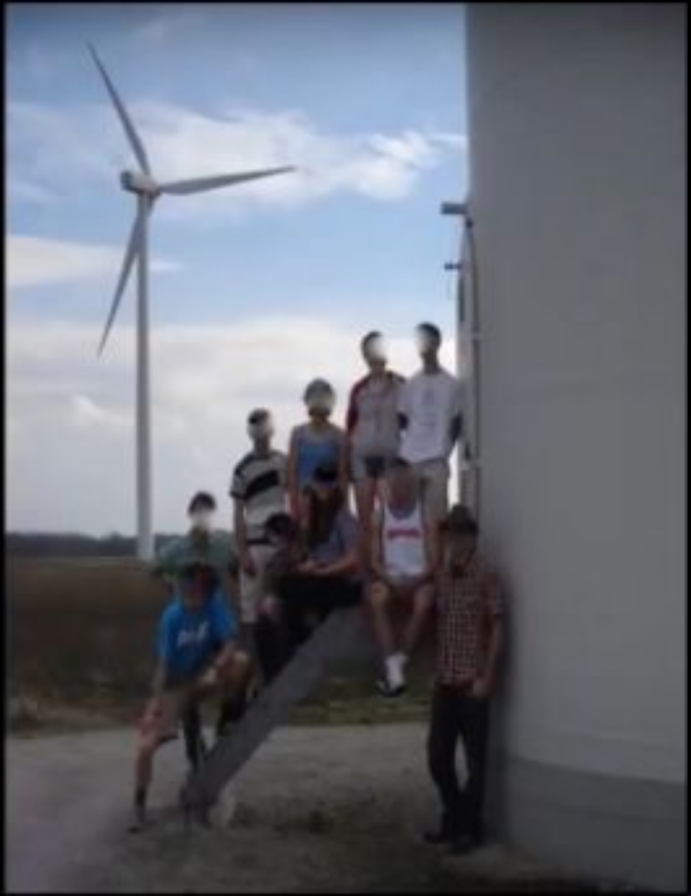

NEIGHBORS

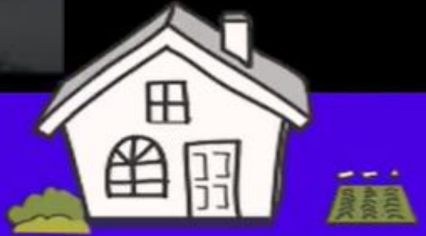

"We should protect the earth because our land is for everyone: humans, plants, and animals."
-Bobby M.

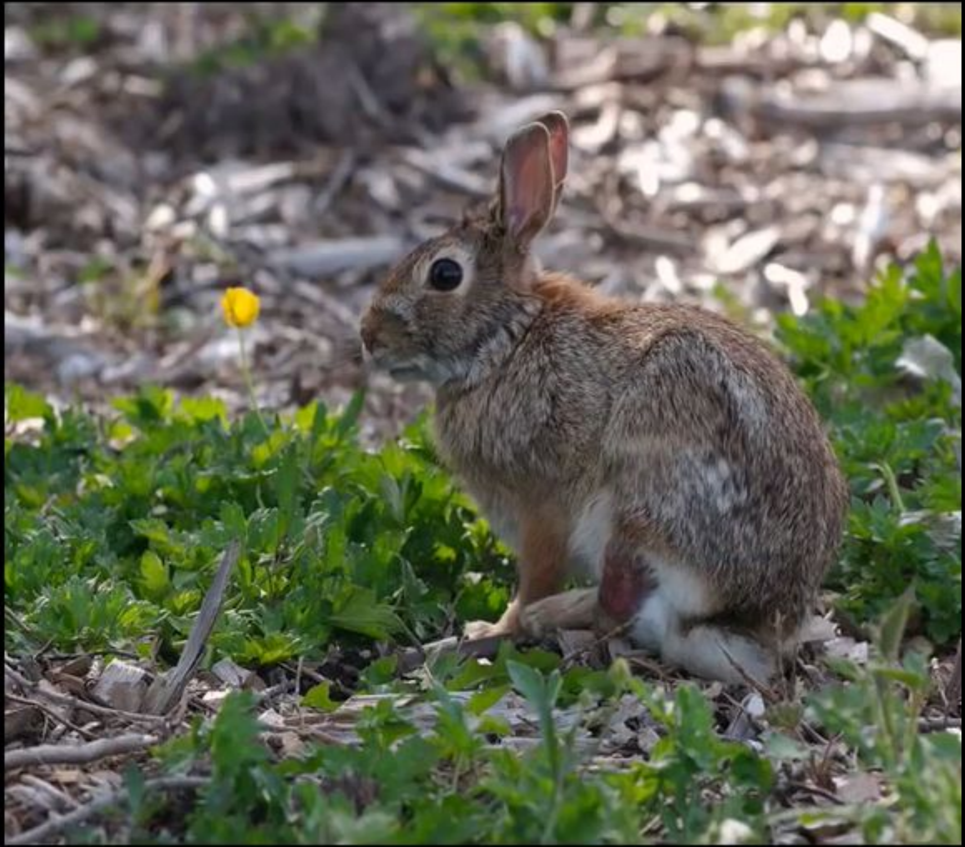

NATURAL WORLD
