# Supplementary material for: Novel approach to delivering pro-environmental messages significantly shifts norms and motivation, but children are not more effective spokespeople than adults
Source: PLoS One. 2021 Sep 8;16(9):e0255457. doi: 10.1371/journal.pone.0255457 (PMC8425541; doi:10.1371/journal.pone.0255457)

“If it’s a nice day, why not be outside”

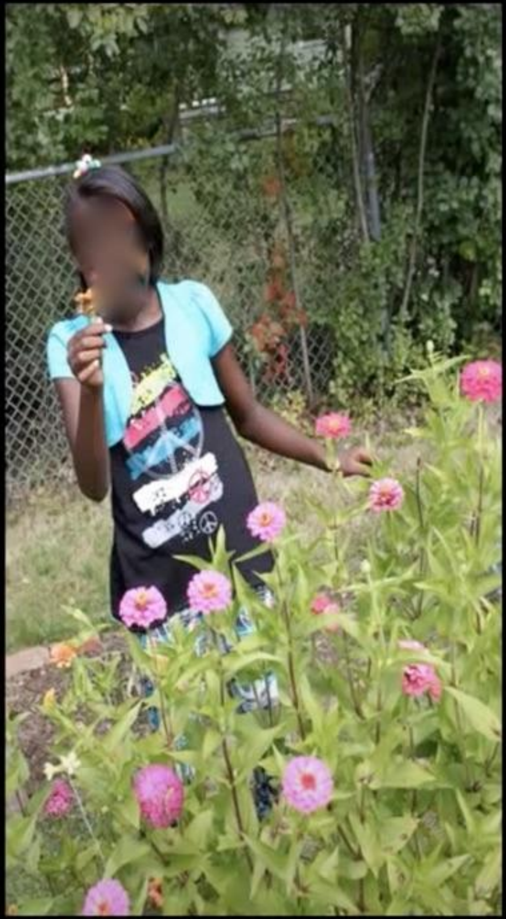

NEIGHBORS

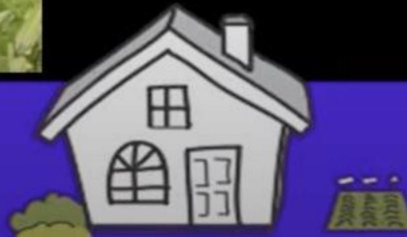

"What we do, even though it seems small, can have a big effect on the world"

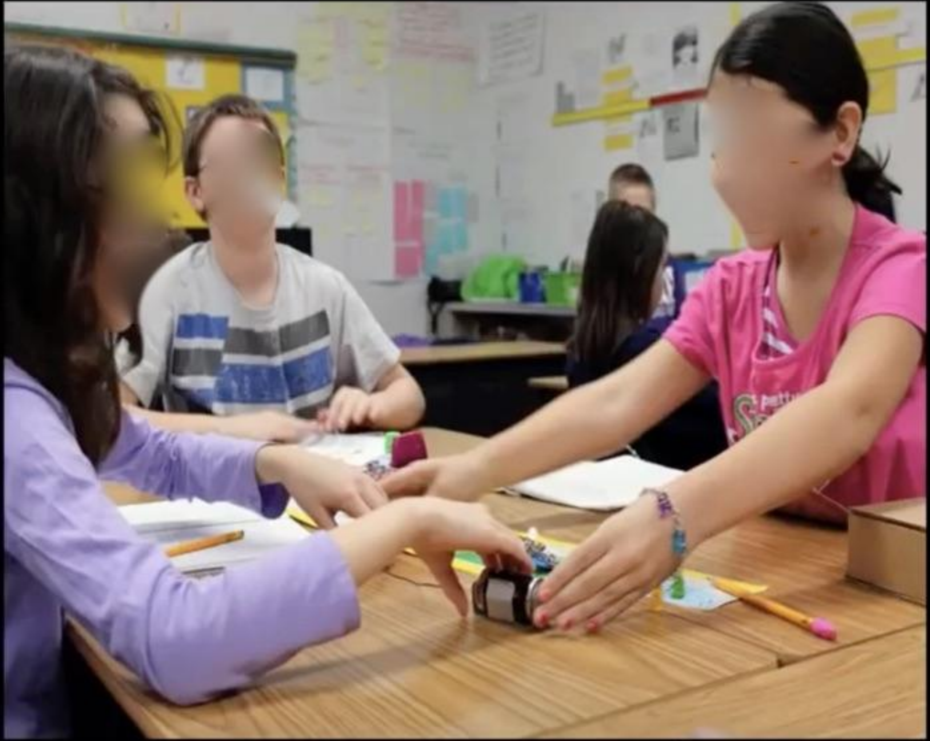

NEIGHBORS

"Saving paper saves trees. Saving trees saves people."

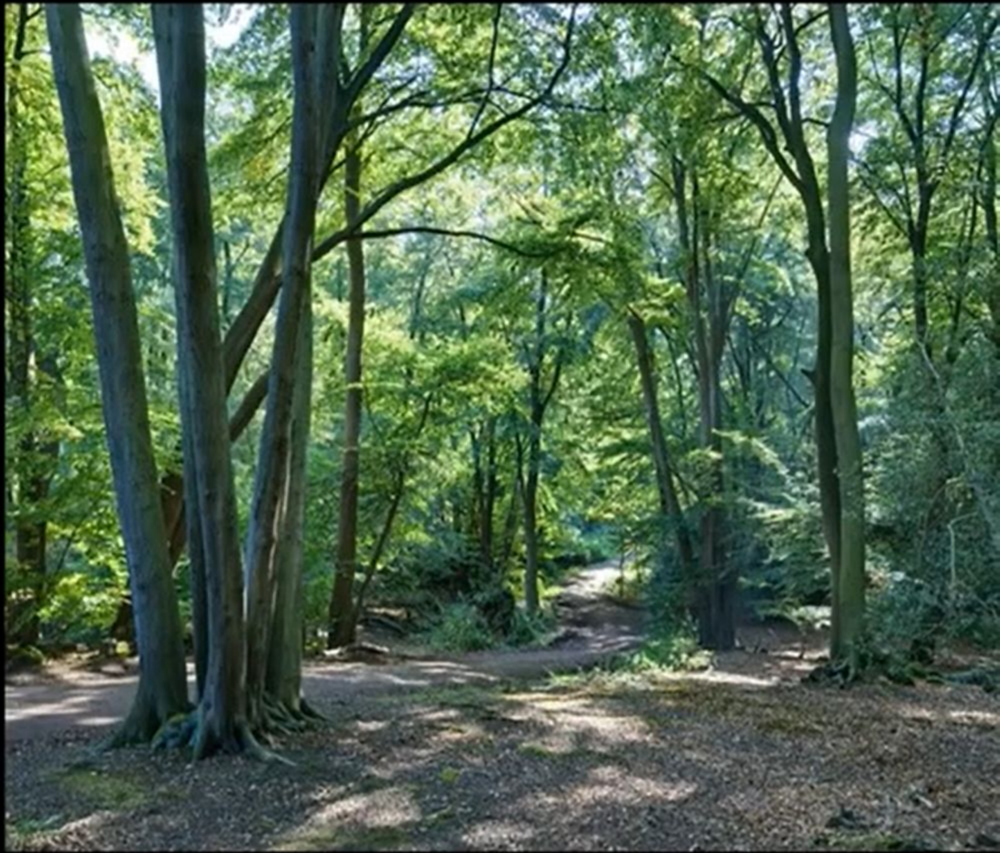

NATURAL WORLD

"If we didn't have the environment, we wouldn't be alive right now"

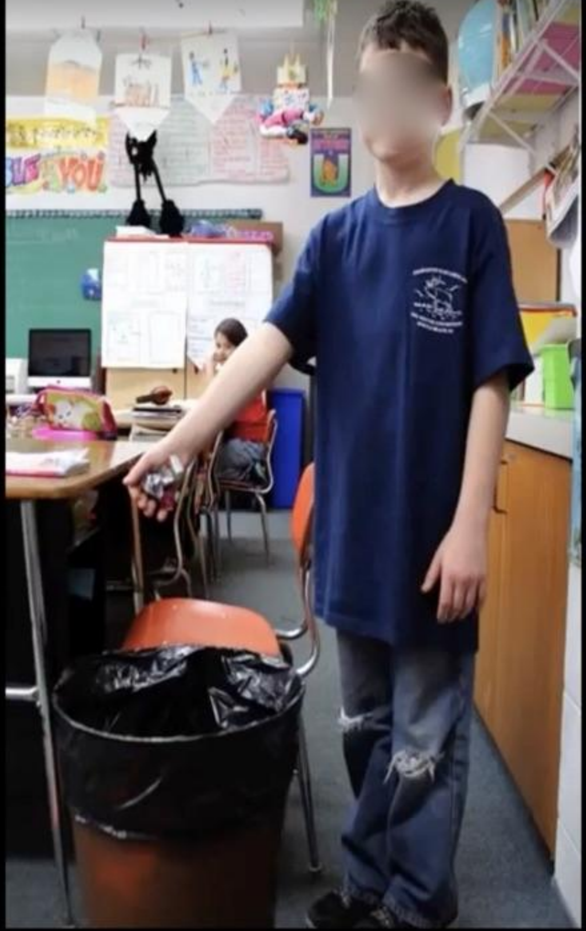

NEIGHBORS

"We have to take care of the environment because this is the only earth we have to live on."

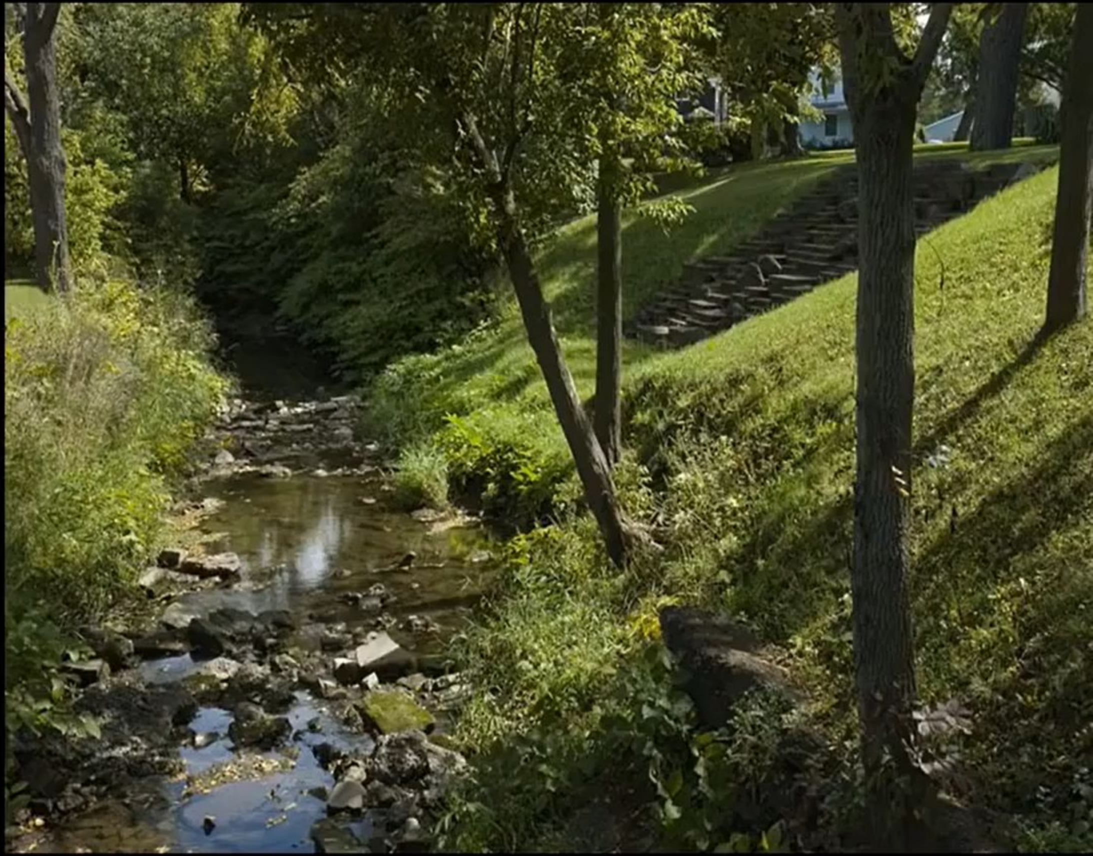

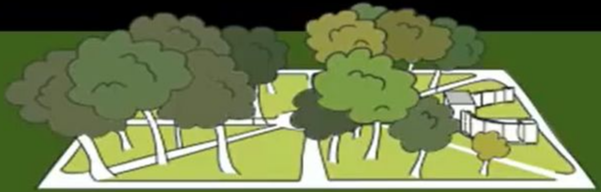

NATURAL WORLD

"Everyone needs to become aware with how we affect our environment."

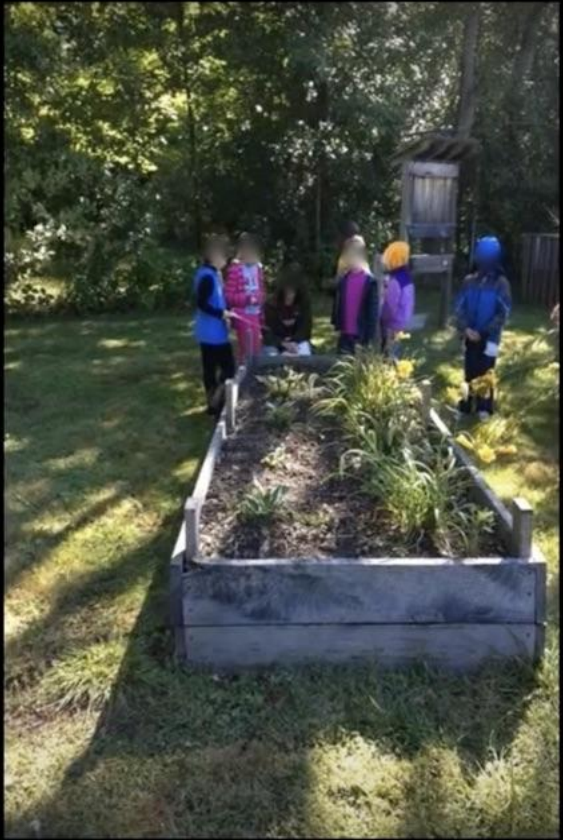

NEIGHBORS

"Sustainability is going back to the old ways...Just simplifying your life"

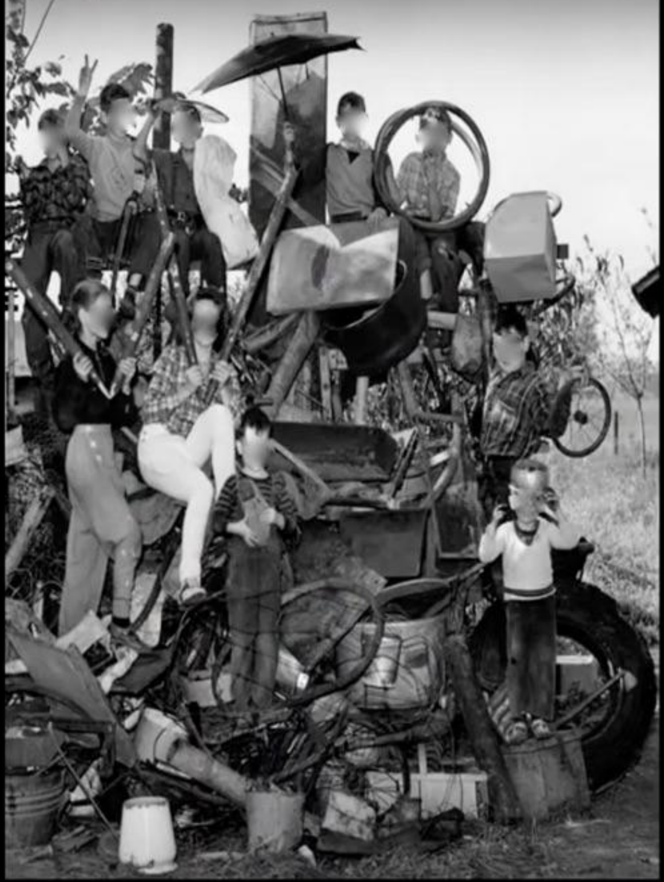

HERITAGE

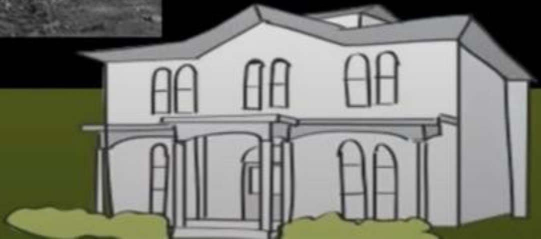

"Our soil is living, it's alive, it's a life. It's our job to be stewards."

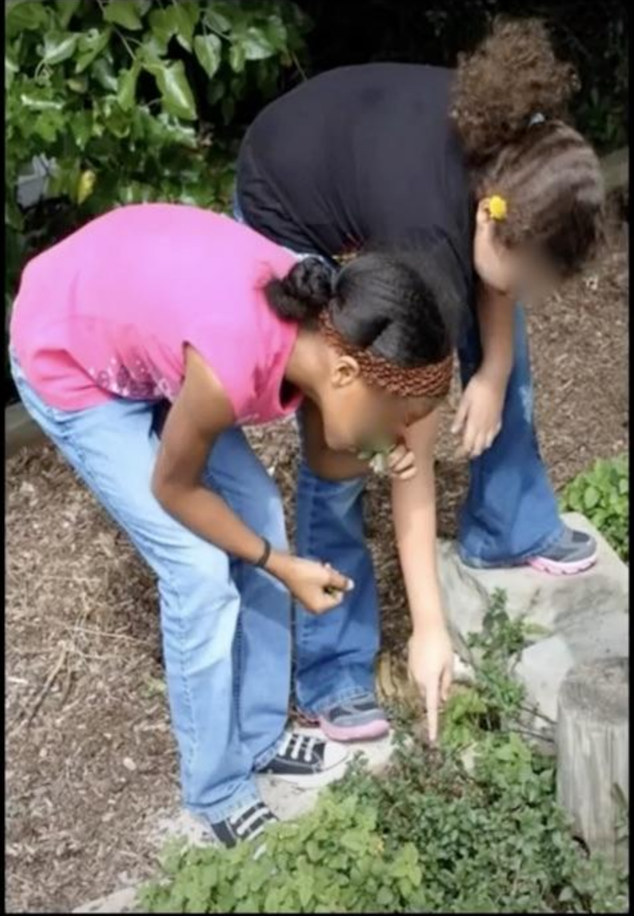

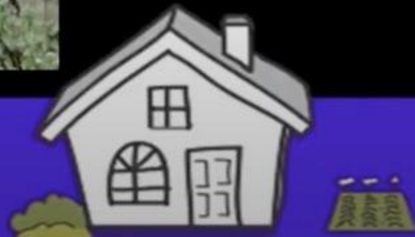

NEIGHBORS

"I always think about how much energy me and my family use. Now it's time to do something about it."

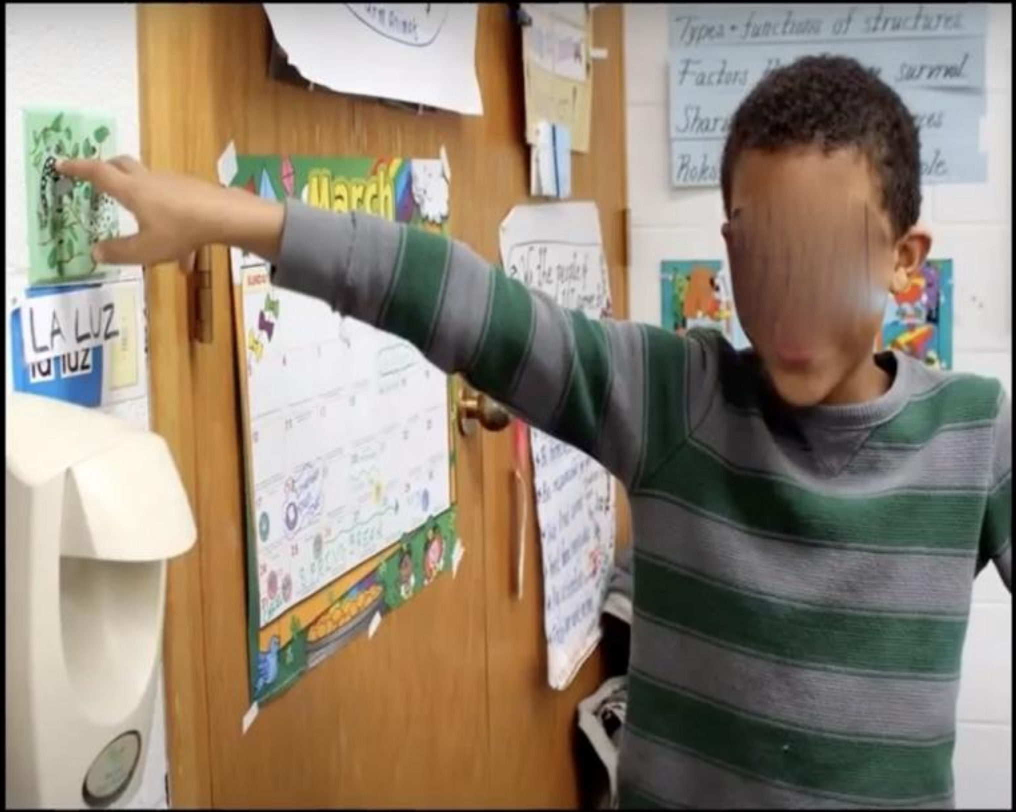

NEIGHBORS

“If we are careful about preserving wetlands, water will be safe for plants and animals”

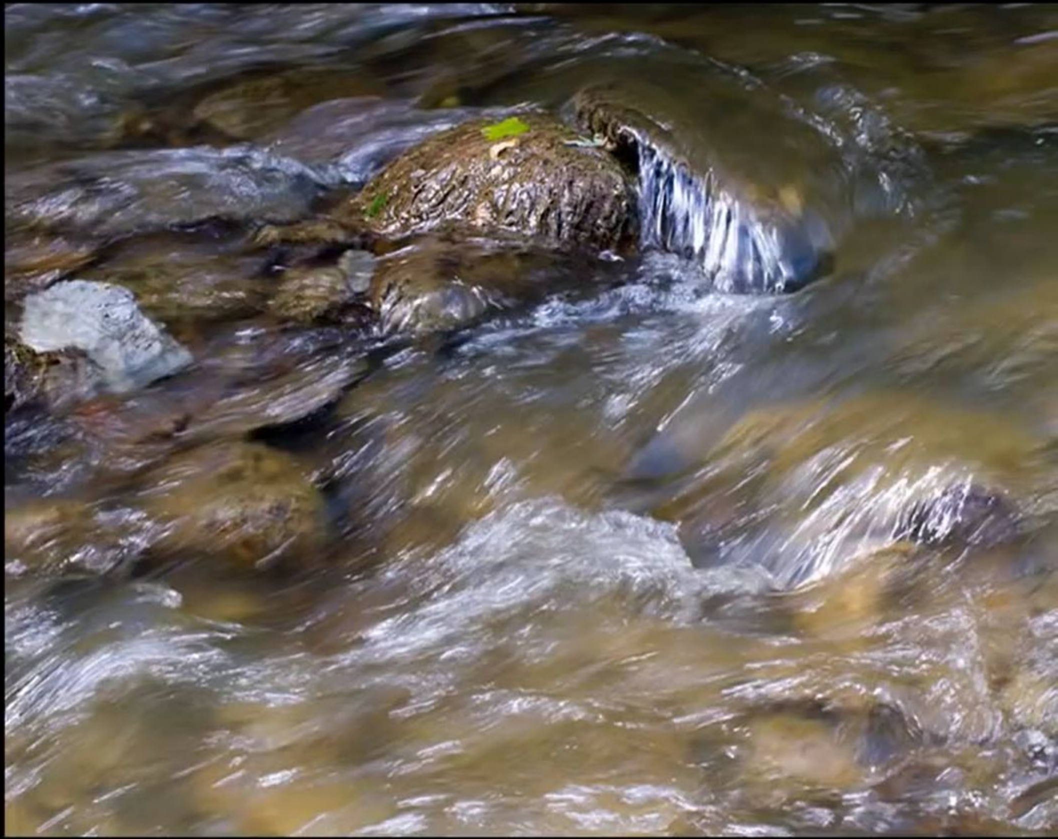

NATURAL WORLD

"Human energy is really important to sustainability."

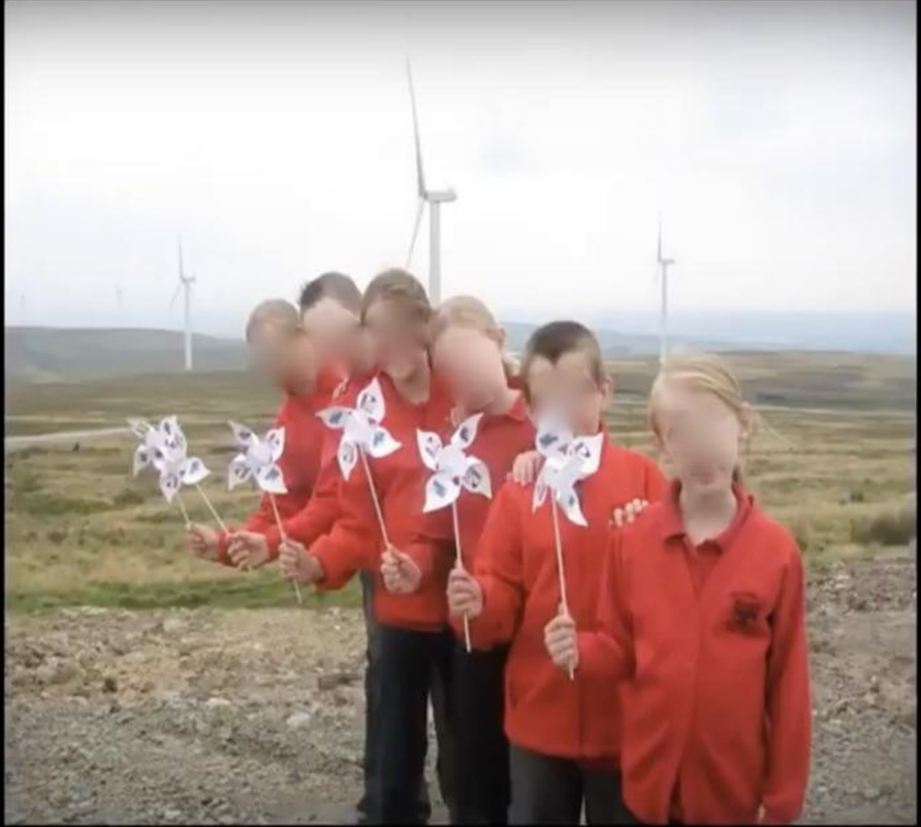

NEIGHBORS

"We should protect the Earth because our land is for everyone: humans, plants, and animals"

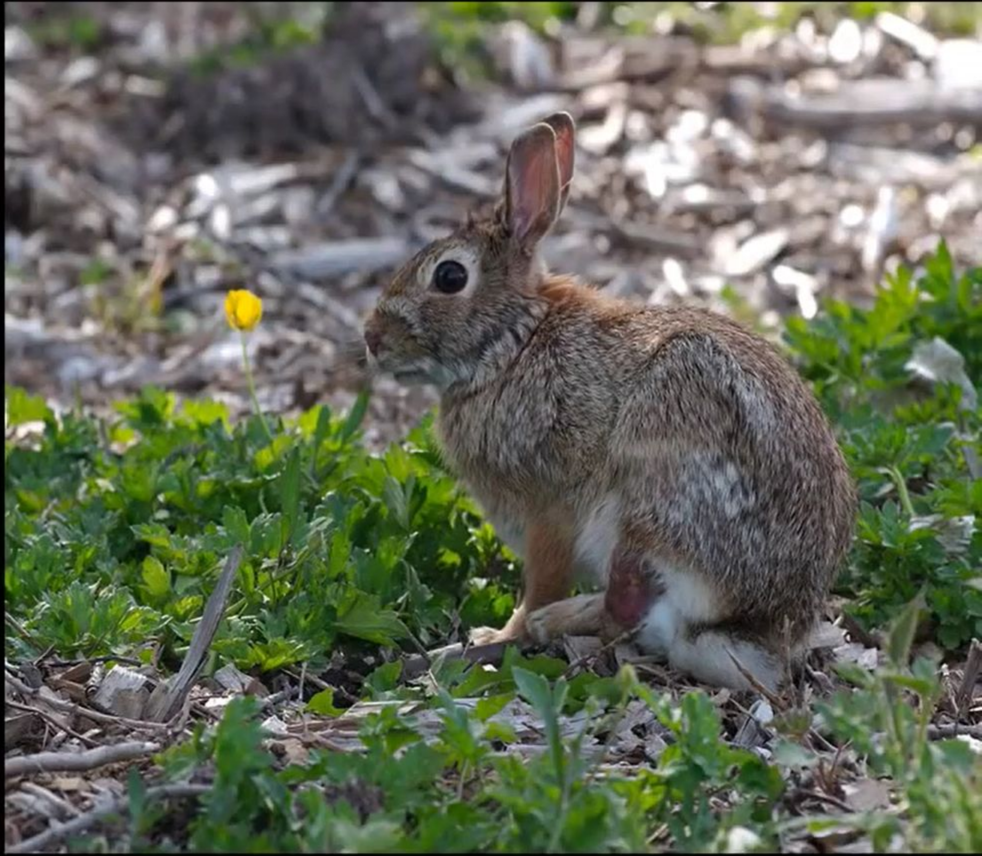

NATURAL WORLD

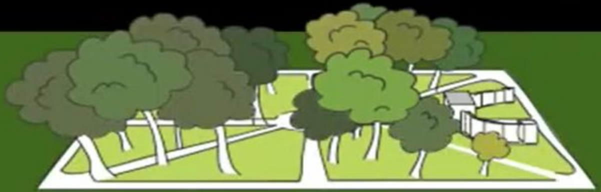

Supplement: S1 Images — (ZIP) [file pone.0255457.s006.zip › Slideshows/Study 3 Slideshow Kids.pdf]
