# Supplementary material for: Novel approach to delivering pro-environmental messages significantly shifts norms and motivation, but children are not more effective spokespeople than adults
Source: PLoS One. 2021 Sep 8;16(9):e0255457. doi: 10.1371/journal.pone.0255457 (PMC8425541; doi:10.1371/journal.pone.0255457)
Supplement: S1 Images — (ZIP) [file pone.0255457.s006.zip › Slideshows/Study 3 Slideshow Adults.pdf]

“When I think of sustainability, I think of being caregivers of our earth, of our planet, of our home.”

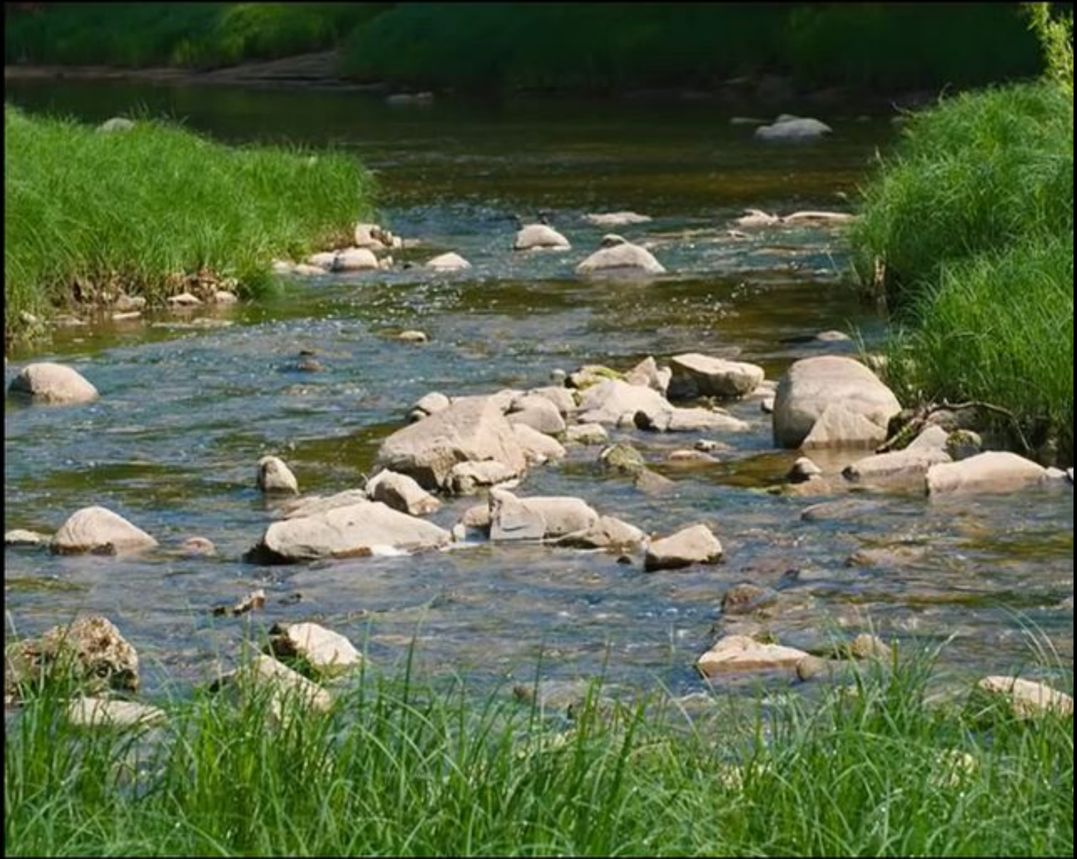

NATURAL WORLD

"If it's a nice day, why not be outside."

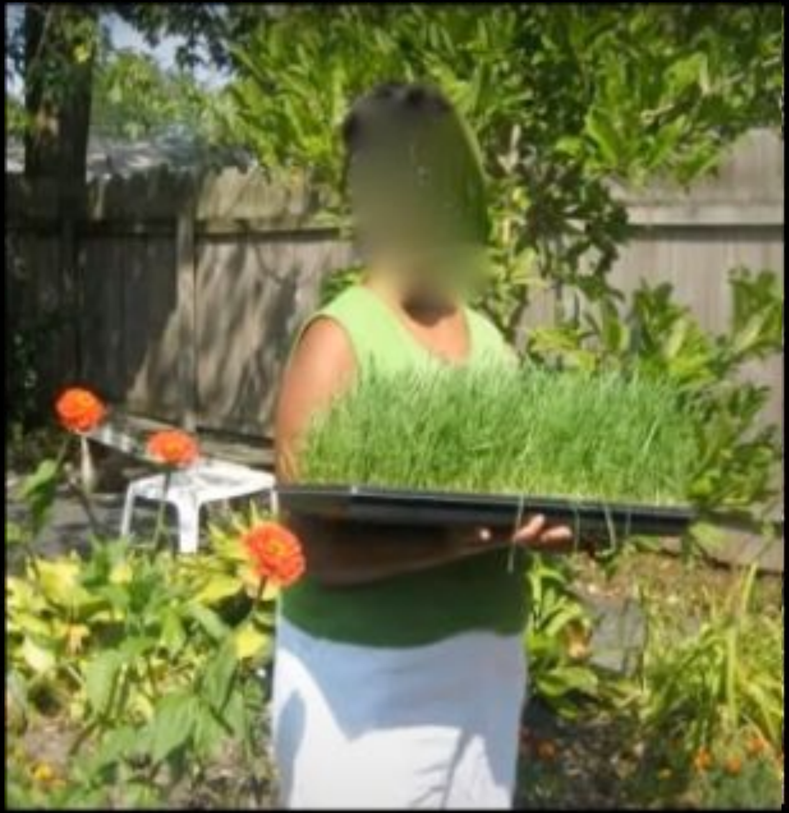

NEIGHBORS

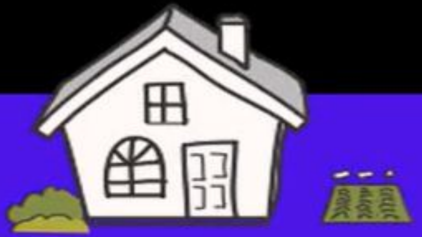

"Animals are relatable and they deserve to have healthy lives just like human beings do."

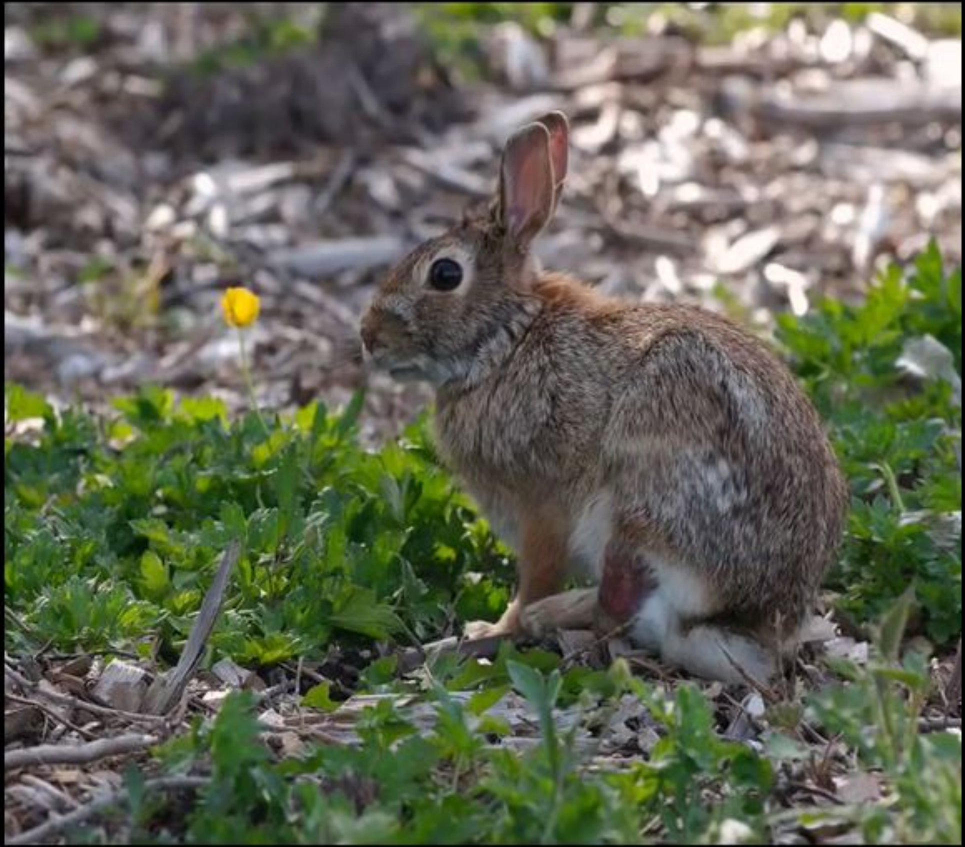

NATURAL WORLD

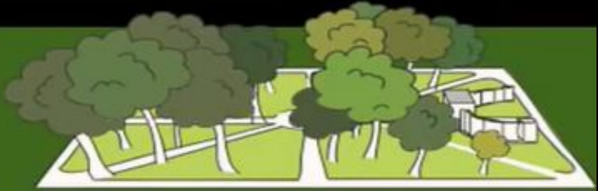

"Everyone needs to become aware with how we affect our environment."

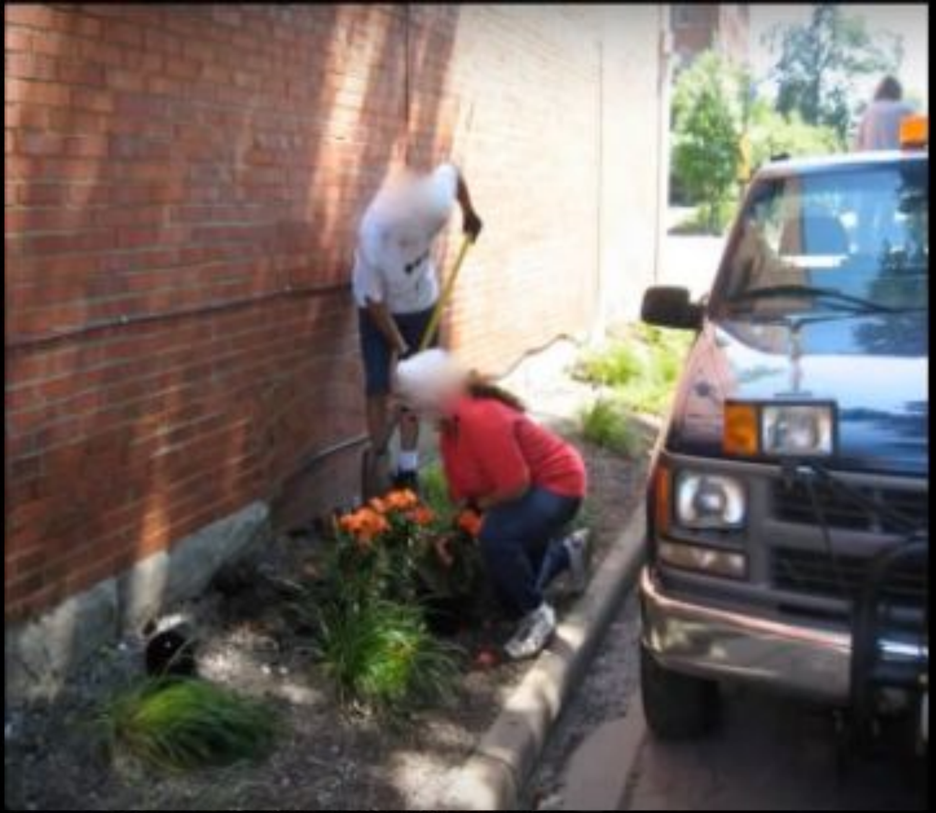

NEIGHBORS

"Saving paper saves trees. Saving trees saves people."

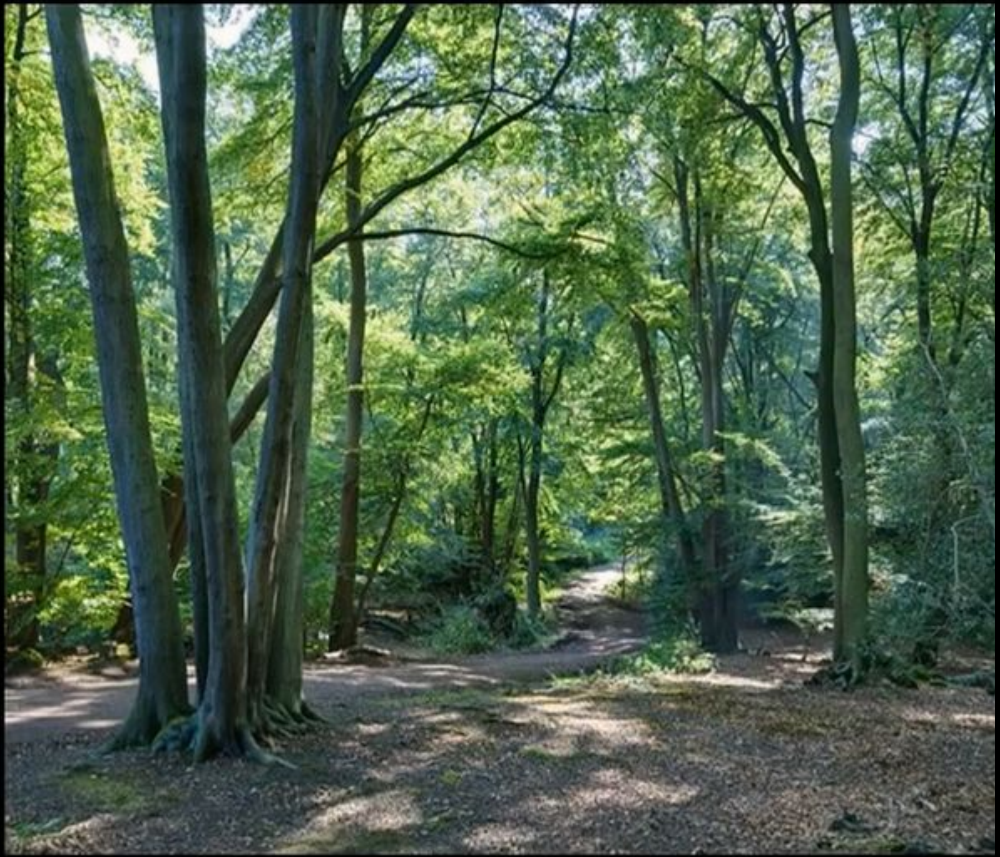

NATURAL WORLD

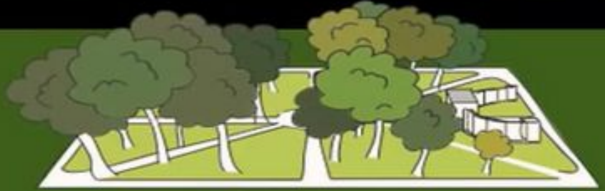

“Our soil is living, it’s alive, it’s a life. It’s our job to be stewards.”

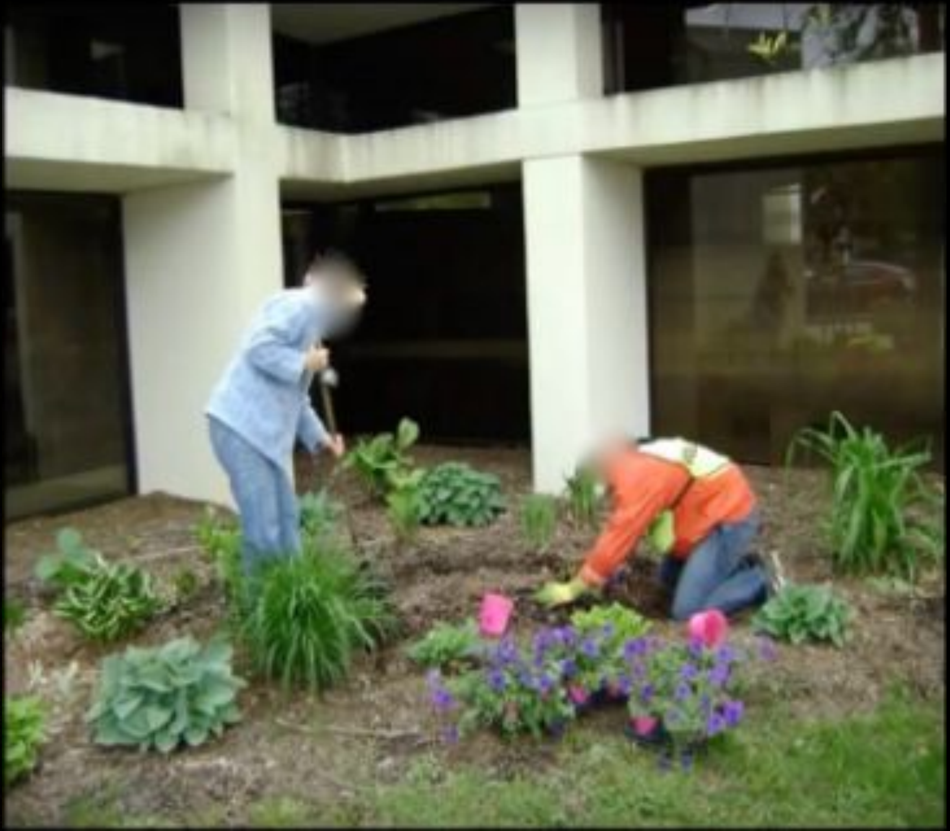

NEIGHBORS

"We have to take care of the environment because this is the only Earth we have to live on."

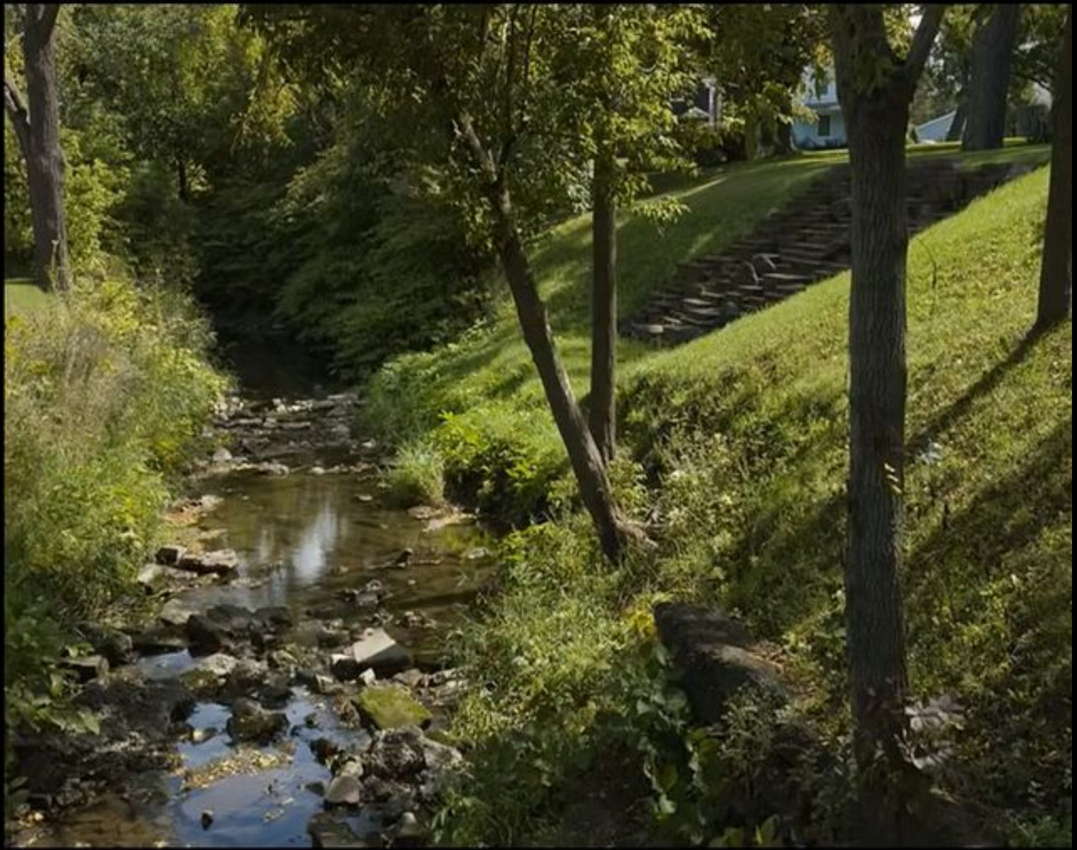

NATURAL WORLD

“Human energy is really important to sustainability.”

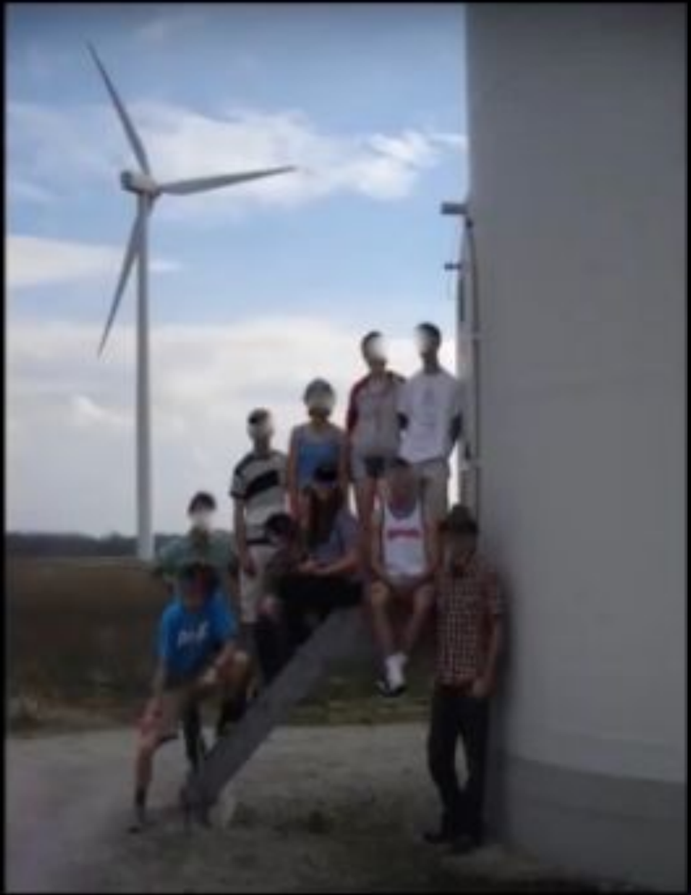

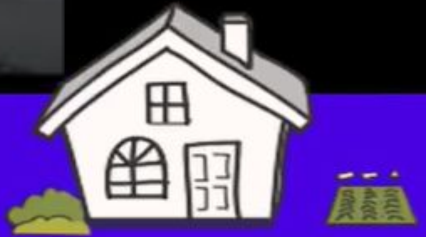

NEIGHBORS

"If we are careful about preserving wetlands, water will be safe for our plants and animals."

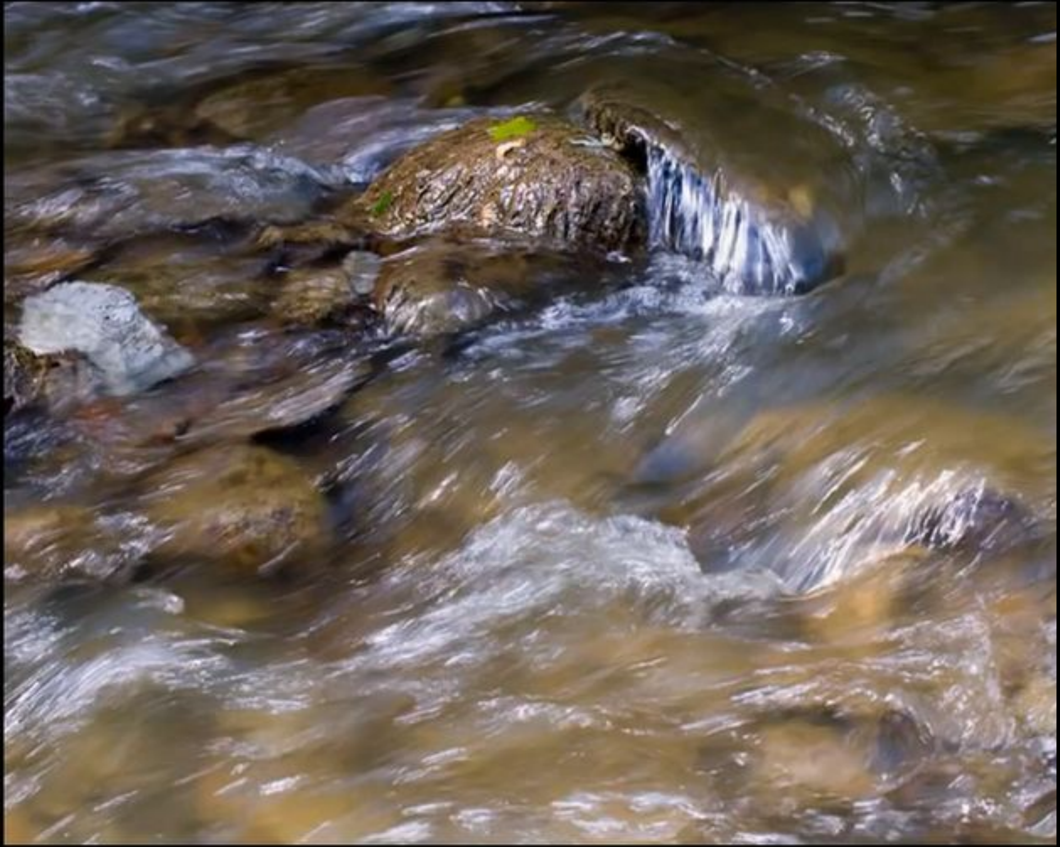

NATURAL WORLD

"I always think about how much energy me and my family use. Now it's time to do something about it."

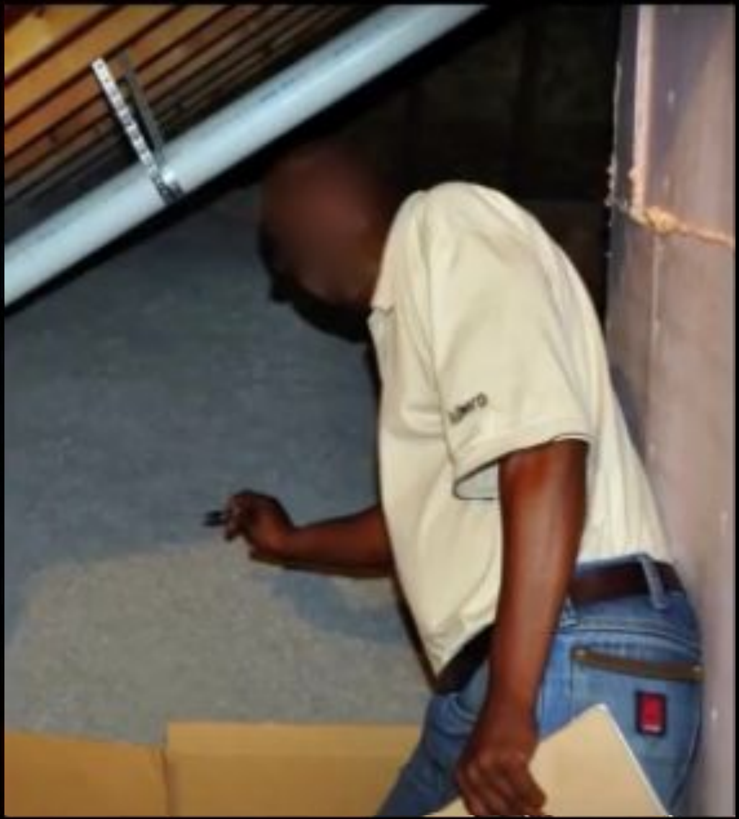

NEIGHBORS

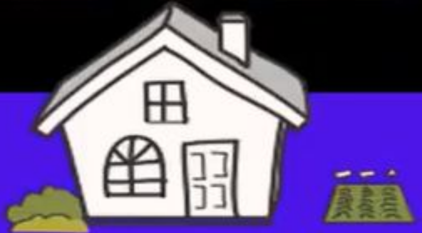

"If we didn't have the environment, we wouldn't be alive right now."

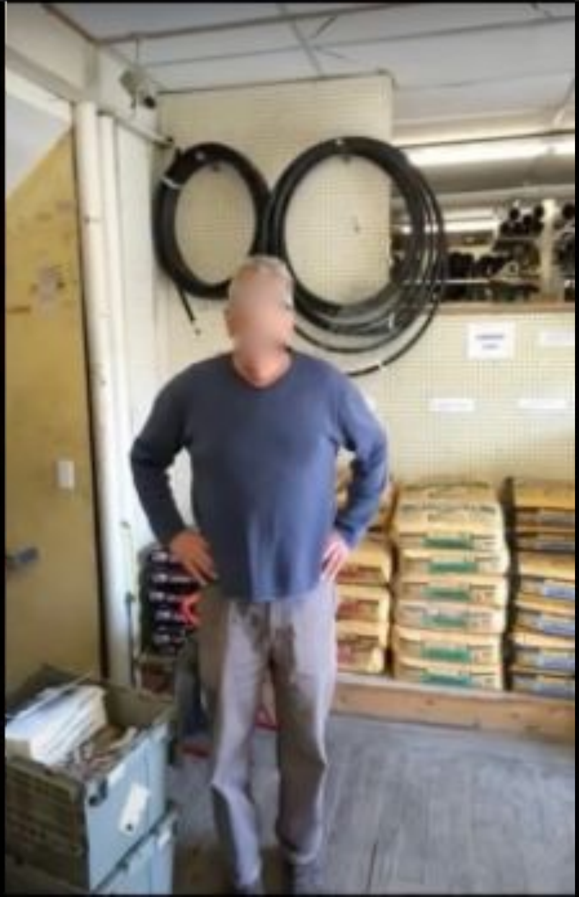

NEIGHBORS

"Sustainability is going back to the old ways… Just simplifying your life."

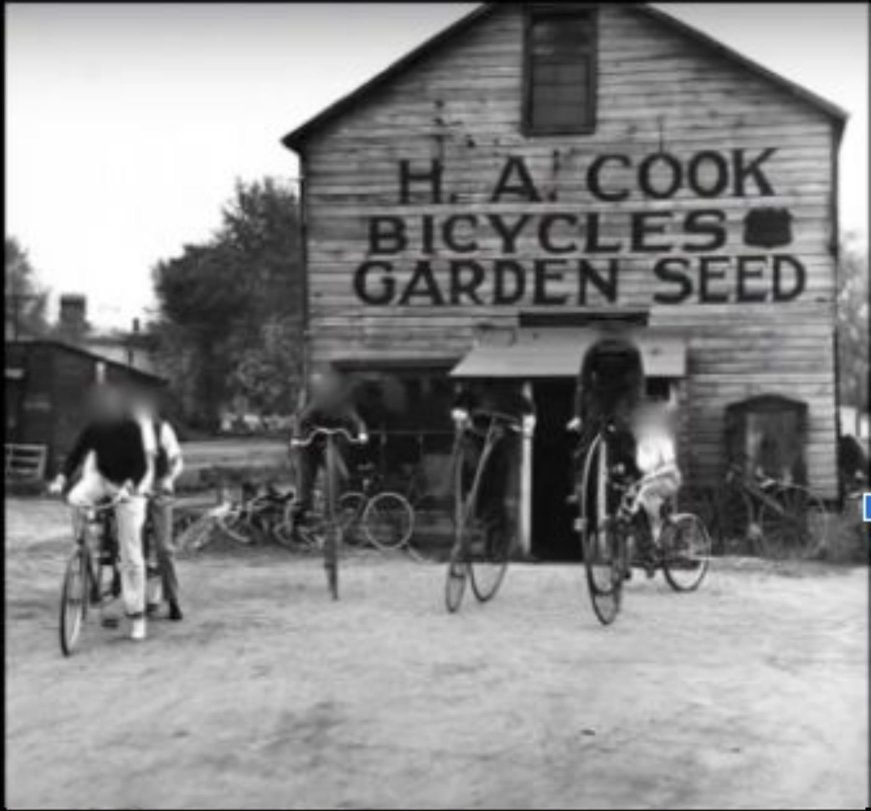

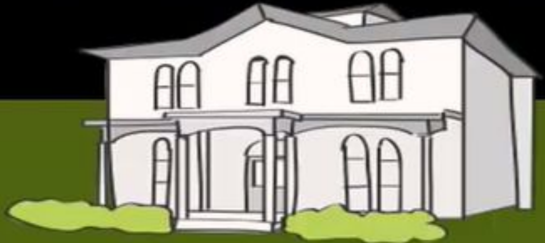

HERITAGE

"What we do, even though it seems small, can have a big effect on the world."

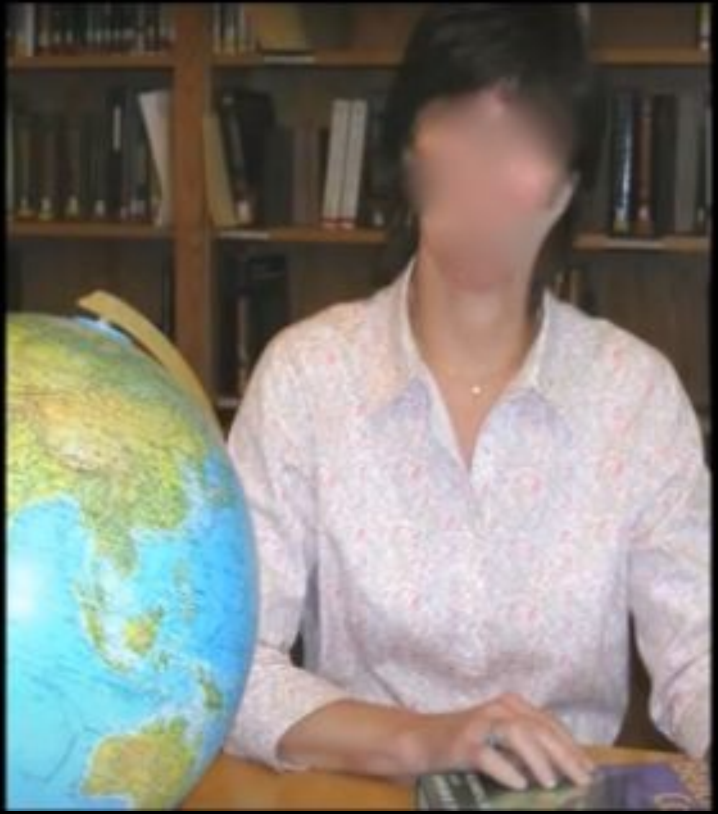

NEIGHBORS
