# Supplementary material for: Novel approach to delivering pro-environmental messages significantly shifts norms and motivation, but children are not more effective spokespeople than adults
Source: PLoS One. 2021 Sep 8;16(9):e0255457. doi: 10.1371/journal.pone.0255457 (PMC8425541; doi:10.1371/journal.pone.0255457)
Supplement: S1 Images — (ZIP) [file pone.0255457.s006.zip › Slideshows/Study 1 Slideshow Kids.pdf]

"You should use less energy to help fight climate change."
-Ashley S. 5th grade

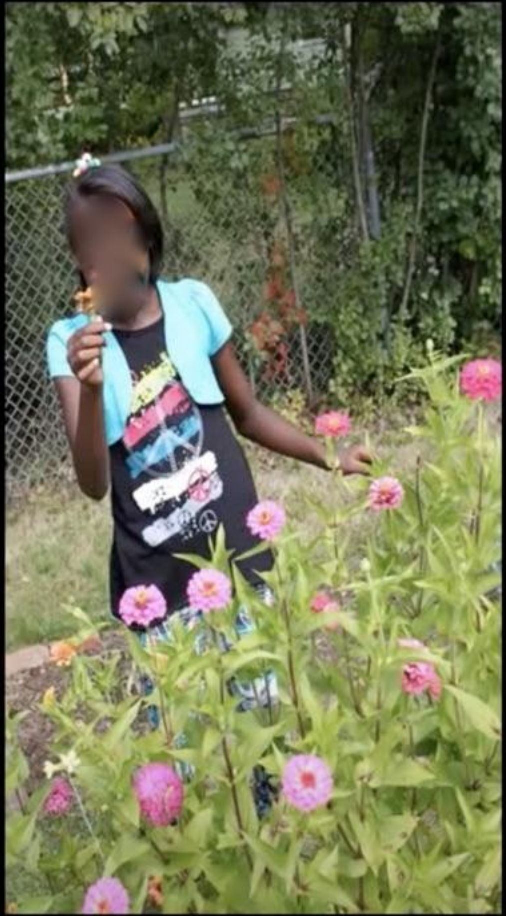

NEIGHBORS

"You need to think about how the planet will be for kids in the future."

-Madeline L. 4th grade

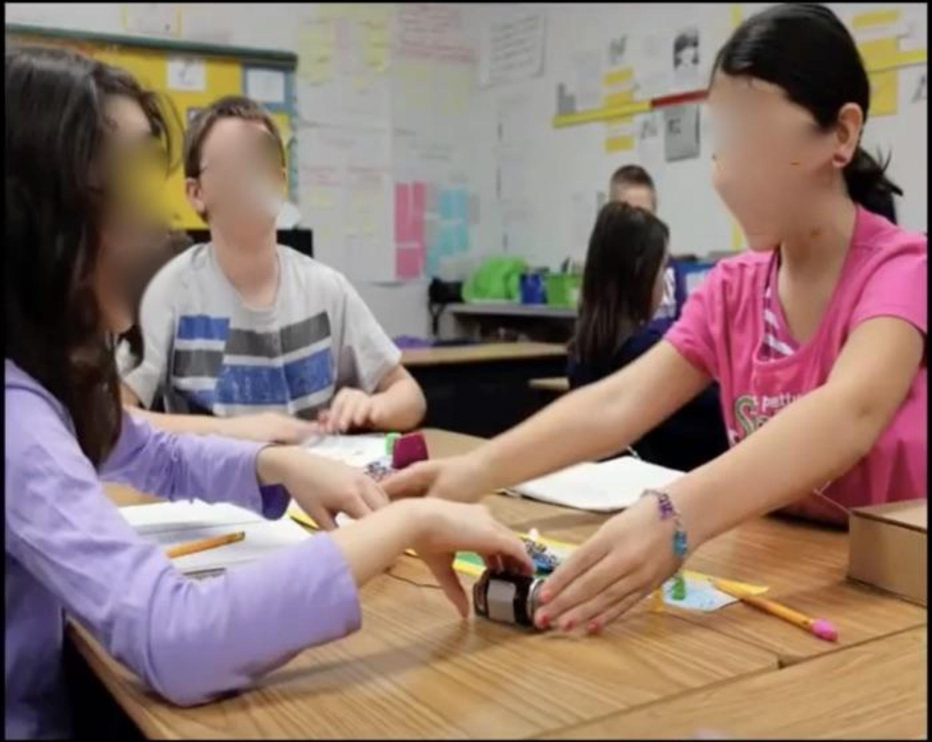

NEIGHBORS

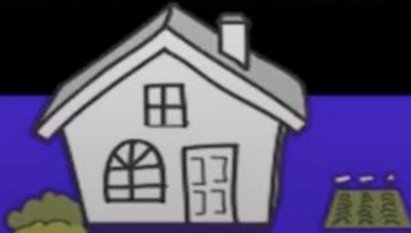

“You should do something to protect the forests.“
-Annie B. 5th grade

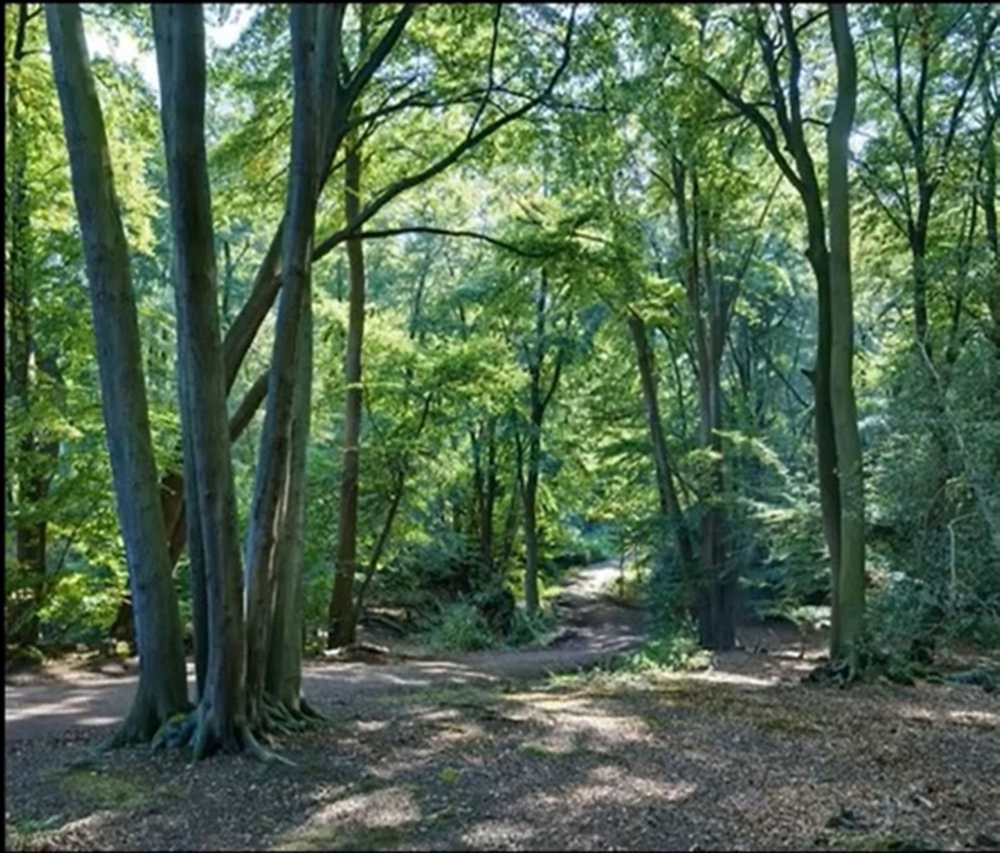

NATURAL WORLD

"You should make changes in your life that help us waste less."

-Justin B. 4th grade

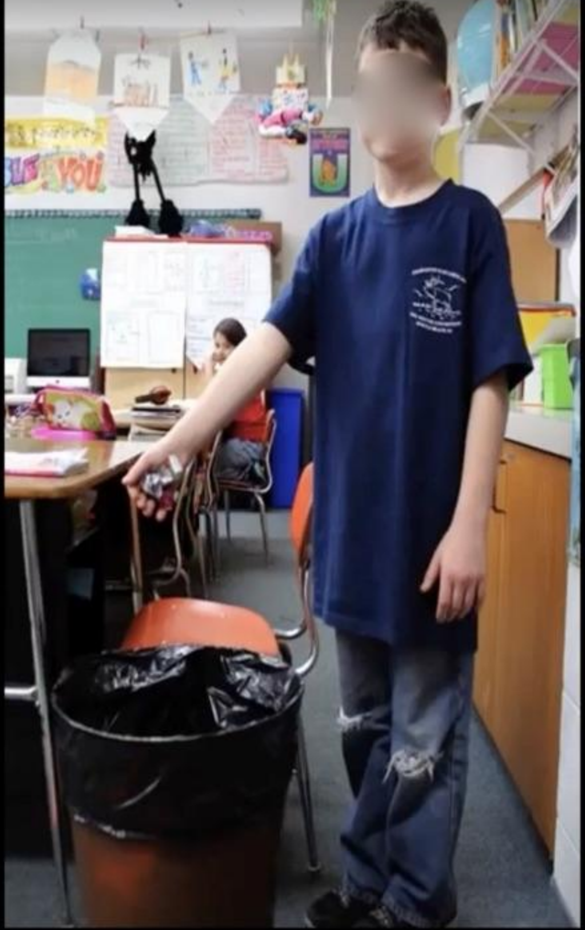

NEIGHBORS

"I think businesses need to be polluting less."

-Elizabeth S. 4th grade

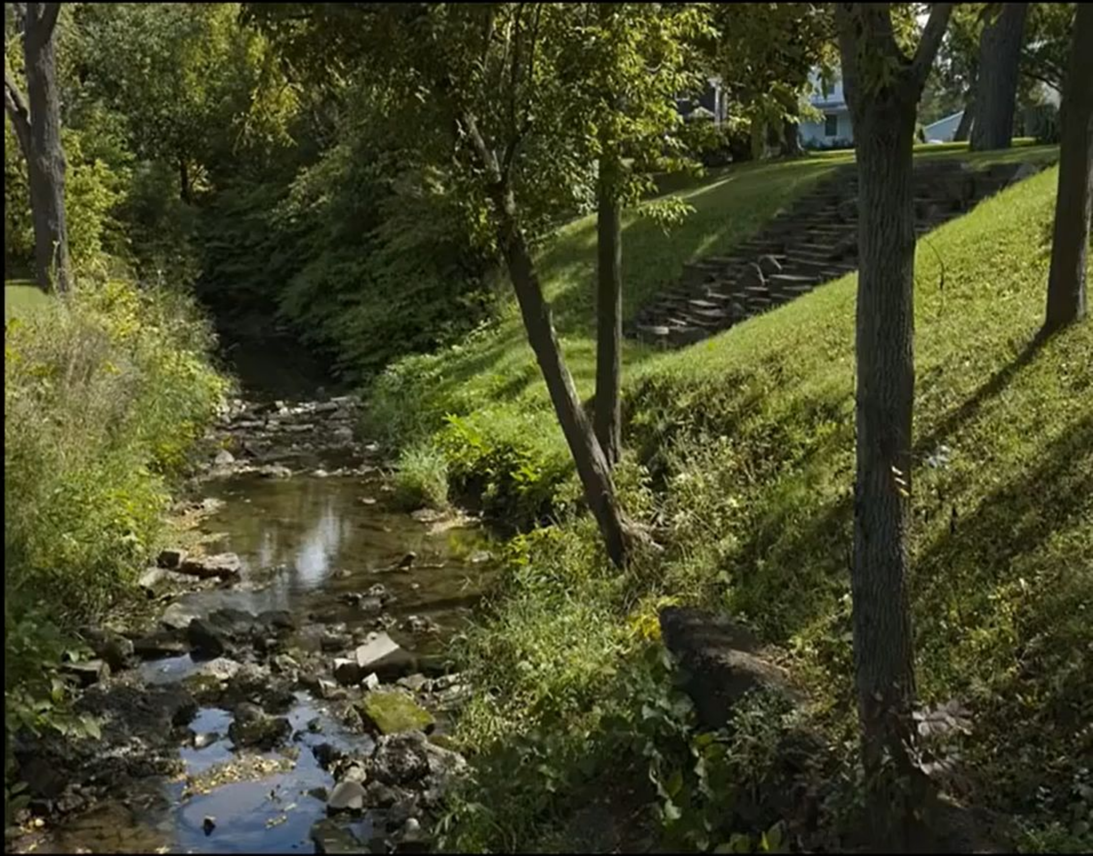

NATURAL WORLD

"You should garden in your yard so you can have fresh food."
-Ashley W. 3rd grade

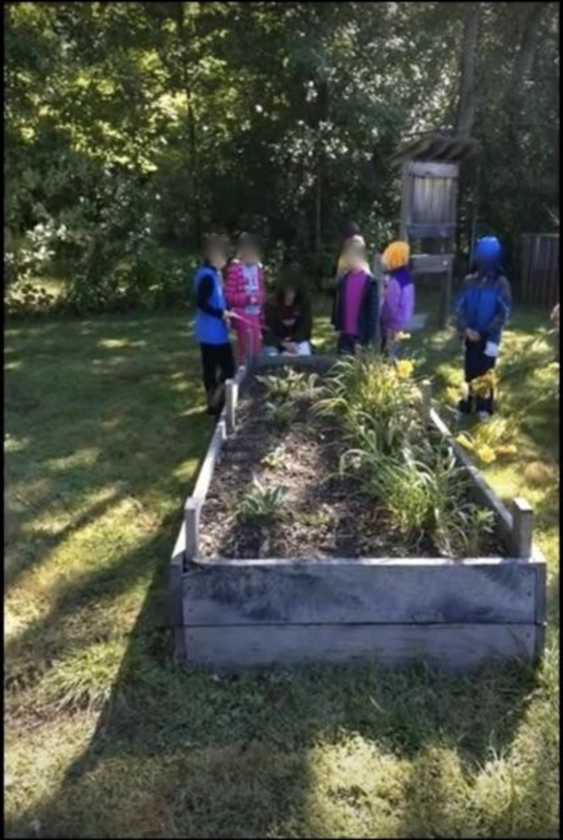

NEIGHBORS

"You have to do more to stop hurting the environment."
-George H. 4th grade

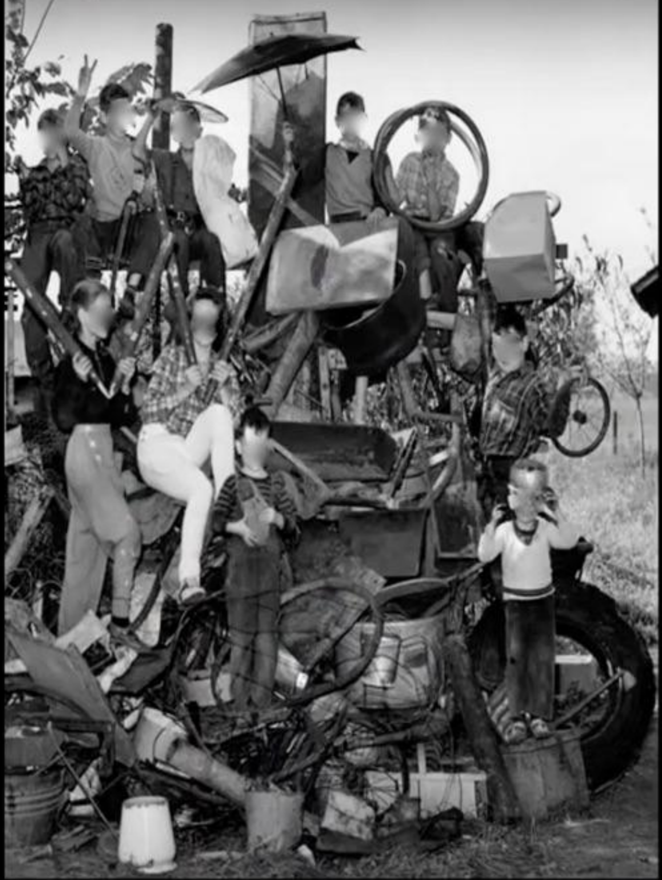

"You should work to help our community and environment."
-Anna C. 3rd grade

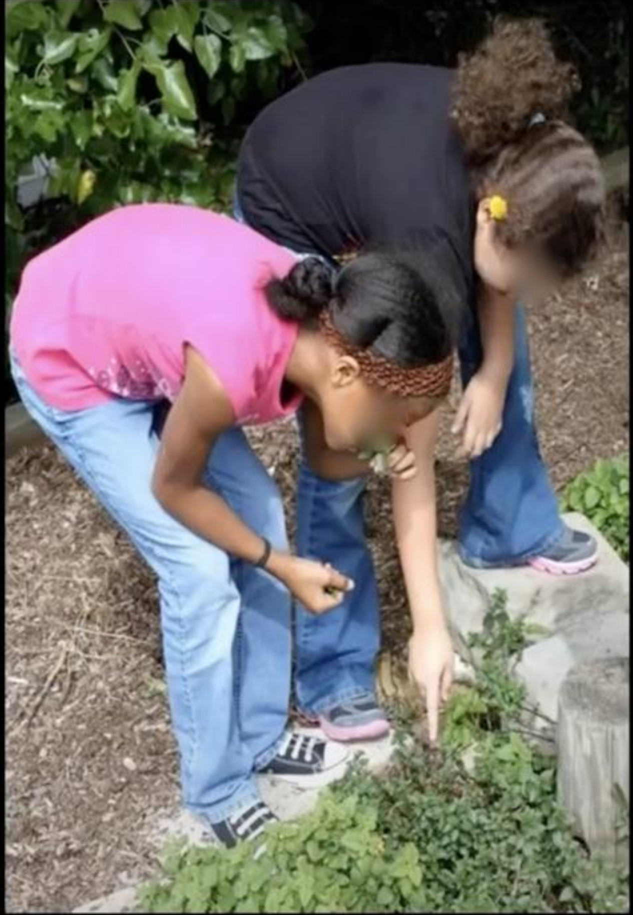

NEIGHBORS

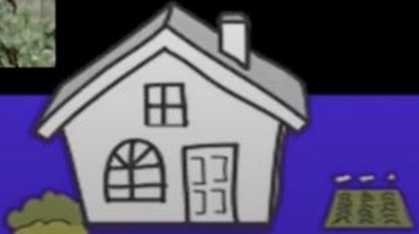

"People who use less water should be rewarded."
-Jack H. 5th grade

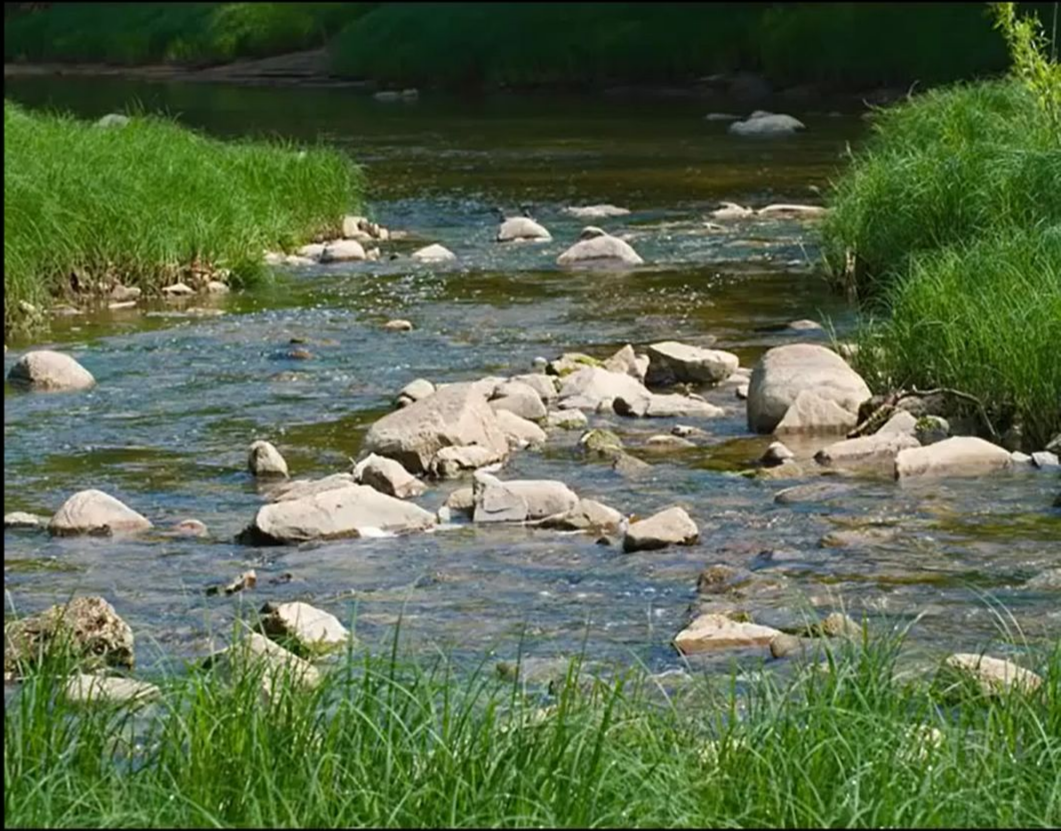

NATURAL WORLD

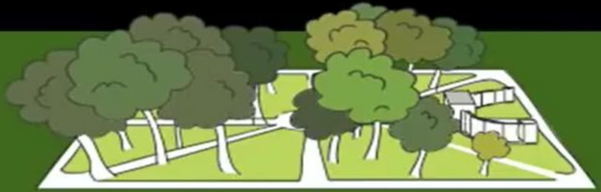

“You should care more about your electricity use.“

-Josh K. 5th grade

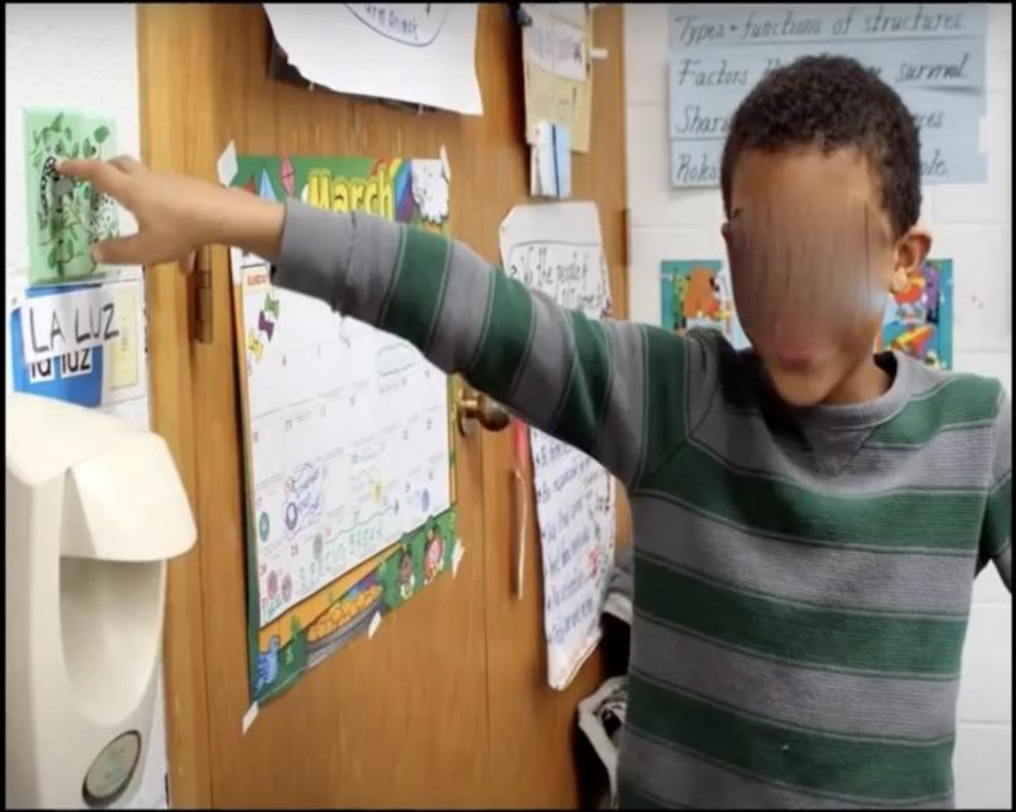

NEIGHBORS

“You’ve got to work towards having cleaner air.“
-Laura H. 6th grade

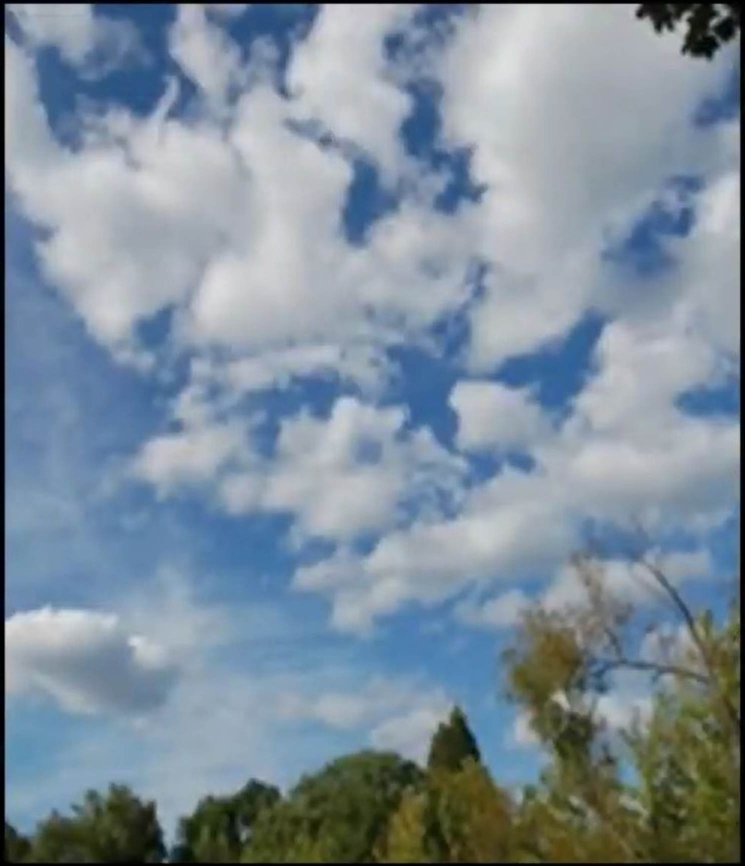

NATURAL WORLD

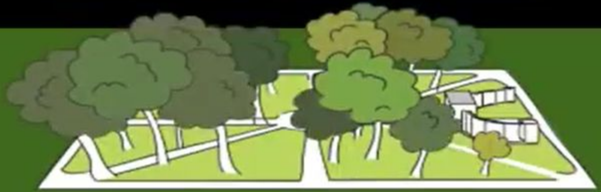

"You should ride your bike more often."
-Jeremy C. 3rd grade

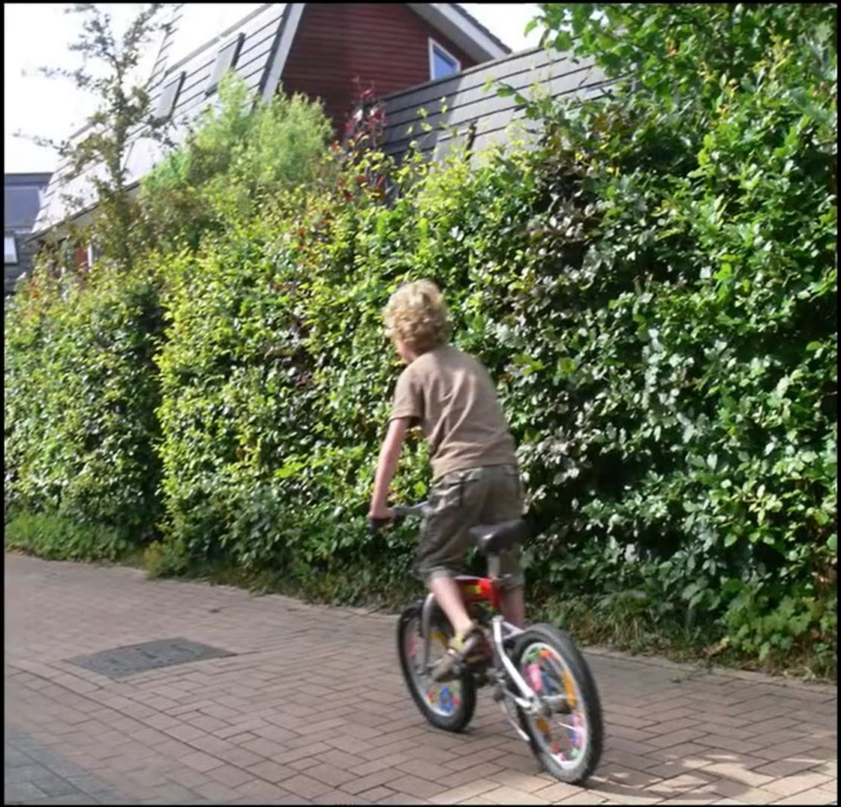

NEIGHBORS

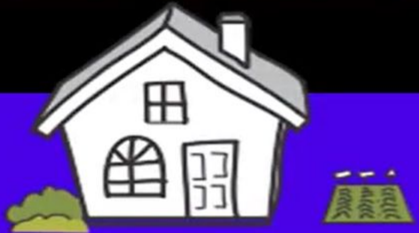

“You need to make sure not to pollute the water.“
-Nicole J. 3rd grade

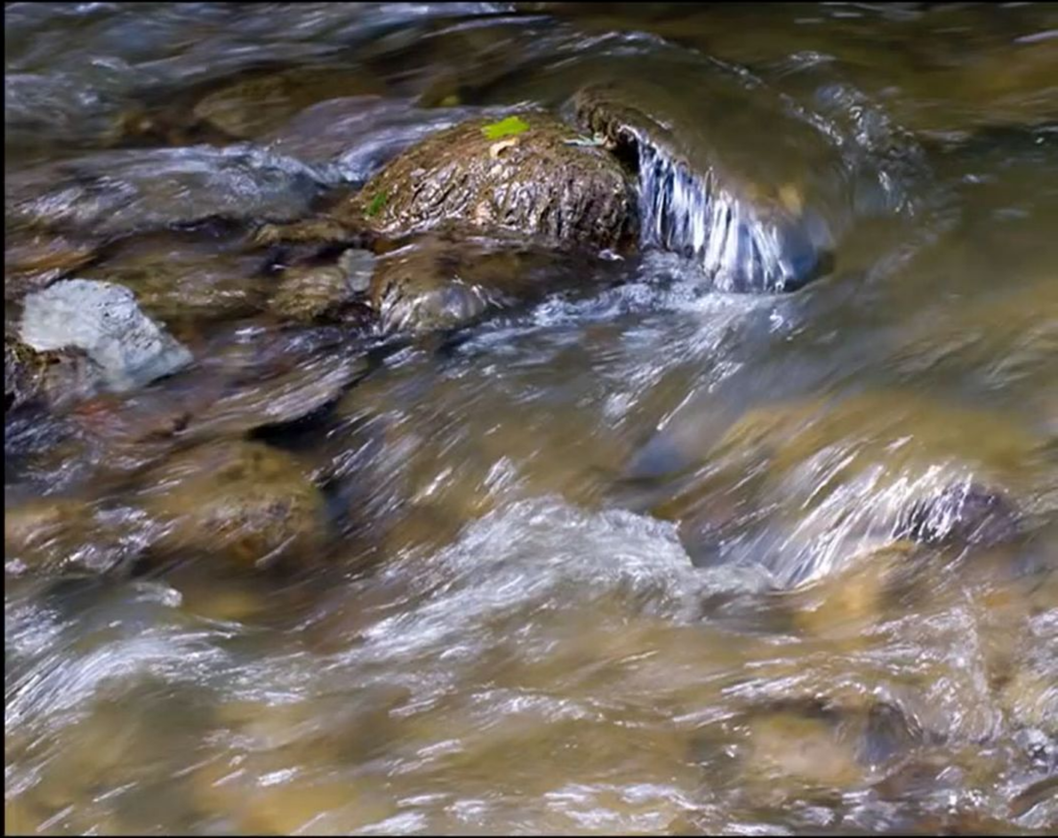

NATURAL WORLD

“You should help protect endangered animals.“
-Lori G. 3rd grade

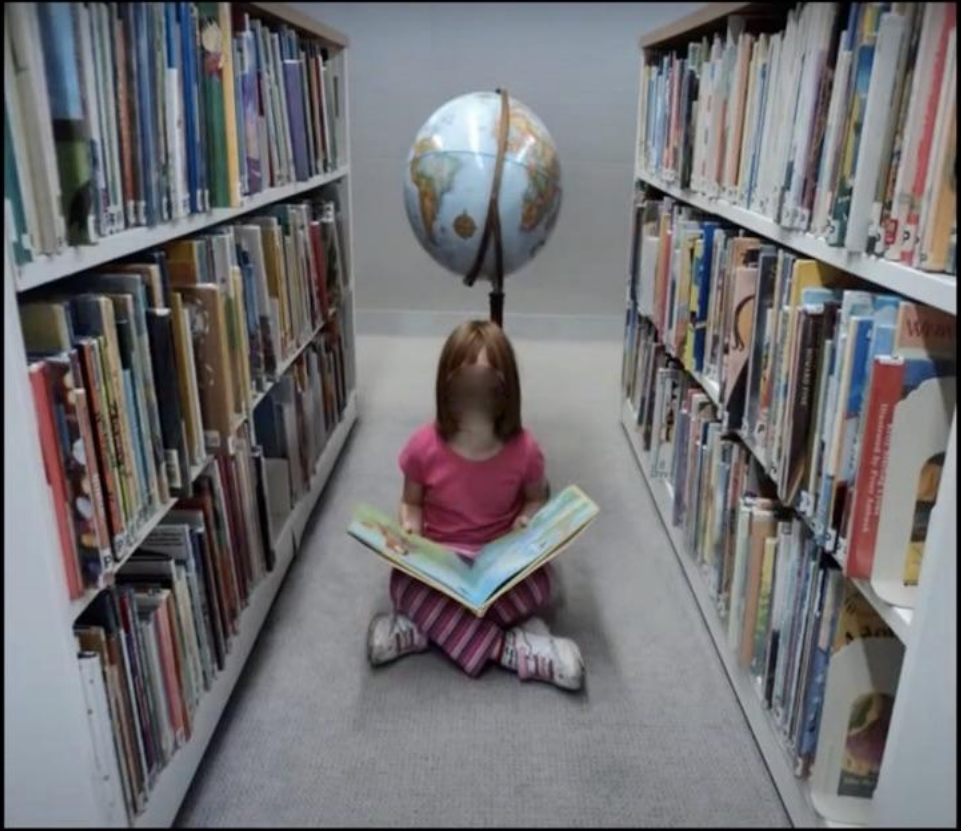

NEIGHBORS

“You should be using more renewable energy.“

-Phil M. 4th grade

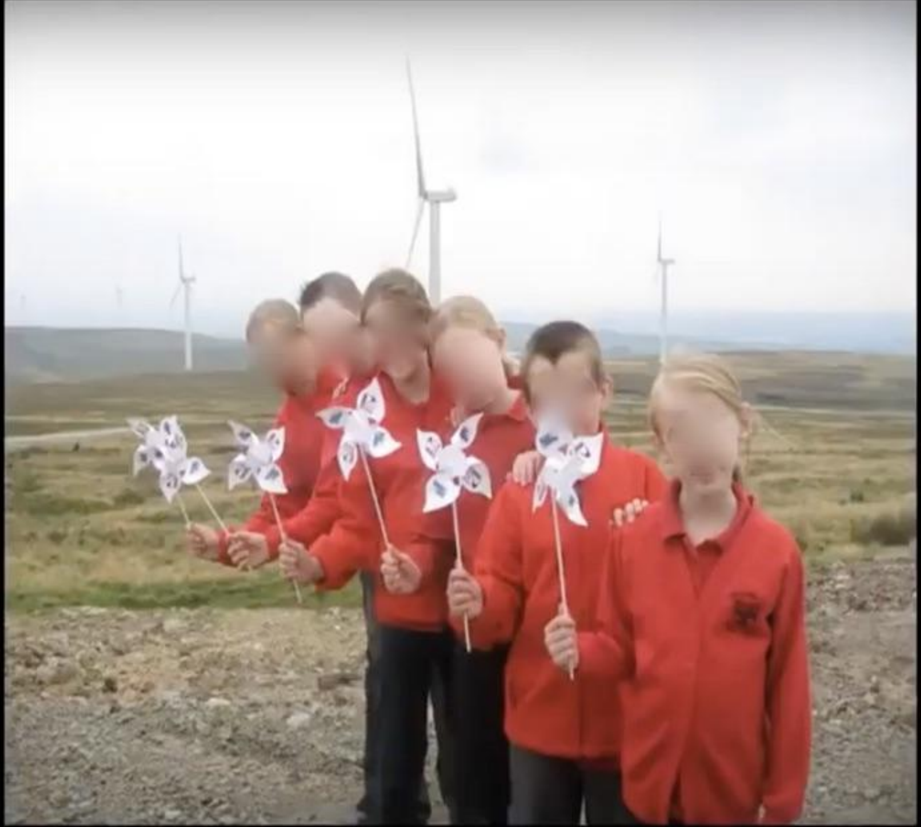

NEIGHBORS

"Our land is for everyone: humans, plants, and animals. You should be sharing it.“

-Bobby M. 5th grade

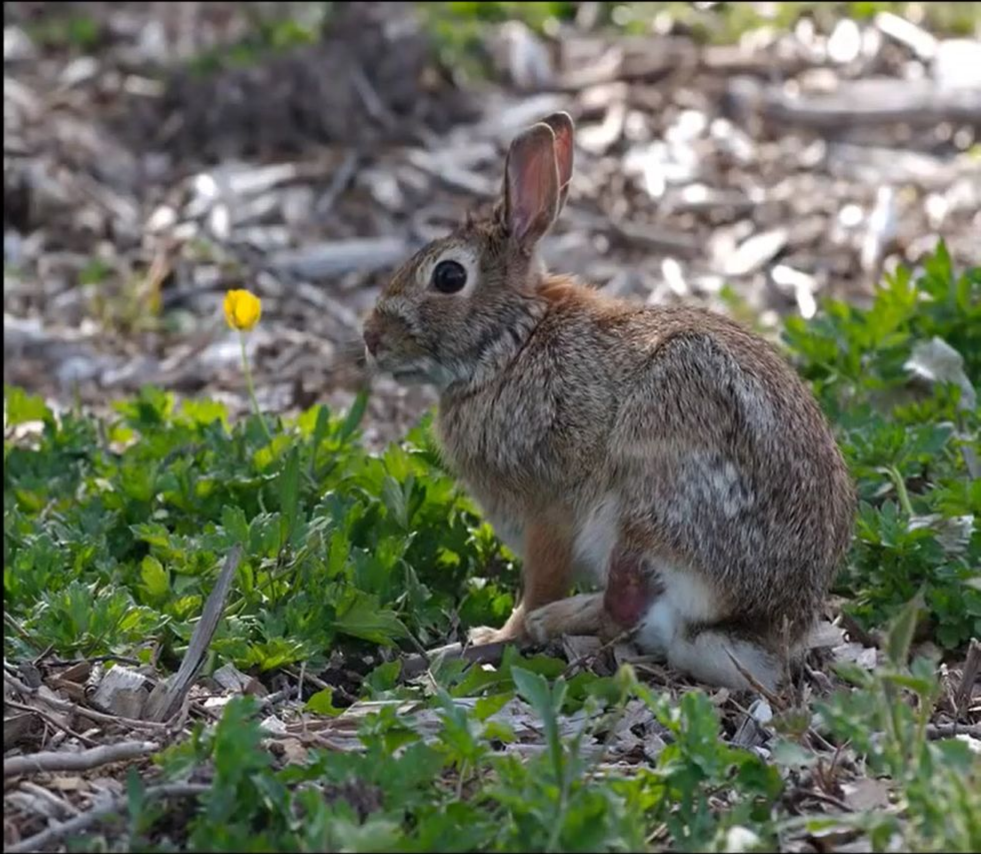

NATURAL WORLD
