# Supplementary material for: Novel approach to delivering pro-environmental messages significantly shifts norms and motivation, but children are not more effective spokespeople than adults
Source: PLoS One. 2021 Sep 8;16(9):e0255457. doi: 10.1371/journal.pone.0255457 (PMC8425541; doi:10.1371/journal.pone.0255457)
Supplement: S1 Images — (ZIP) [file pone.0255457.s006.zip › Slideshows/Study 2 Slideshow Kids.pdf]

"Keep remaking, keep reusing, to keep the world clean."
-Madeline L. 4th grade

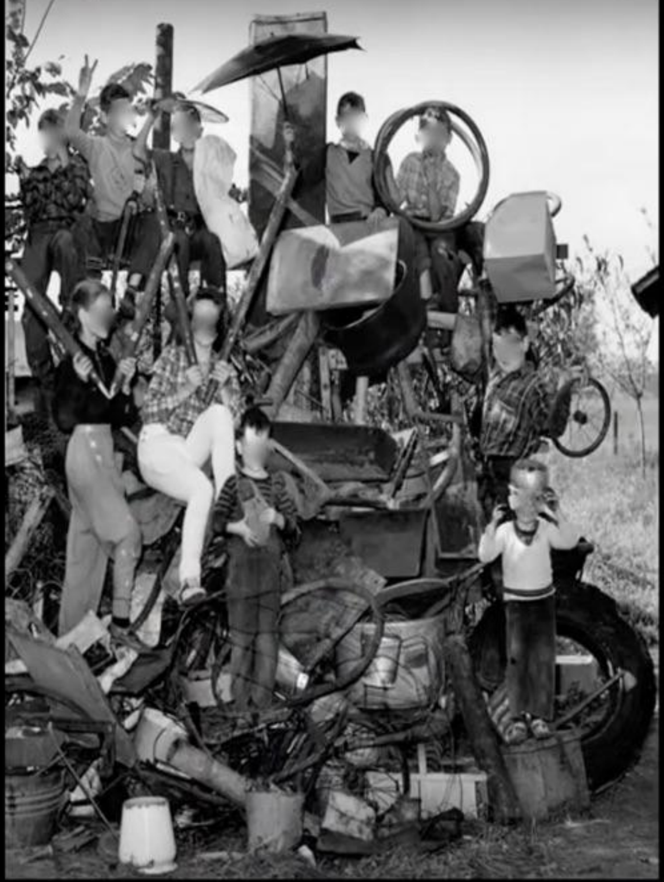

HERITAGE

"Everything we need to live is here on earth. We just have to share it so we can all use it."

-Annie B. 5th grade

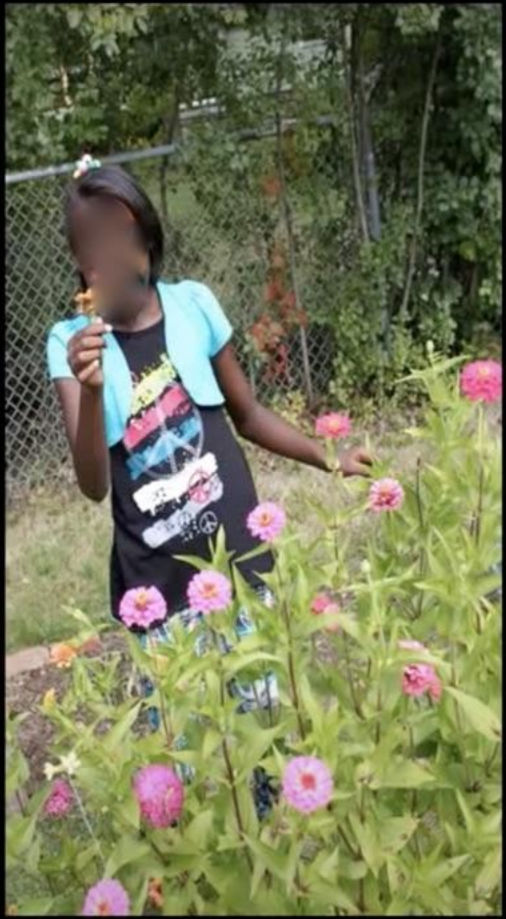

NEIGHBORS

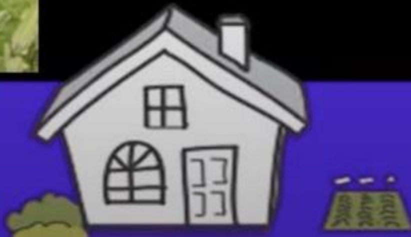

"Our planet will stay healthy when we create as little trash as possible."
-Justin B. 4th grade

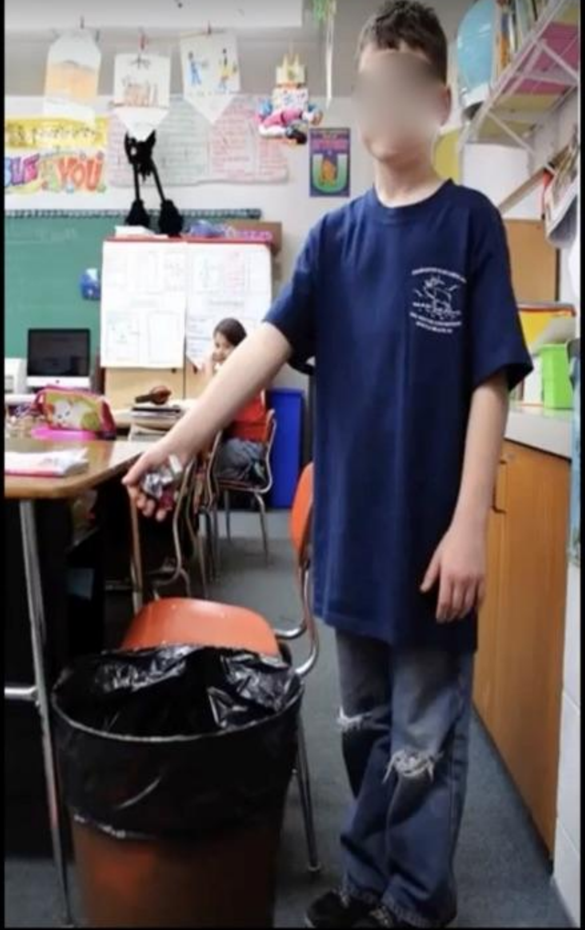

“I think rivers are beautiful”
-Elizabeth S. 6th grade

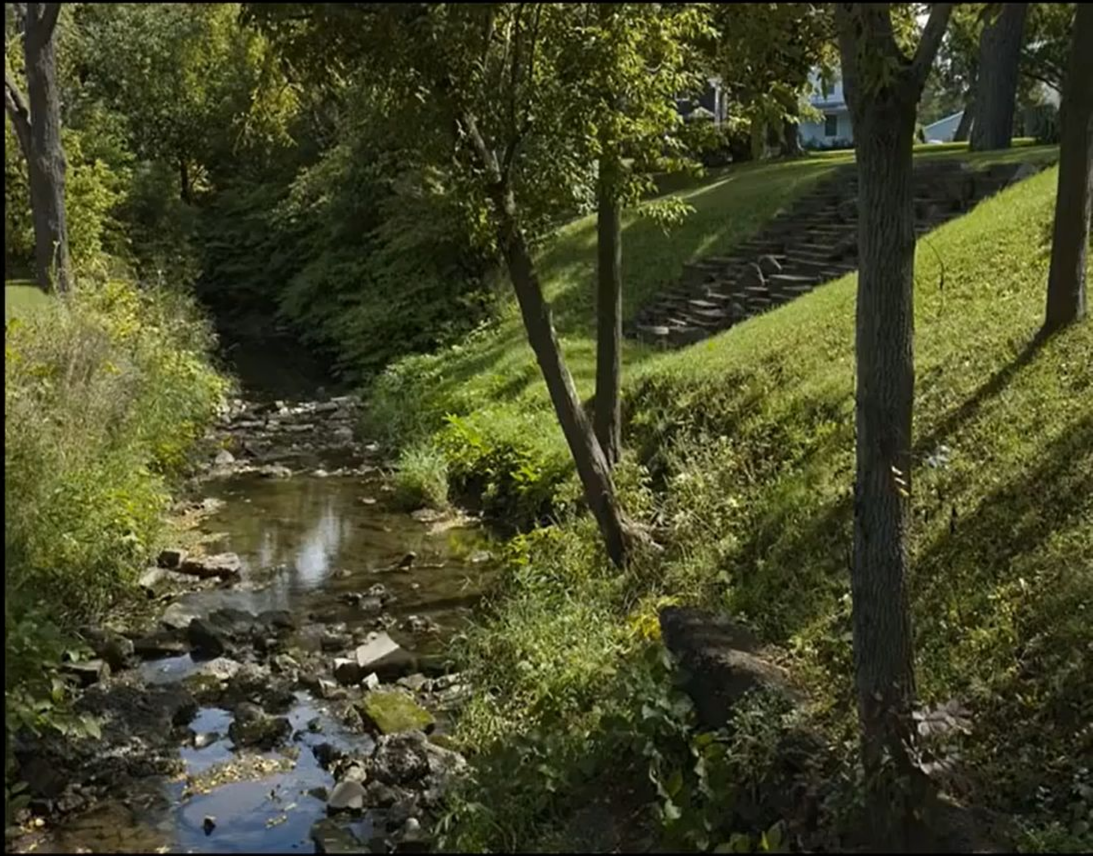

NATURAL WORLD

"We will build this community by bringing people together."
-Ashley W. 11th grade

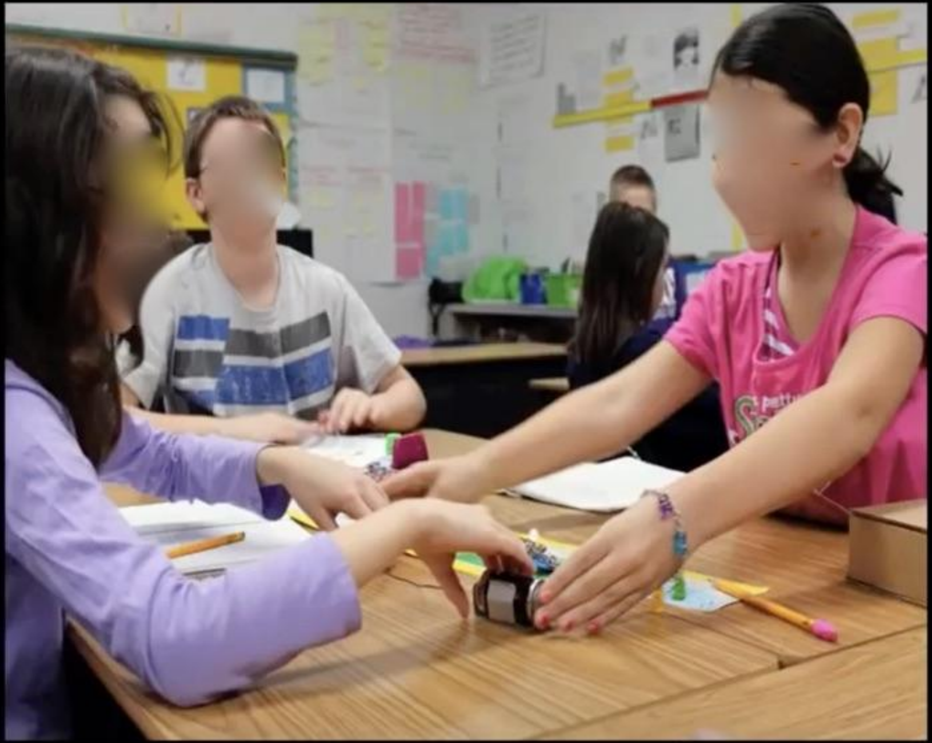

NEIGHBORS

“Get outside and ride your bike! Keep the air clean”
-George H. 4th grade

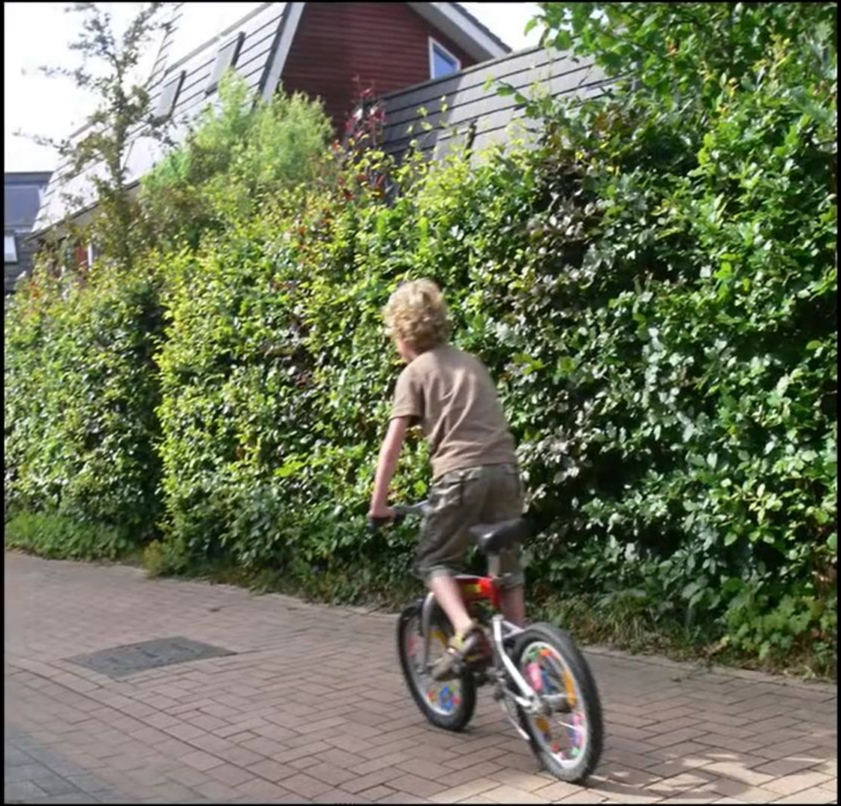

NEIGHBORS

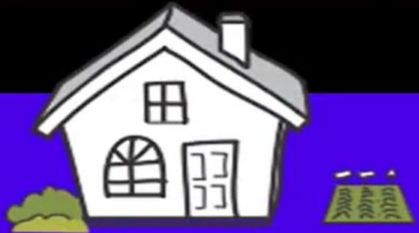

"What affects your community affects you."
-Anna C. 5th grade

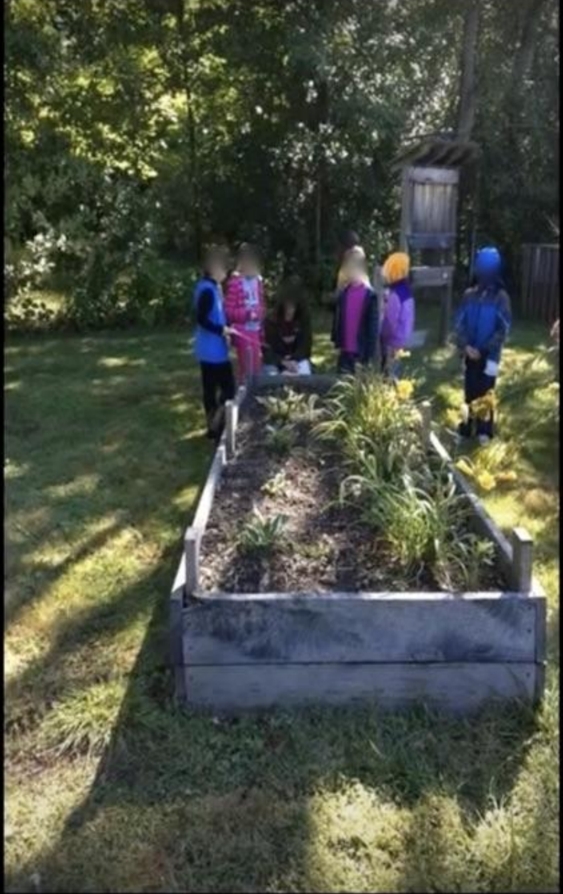

NEIGHBORS

"Sustainability is what we need to be happy and healthy." -Josh K. 5th grade

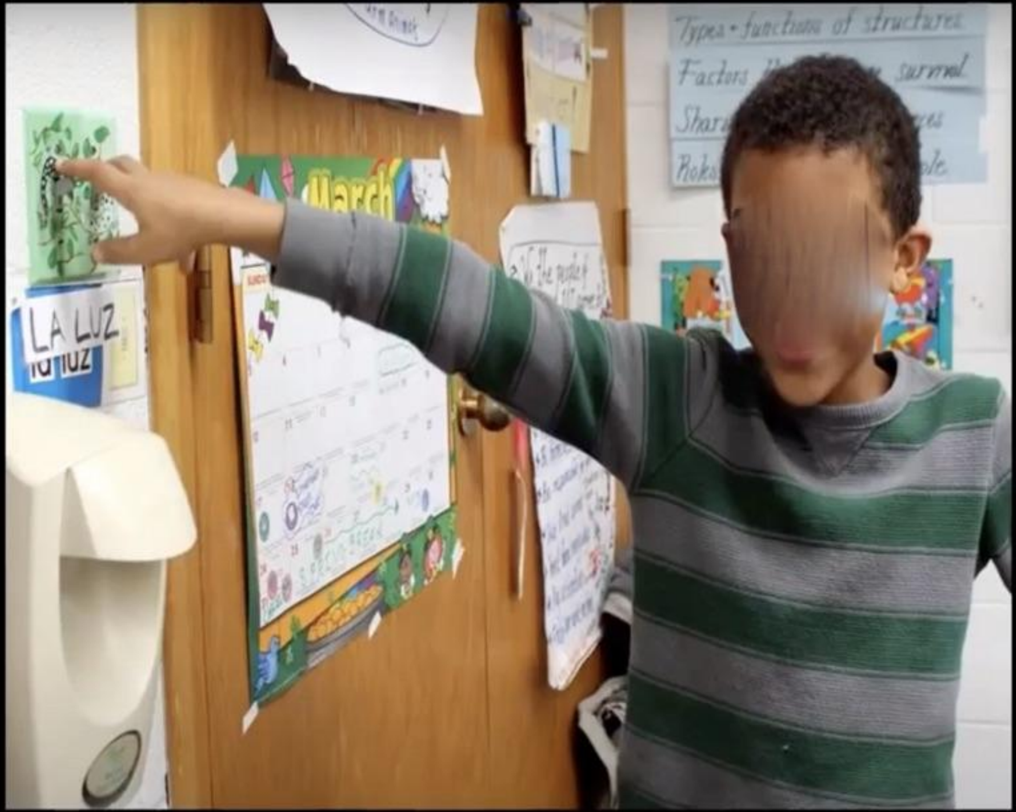

NEIGHBORS

"We need a place to be outside and have fun."
-Laura H. 3rd grade

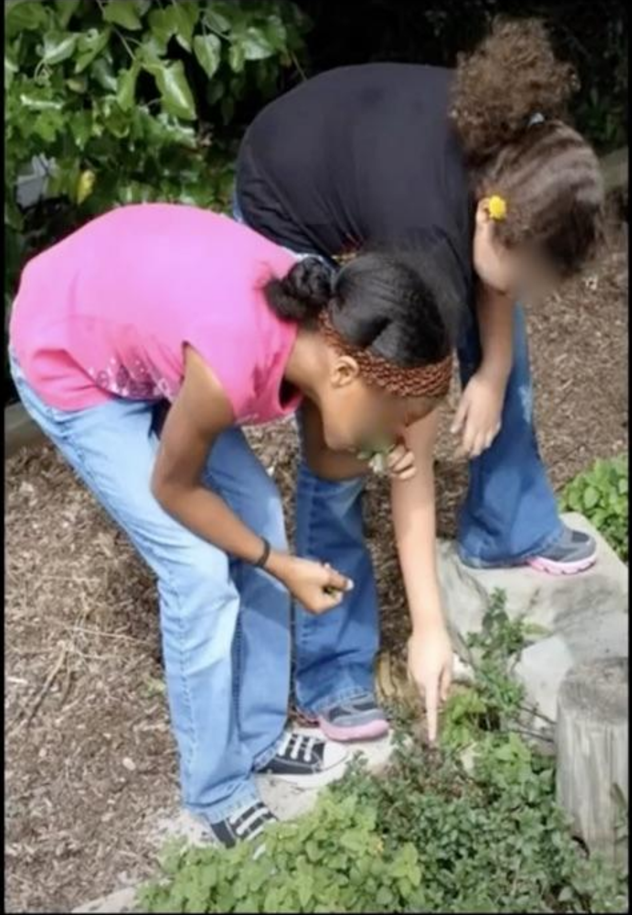

NEIGHBORS

“I enjoy watching the fish swim down the stream.”
-Nicole J. 5th grade

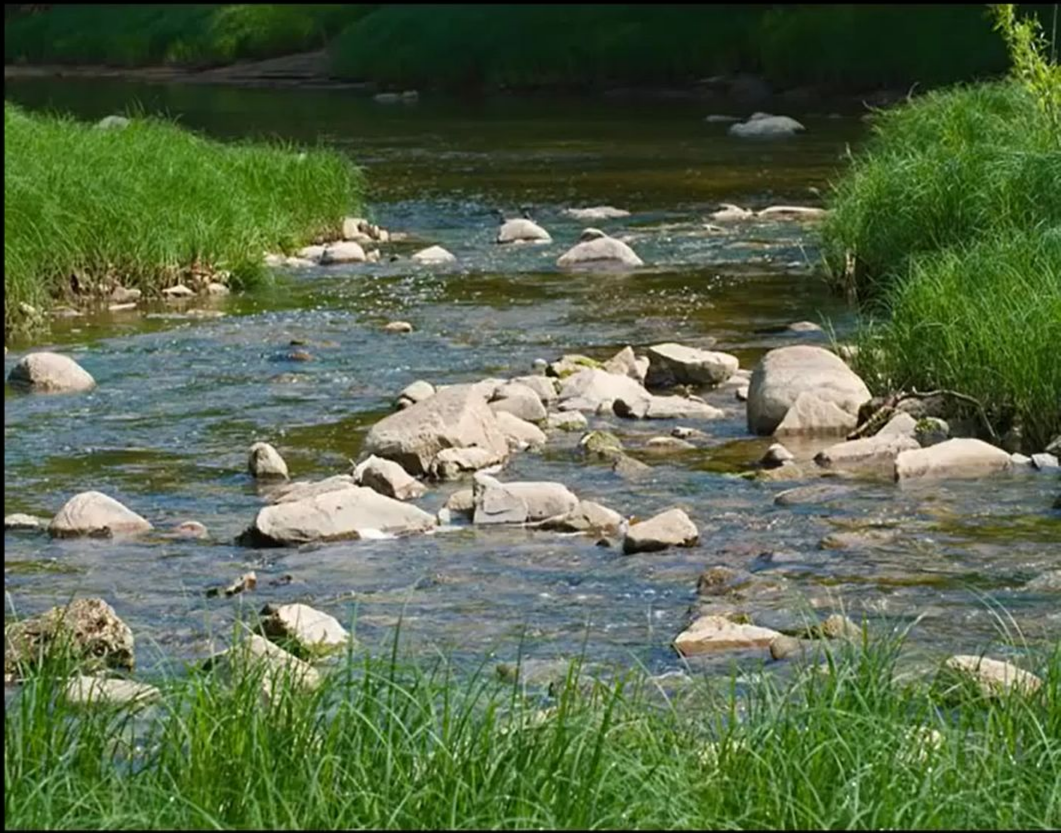

NATURAL WORLD

"I like learning about the environment in school"
-Lori G. 3rd grade

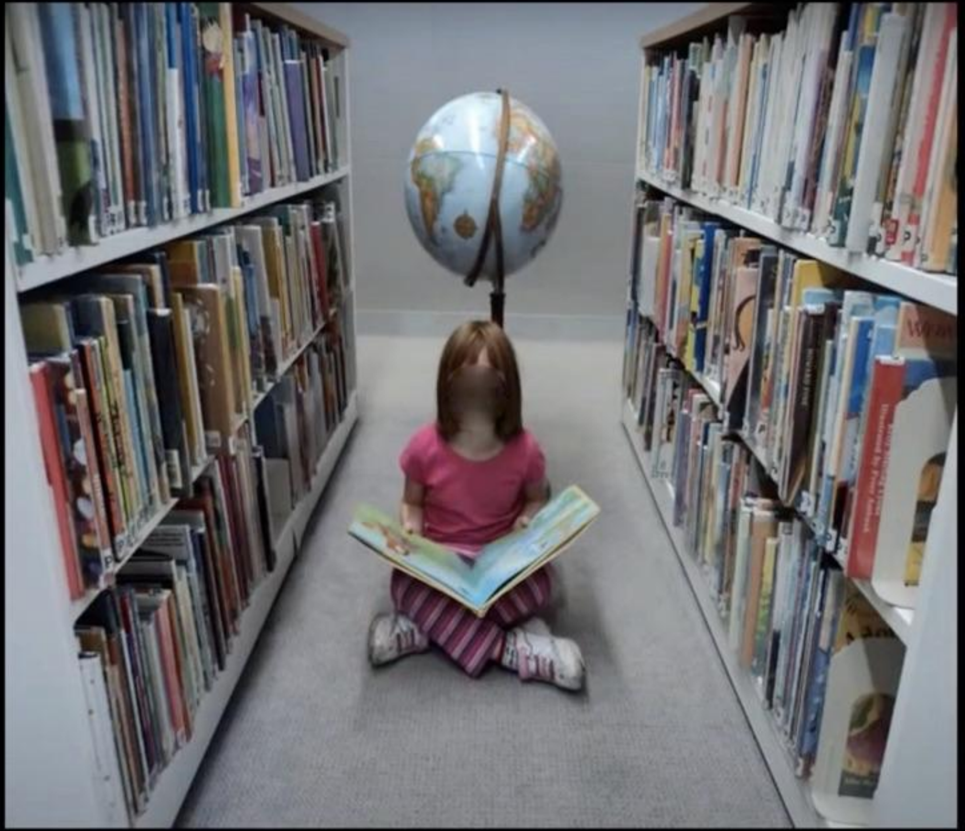

NEIGHBORS

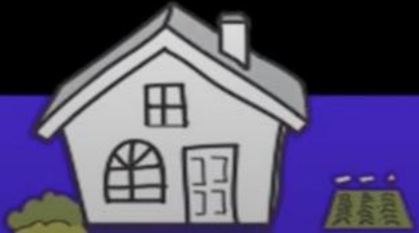

"It's good for our school to use renewable energy." -Phil M. 6th grade

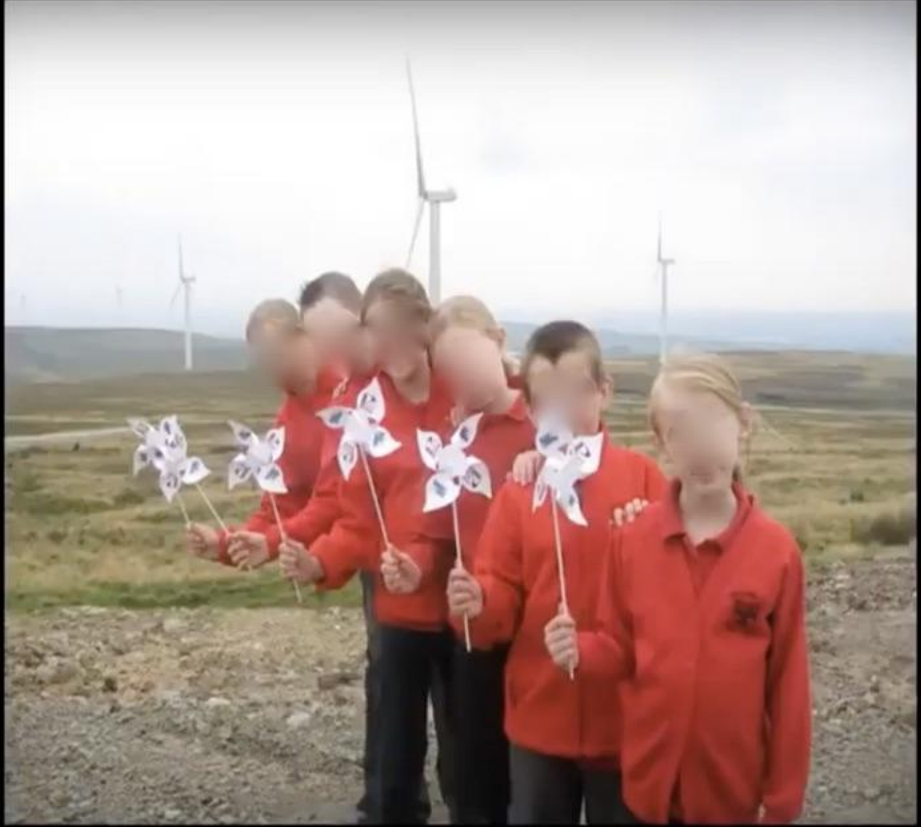

NEIGHBORS

“We should protect the earth because our land is for everyone: humans, plants, and animals.”
-Bobby M. 5th grade

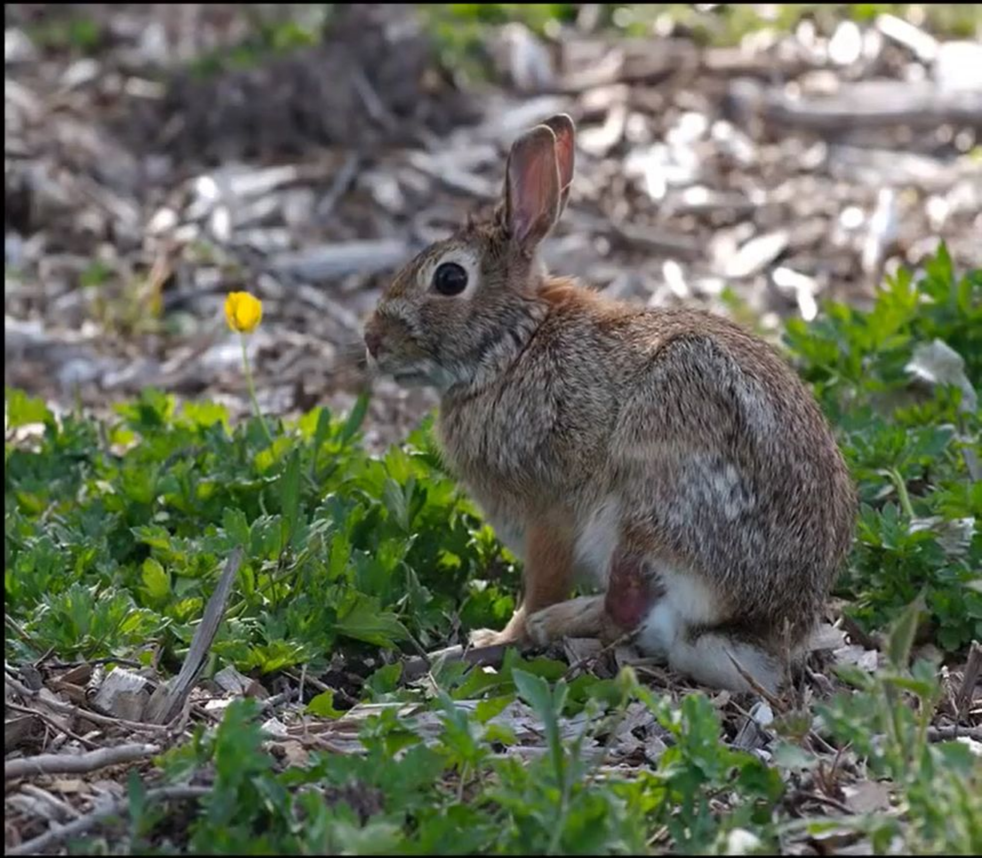

NATURAL WORLD
