# Supplementary material for: Novel approach to delivering pro-environmental messages significantly shifts norms and motivation, but children are not more effective spokespeople than adults
Source: PLoS One. 2021 Sep 8;16(9):e0255457. doi: 10.1371/journal.pone.0255457 (PMC8425541; doi:10.1371/journal.pone.0255457)

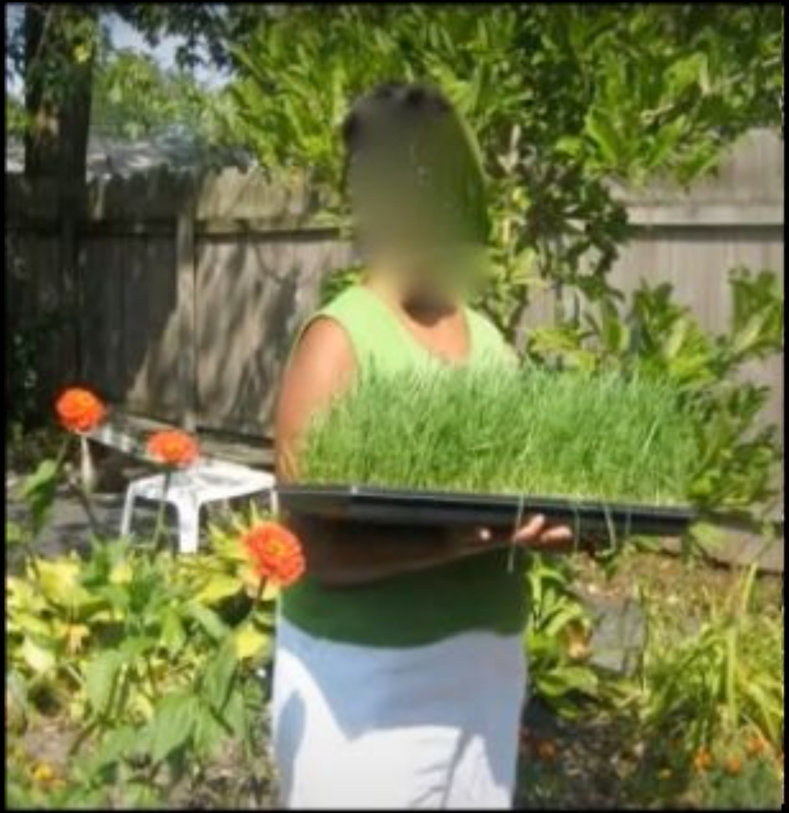

NEIGHBORS

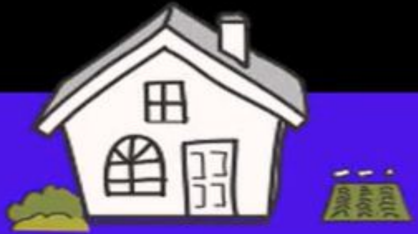

"You need to think about how the planet will be for kids in the future."

-Madeline L.

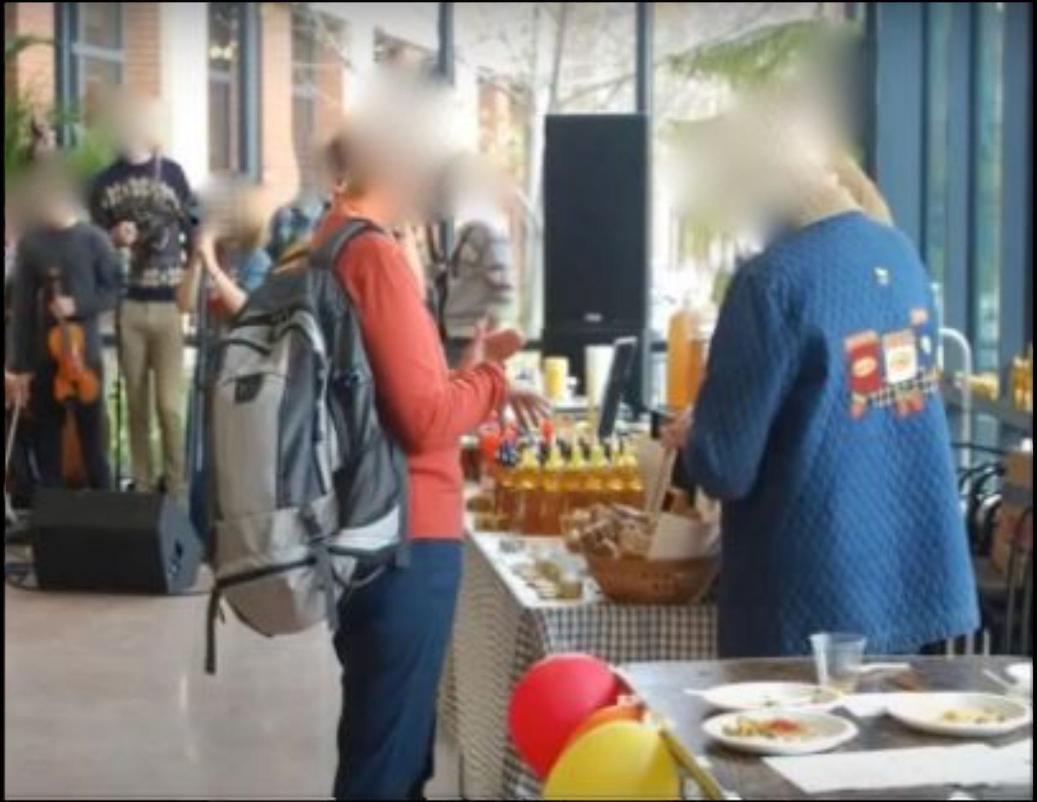

NEIGHBORS

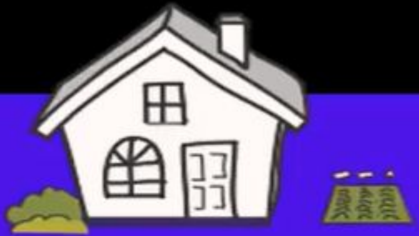

"You should do something to protect the forests."
-Annie B.

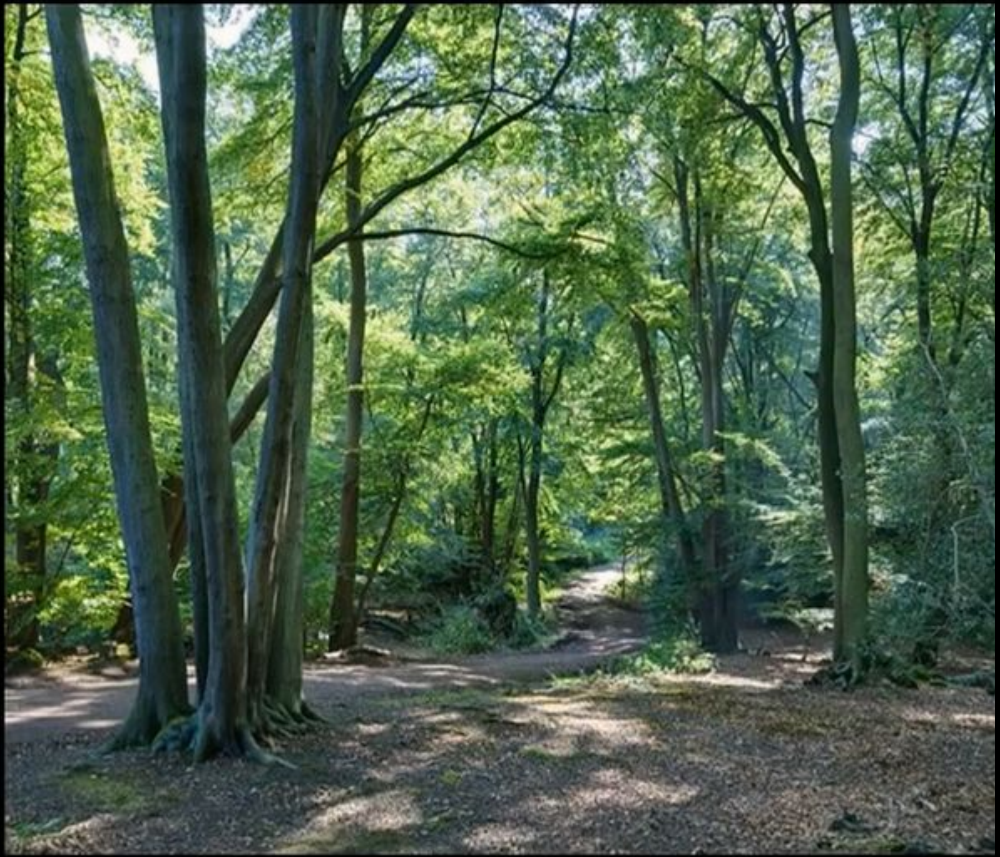

NATURAL WORLD

"You should make changes in your life that help us waste less."
-Justin B.

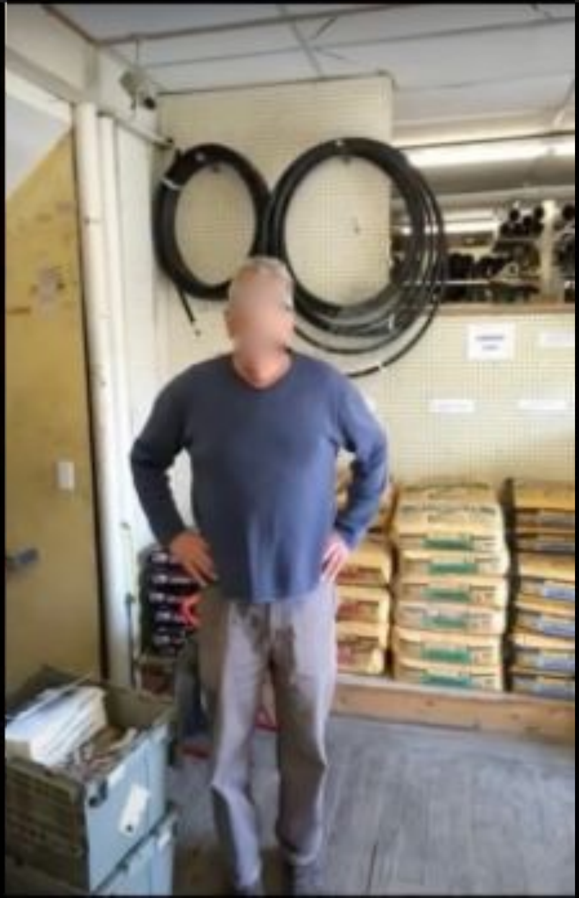

NEIGHBORS

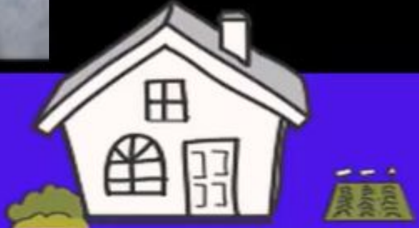

"I think businesses need to be polluting less."

-Elizabeth S.

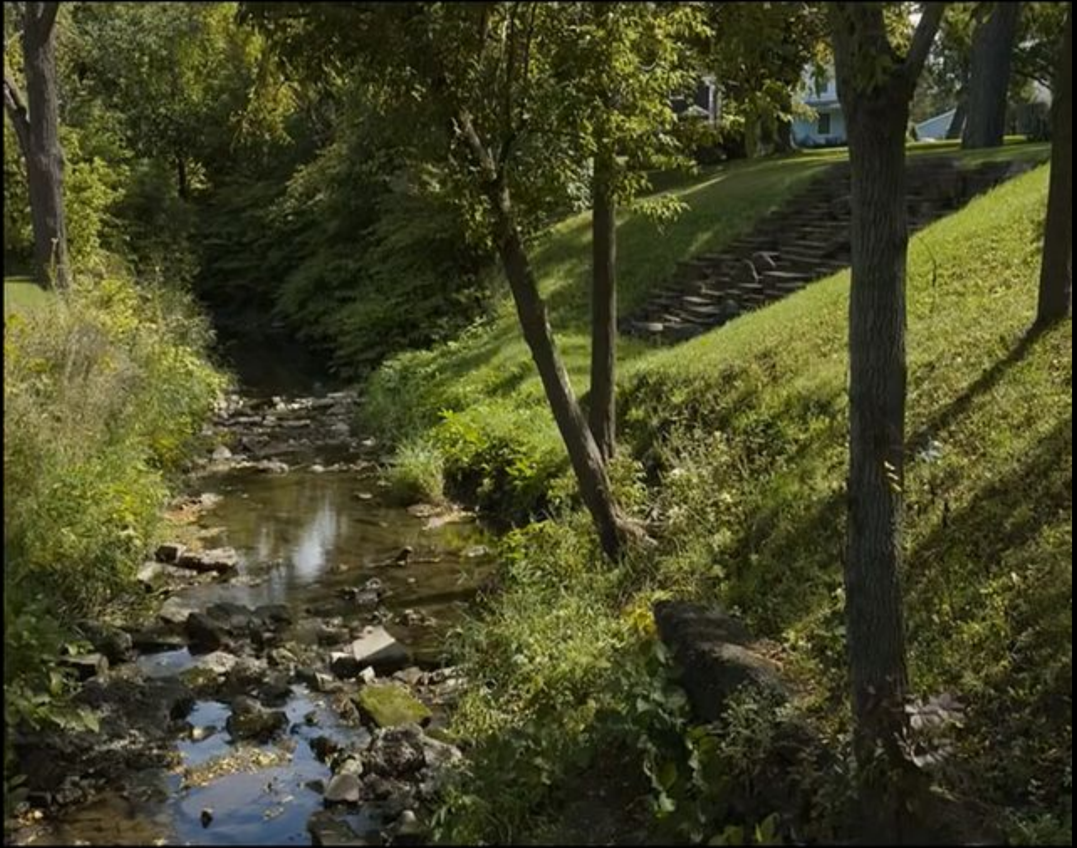

NATURAL WORLD

“You should garden in your yard so you can have fresh food.“
-Ashley W.

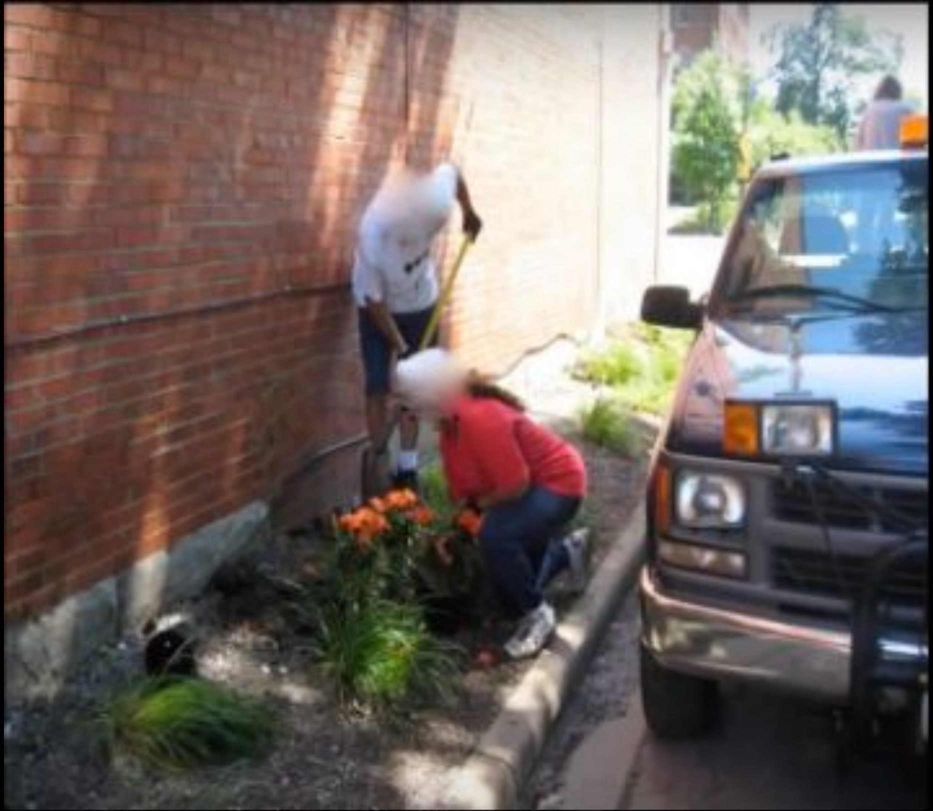

NEIGHBORS

"You have to do more to stop hurting the environment."
-George H.

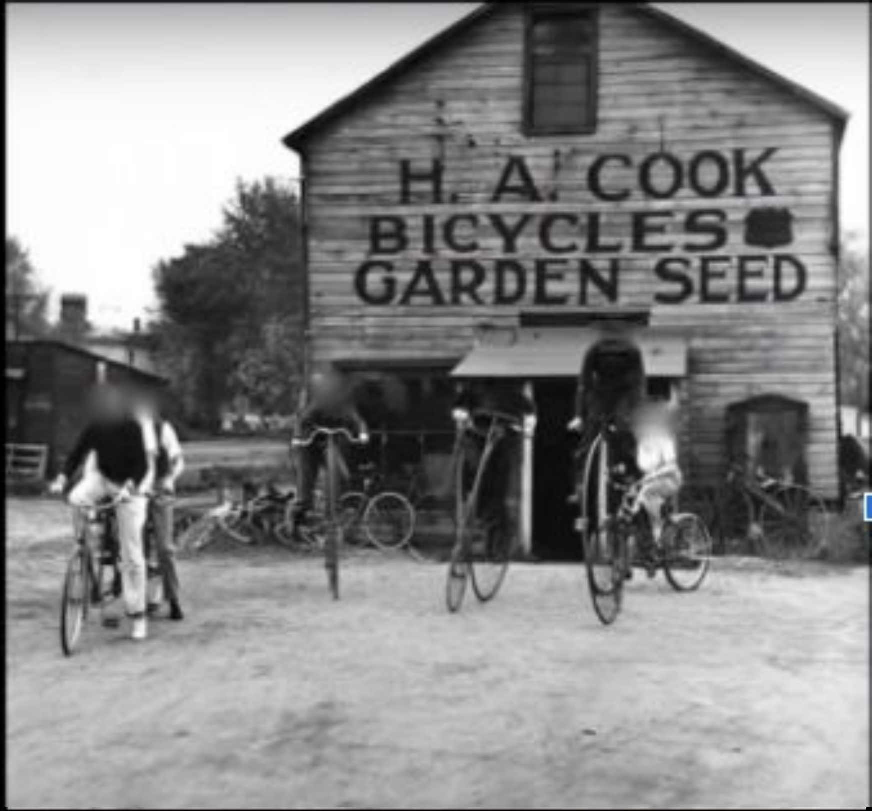

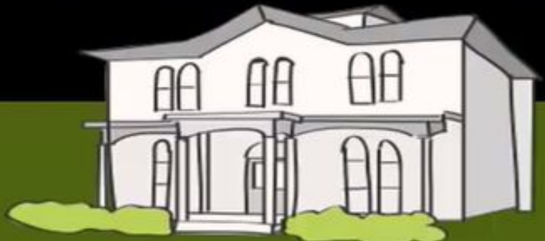

HERITAGE

"You should work to help our community and environment."
-Anna C.

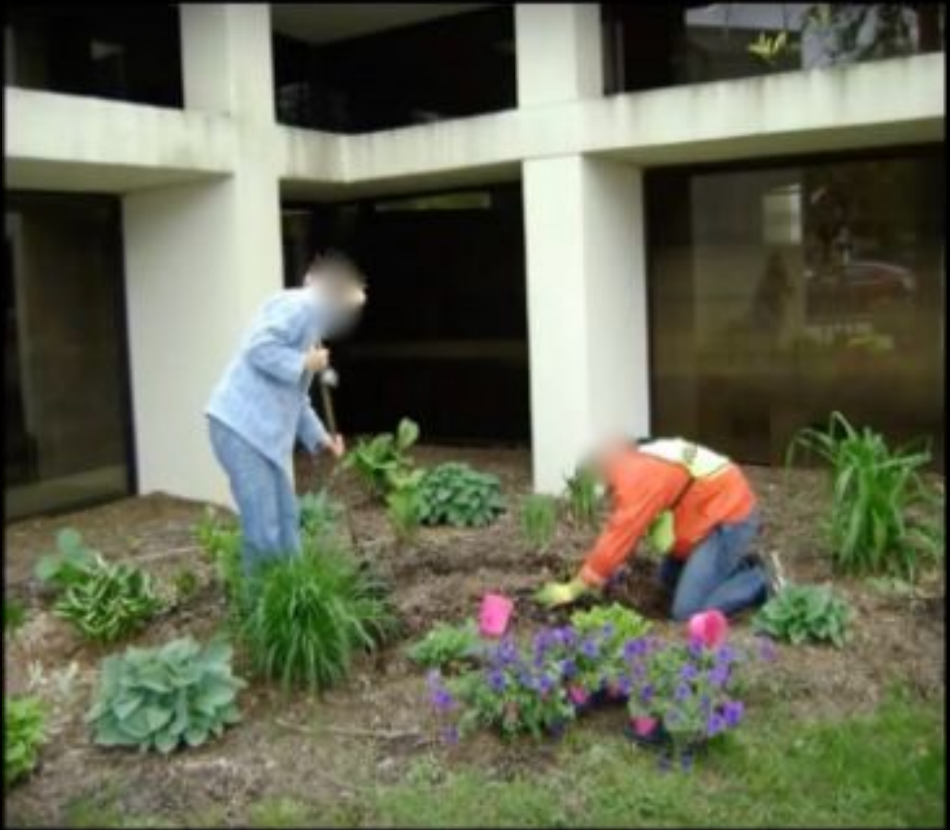

NEIGHBORS

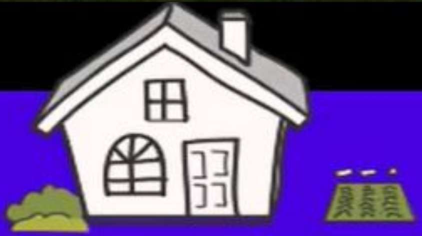

"People who use less water should be rewarded."
-Jack H.

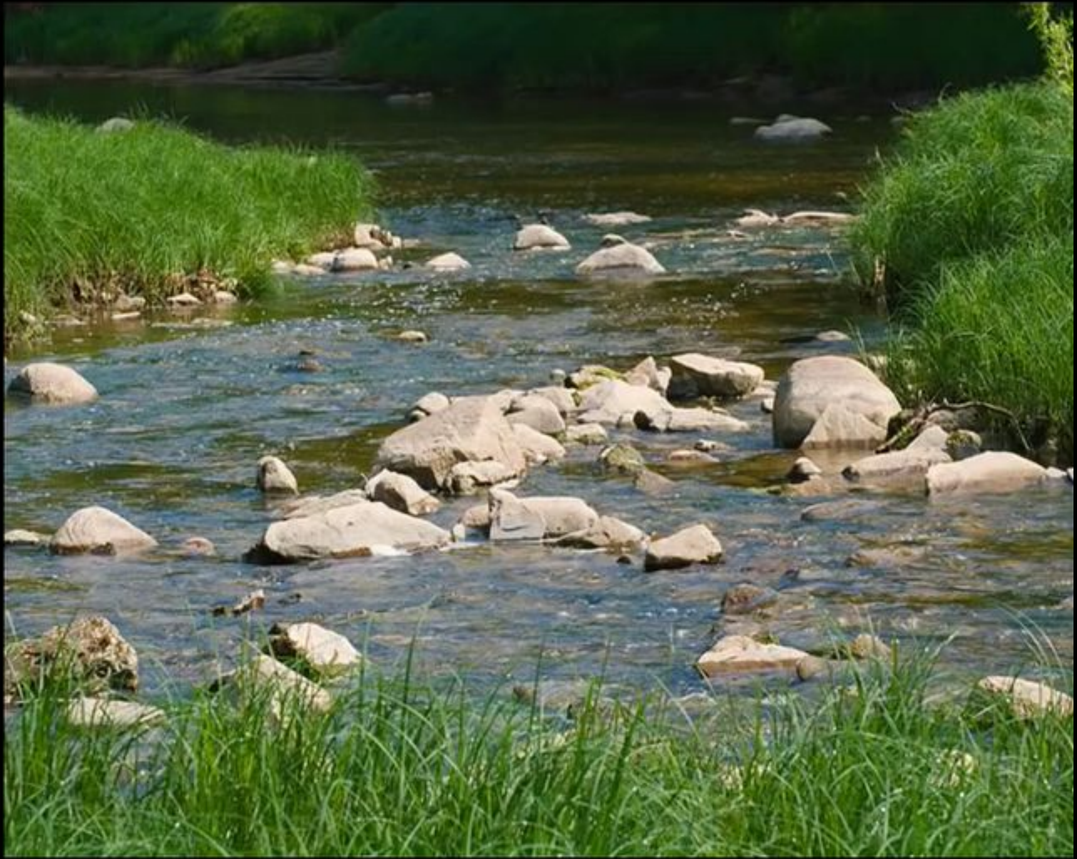

NATURAL WORLD

"You should care more about your electricity use."

-Josh K.

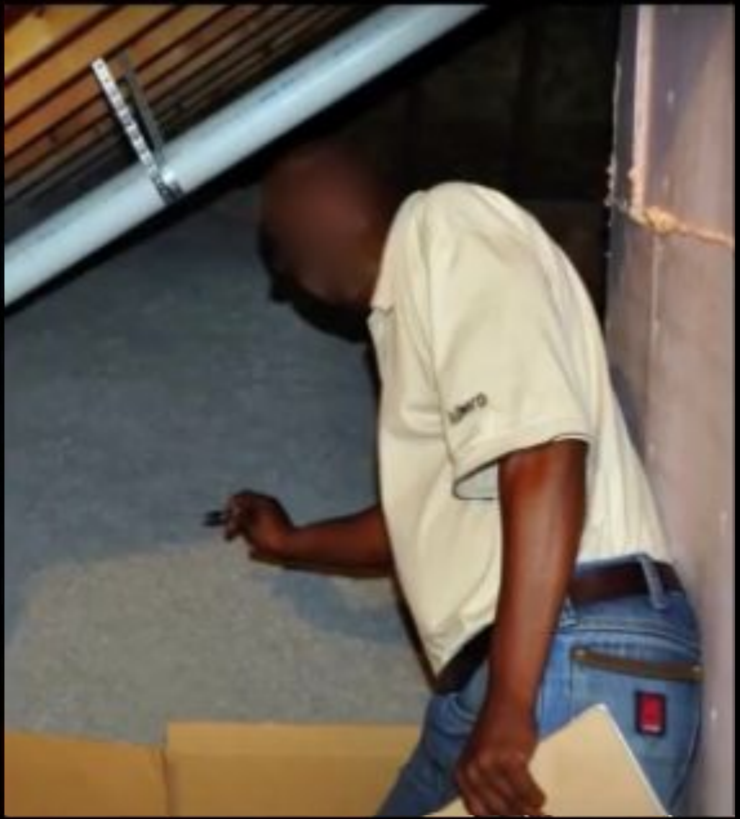

NEIGHBORS

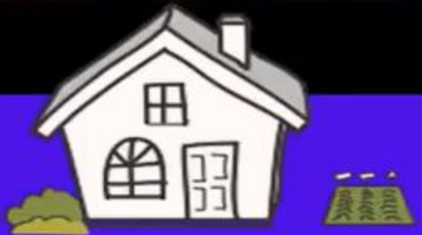

“You’ve got to work towards having cleaner air.“

-Laura H.

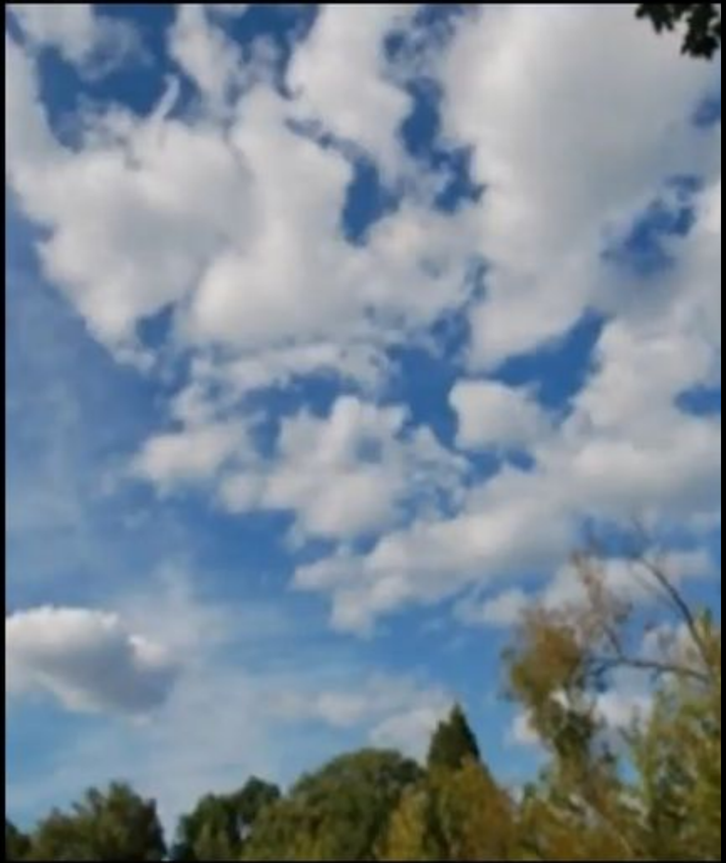

NATURAL WORLD

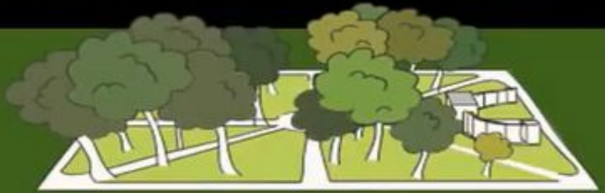

“You should ride your bike more often.“
-Jeremy C.

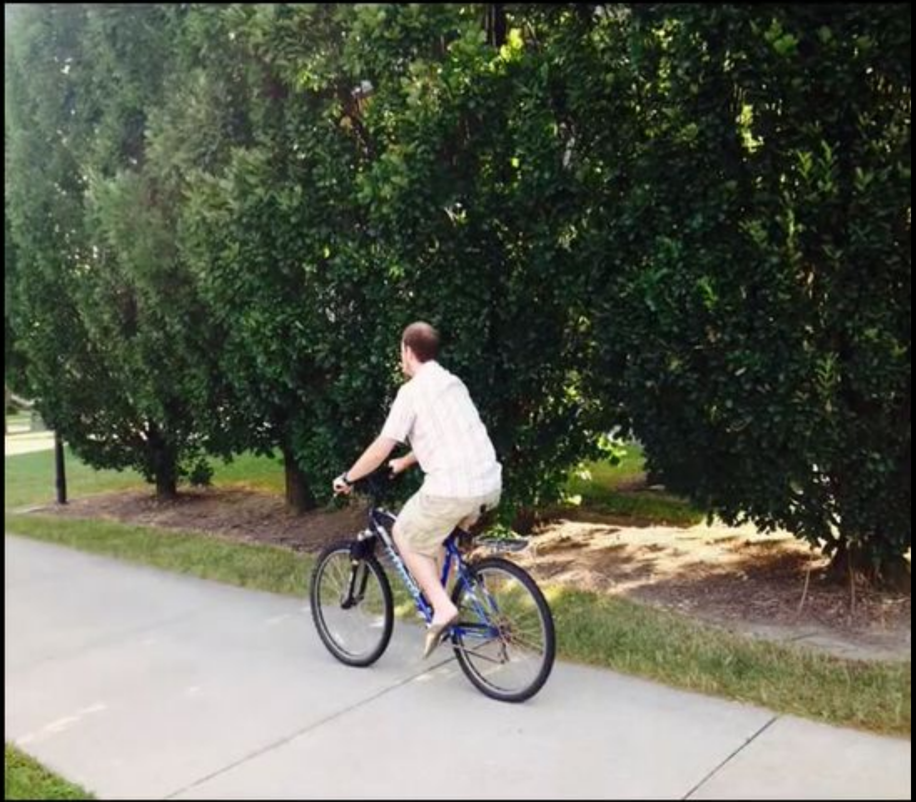

NEIGHBORS

"You need to make sure not to pollute the water."
-Nicole J.

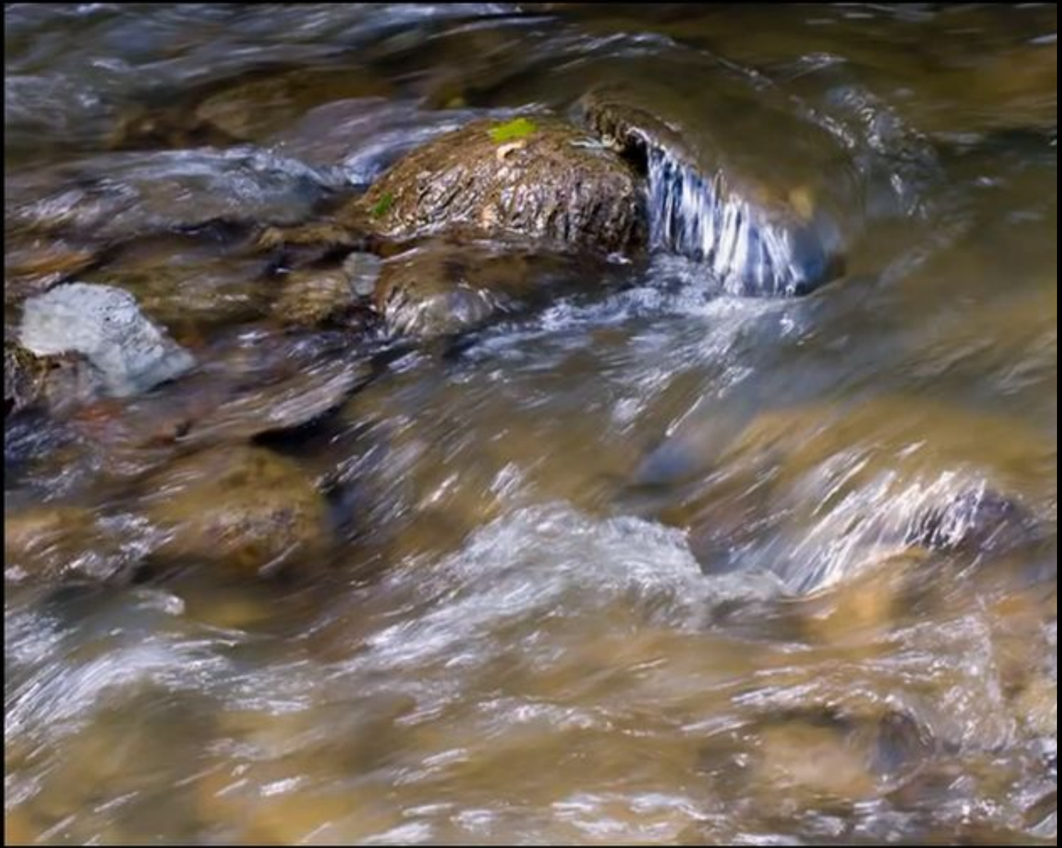

NATURAL WORLD

"You should help protect endangered animals."

-Lori G.

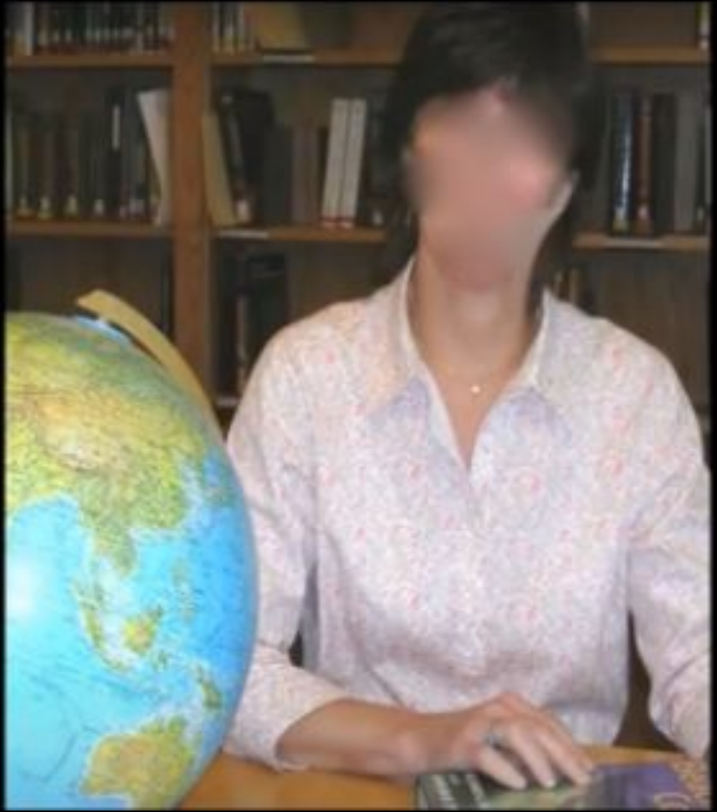

NEIGHBORS

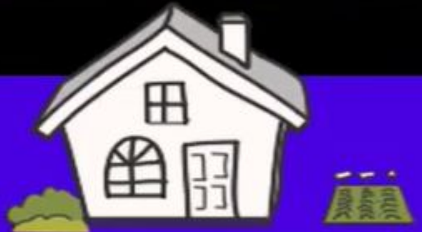

“You should be using more renewable energy.“
-Phil M.

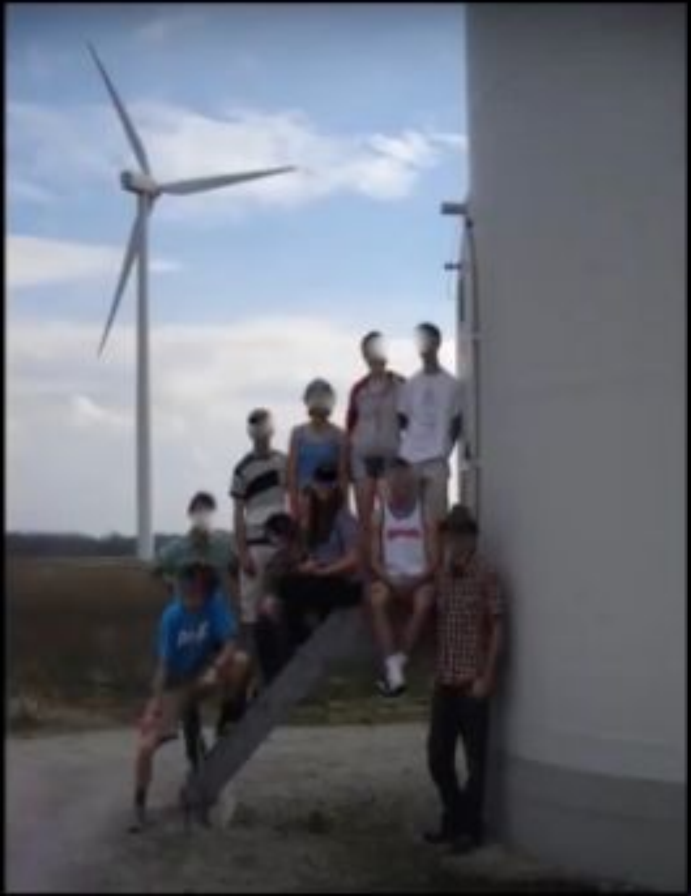

NEIGHBORS

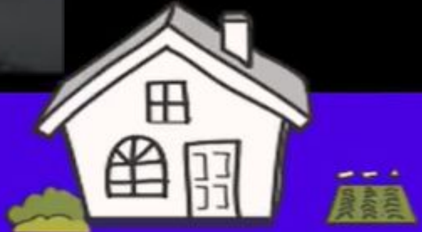

"Our land is for everyone: humans, plants, and animals. You should be sharing it.“
-Bobby M.

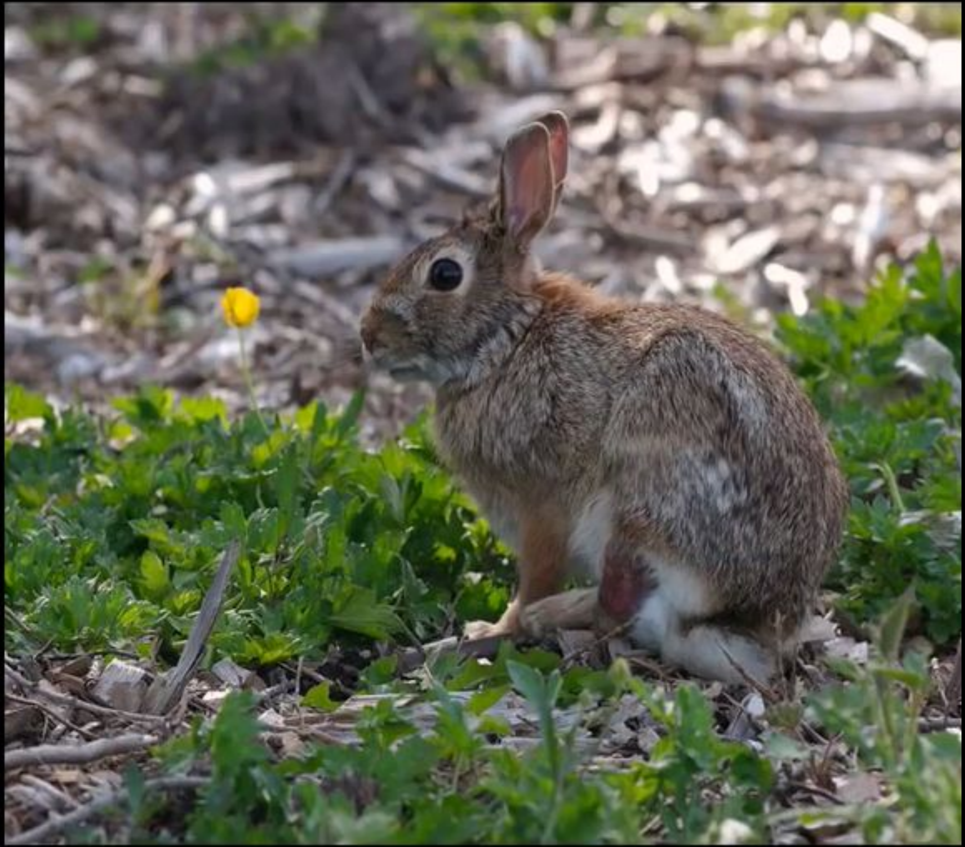

NATURAL WORLD

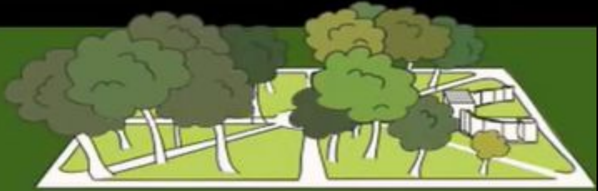

Supplement: S1 Images — (ZIP) [file pone.0255457.s006.zip › Slideshows/Study 1 Slideshow Adults.pdf]
